# miR‐124‐dependent tagging of synapses by synaptopodin enables input‐specific homeostatic plasticity

**DOI:** 10.15252/embj.2021109012

**Published:** 2022-07-25

**Authors:** Sandra Dubes, Anaïs Soula, Sébastien Benquet, Béatrice Tessier, Christel Poujol, Alexandre Favereaux, Olivier Thoumine, Mathieu Letellier

**Affiliations:** ^1^ University of Bordeaux CNRS Interdisciplinary Institute for Neuroscience UMR 5297 Bordeaux France; ^2^ University of Bordeaux CNRS INSERM Bordeaux Imaging Center BIC UMS 3420, US 4 Bordeaux France

**Keywords:** homeostatic synaptic plasticity, microRNA, synaptopodin, synaptic tag, synapse‐autonomous, Neuroscience, RNA Biology

## Abstract

Homeostatic synaptic plasticity is a process by which neurons adjust their synaptic strength to compensate for perturbations in neuronal activity. Whether the highly diverse synapses on a neuron respond uniformly to the same perturbation remains unclear. Moreover, the molecular determinants that underlie synapse‐specific homeostatic synaptic plasticity are unknown. Here, we report a synaptic tagging mechanism in which the ability of individual synapses to increase their strength in response to activity deprivation depends on the local expression of the spine‐apparatus protein synaptopodin under the regulation of miR‐124. Using genetic manipulations to alter synaptopodin expression or regulation by miR‐124, we show that synaptopodin behaves as a “postsynaptic tag” whose translation is derepressed in a subpopulation of synapses and allows for nonuniform homeostatic strengthening and synaptic AMPA receptor stabilization. By genetically silencing individual connections in pairs of neurons, we demonstrate that this process operates in an input‐specific manner. Overall, our study shifts the current view that homeostatic synaptic plasticity affects all synapses uniformly to a more complex paradigm where the ability of individual synapses to undergo homeostatic changes depends on their own functional and biochemical state.

## Graphical abstract

Selective regulation of synaptopodin expression at synapses allows for the capture of surface‐diffusing AMPA receptors to support homeostatic synaptic plasticity.

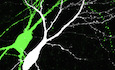

## Introduction

In the face of continuous alterations of neuronal activity, neurons adjust the efficacy of their connections to maintain stable network activity, a process referred to as homeostatic synaptic plasticity (HSP). These compensatory adjustments are achieved at least in part through changes in postsynaptic AMPA receptor (AMPAR) number and function. One major form of HSP is multiplicative synaptic scaling, in which the synaptic strength of every excitatory synapse on a neuron is slowly scaled up or down with the same gain to compensate for prolonged alterations of neuronal firing rate (Turrigiano, 2008). In classical experimental paradigms, chronic treatment of primary neurons with tetrodotoxin (TTX) or bicuculline to inhibit or enhance neuronal activity, respectively, induces a uniform increase or decrease in postsynaptic strengths (Turrigiano *et al*, 1998). This adaptation involves the synthesis of proteins as diverse as glutamate receptors, scaffolding proteins, voltage‐gated ion channels, kinases, secreted factors, and cell adhesion molecules (Fernandes & Carvalho, 2016). *In vivo*, synaptic scaling takes place following sensory deprivation (Desai *et al*, 2002; Goel *et al*, 2006; Keck *et al*, 2013) or during sleep (De Vivo *et al*, 2017; Diering *et al*, 2017) and has been proposed to renormalize synaptic weights while maintaining the relative difference between incoming inputs and consolidating contextual memory. Uniform HSP could thus solve the apparent paradox of having circuits that are both stable and plastic (Vitureira *et al*, 2012; Davis, 2013; Vitureira & Goda, 2013; Turrigiano, 2017; Lee & Kirkwood, 2019; Galanis & Vlachos, 2020).

Yet, the multiplicative nature of synaptic scaling has recently been questioned (Kim *et al*, 2012; Wang *et al*, 2019; Hanes *et al*, 2020) and accumulating evidence points to the existence of a variety of homeostatic mechanisms acting on multiple spatial and temporal scales depending on activity perturbation paradigm, cell type, or developmental stage (Thiagarajan *et al*, 2005; Wierenga *et al*, 2006; Goel & Lee, 2007; Kim & Tsien, 2008; Lee *et al*, 2013, 2014; Lippi *et al*, 2016; Lee & Kirkwood, 2019; Letellier *et al*, 2019). In particular, altering the activity of individual connections revealed that subcellular compartments such as dendritic branches or individual synapses can implement HSP in a relative autonomy, questioning the ability of the cell to maintain synaptic strength differences between inputs (Sutton *et al*, 2006; Hou *et al*, 2008; Beique *et al*, 2011; Letellier *et al*, 2014, 2019; Barnes *et al*, 2017; Li *et al*, 2018). One possible mechanism supporting such synapse autonomy is local protein translation, a process that occurs in remote subcellular compartments including presynaptic terminals and dendritic spines (Hafner *et al*, 2019) and which contributes to local forms of HSP, in particular by regulating the expression of the GluA1 subunit of AMPARs (Sutton *et al*, 2006; Aoto *et al*, 2008; Maghsoodi *et al*, 2008; Letellier *et al*, 2014). Among the actors that can regulate local protein translation, microRNAs (miRNAs) control various forms of HSP (Mellios *et al*, 2011; Tognini *et al*, 2011; Fiore *et al*, 2014; Letellier *et al*, 2014; Rajman *et al*, 2017; Dubes *et al*, 2019; Silva *et al*, 2019). These small noncoding RNAs hybridize to the 3' UTR of multiple target mRNAs and inhibit protein synthesis through translational repression or destabilization of the transcripts (Filipowicz *et al*, 2008; Friedman *et al*, 2009; Soula *et al*, 2018). In neurons, miRNAs can be found in proximity of synapses where they likely respond to activity change and in turn regulate synaptic plasticity (Schratt, 2009; Letellier *et al*, 2014; Sambandan *et al*, 2017; Park *et al*, 2019).

miR‐124 is one of the most enriched miRNAs in the brain and regulates synaptic function, including HSP, through the targeting of the GluA2 AMPAR subunit (Gascon *et al*, 2014; Ho *et al*, 2014; Hou *et al*, 2015). Specifically, the pharmacological blockade of action potentials (APs) and NMDA receptors (NMDARs) in cultured neurons with TTX and D‐APV, respectively, increases miR‐124 expression, and thus, the repression of miR‐124 on GluA2 translation which, unexpectedly, results in synaptic strengthening through the recruitment of GluA2‐lacking AMPARs (Hou *et al*, 2015). Yet, there is also evidence that miR‐124 elevation *in vivo* negatively regulates synaptic transmission and long‐term potentiation (LTP) in the hippocampus with possible implications in spatial learning (Yang *et al*, 2012) and neurological disorders such as epilepsy (Wang *et al*, 2016b), neurodegenerative diseases (Gascon *et al*, 2014; Wang *et al*, 2018), and multiple sclerosis (Dutta *et al*, 2013). Therefore, the mechanisms by which miR‐124 regulates synaptic function still remain elusive. Another target of miR‐124 is synaptopodin (SP) (Elramah *et al*, 2017), an essential component of the spine apparatus that is present only in a subset of spines where it regulates calcium levels and synaptic plasticity, including HSP (Deller *et al*, 2000, 2003; Pierce *et al*, 2000; Holbro *et al*, 2009; Vlachos *et al*, 2009, 2013; Korkotian & Segal, 2011; Chirillo *et al*, 2019). However, the enigmatic expression of SP at some spines but not others suggests that not all synapses are equally competent for synaptic plasticity and makes it hard to reconcile with uniform multiplicative scaling. Interestingly, it was reported that SP clusters emerge inside spines with no obvious transport along dendrites, suggesting that the spine apparatus is assembled on site, possibly involving local protein translation (Konietzny *et al*, 2019).

Here, we investigated whether the regulation of SP and GluA2 translation by miR‐124 could control HSP in hippocampal neurons. Using genetic manipulations to alter SP or miR‐124 expression or to prevent the binding of miR‐124 to SP or GluA2 transcripts, we uncover a synaptic tagging mechanism in which the ability of individual synapses to increase their strength in response to activity deprivation depends on the expression of SP under the control of miR‐124. By building on an original experimental design allowing us to genetically manipulate pre‐ and postsynaptic elements in connected pairs of neurons (Letellier *et al*, 2019), we further demonstrate that miRNA‐dependent local translation of SP supports input‐specific HSP. Overall, we show that SP behaves as a “postsynaptic tag” whose expression at a subset of large and strong synapses is locally controlled by miR‐124 and promotes the “capture” of surface‐diffusing AMPARs and spine growth.

## Results

### TTX‐induced activity deprivation leads to the synaptic recruitment of AMPARs and SP in a nonuniform manner

We first checked whether chronic activity blockade of dissociated neurons using TTX altered the synaptic expression of AMPARs and SP (Turrigiano *et al*, 1998; Gainey *et al*, 2009; Vlachos *et al*, 2013). Because culturing rat hippocampal neurons in neurobasal‐containing medium occluded TTX‐induced HSP in our conditions, most likely through inhibiting action potentials (APs) and spontaneous synaptic activity (Appendix Fig S1A–G), we opted for a culture medium that better fits physiological conditions and supports both neuronal activity and maturation, namely BrainPhys (Bardy *et al*, 2015; Appendix Fig S1, see Materials and Methods).

**Figure 1:**
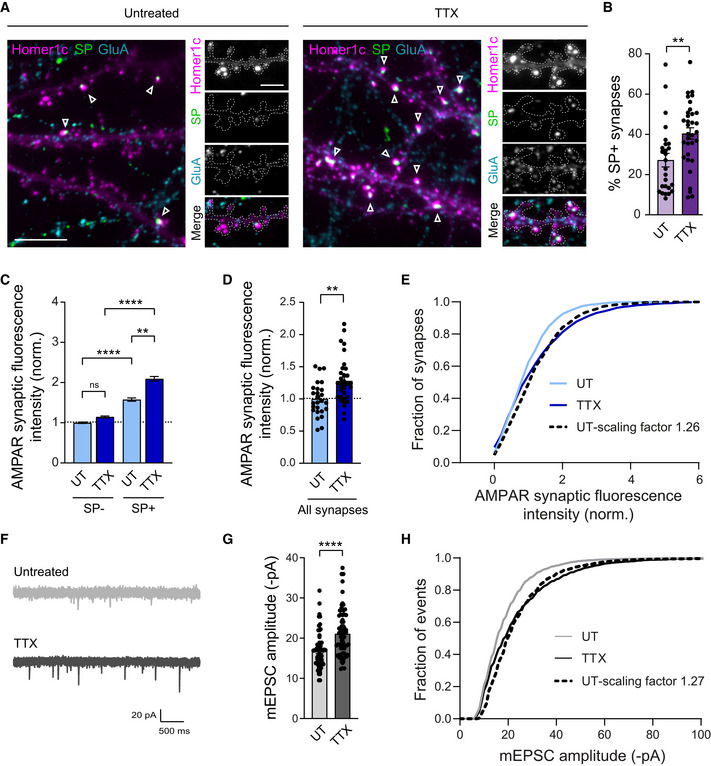
Cultured hippocampal neurons exhibit nonuniform homeostatic synaptic plasticity upon TTX treatment Homer1c‐GFP signal (magenta) and immunostaining for surface AMPARs (cyan) and SP (green) in neurons treated for 48 h with TTX, or untreated (UT). Scale bars: 10 μm (low magnification), 5 μm (insets). Dotted lines indicate the outline of dendrites. Arrowheads indicate SP^+^ synapses.Percentage of SP^+^ synapses for untreated (UT) and TTX‐treated neurons (dot plots represent different cells; UT: *n* = 26; TTX: *n* = 33, *n* indicates the number of cells, from two cultures). % SP^+^ spines: **P = 0.019 (Mann Whitney test).AMPAR synaptic fluorescence intensity for SP^+^ vs. SP^−^ synapses in UT and TTX‐treated neurons. AMPAR synaptic fluorescence intensity was normalized to SP^−^ or small synapses, respectively, from UT condition. (UT: SP^−^, *n* = 1,455, SP^+^, *n* = 516; TTX: SP^−^, *n* = 1,529, SP^+^, *n* = 724, *n* indicates the number of synapses, from two cultures). **P < 0.01, ****P < 0.0001, ns, not significant, *P* > 0.05 (Kruskal–Wallis test followed by Dunn's multiple comparison test).Average AMPAR synaptic fluorescence intensity in UT or TTX‐treated neurons, regardless of the expression of SP (all synapses; UT: *n* = 26; TTX: *n* = 33, *n* indicates the number of cells, from three cultures). AMPAR intensities were normalized to UT condition. **P = 0.0014 (Mann Whitney test).Cumulative probability distribution of AMPAR synaptic fluorescence intensity for untreated neurons (UT, light blue), 48 h TTX‐treated neurons (TTX, dark blue) and scaled UT neurons (dotted black line, scaled factor = 1.26). The scale factor was obtained by computing the ratio between TTX and UT average values. UT vs. TTX, ****P < 0.0001; UT‐scaled vs. TTX, ***P = 0.0005 (Kolmogorov–Smirnov test).Representative traces of AMPAR‐mediated miniature currents (mEPSCs) recorded from neurons cultured in BrainPhys supplemented with TTX for 48 h (TTX, dark gray), or untreated (UT, light gray).Mean mEPSC amplitudes for each condition (UT: *n* = 65; TTX: *n* = 68, *n* indicates the number of cells, from 10 cultures). ****P < 0.0001 (Mann–Whitney test).Cumulative probability distributions of mEPSC amplitudes for untreated neurons (UT, light gray), TTX‐treated neurons (TTX, black) and untreated scaled to TTX condition (dotted black line, scale factor = 1.27). The scale factor was obtained from the linear regression of the ranked mEPSC amplitudes for UT vs. TTX condition. UT vs. TTX, *P = 0.0412, UT scaled vs. TTX, *P = 0.0316 (Kolmogorov–Smirnov test). Data information: Data are represented as mean ± SEM.

**Figure EV1:**
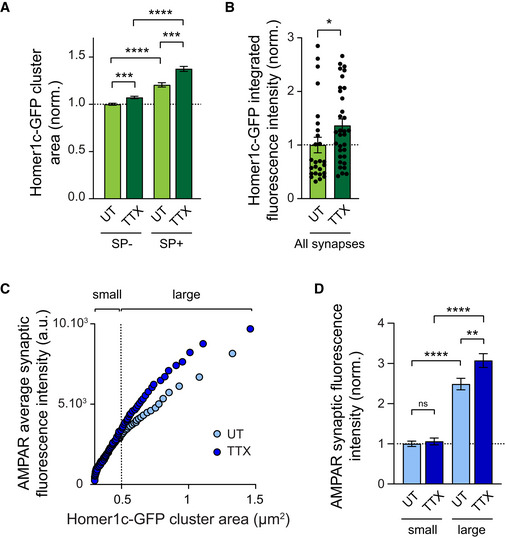
Synapse size correlates with SP expression and predicts synaptic AMPAR content depending on network activity Area of Homer1c‐GFP clusters containing SP (SP^+^) or not (SP^−^) from neurons treated with TTX or left untreated (UT). Homer1c‐GFP cluster area was normalized to untreated SP^−^ synapse condition (SP^−^: UT, *n* = 1,455, TTX, *n* = 1,529; SP^+^: UT, *n* = 516, TTX, *n* = 724; *n* indicates the number of synapses from three cultures). ***P < 0.001, ****P < 0.0001 (Kruskall–Wallis test followed by Dunn's multiple comparison test).Integrated fluorescence intensity of Homer1c‐GFP clusters in UT or TTX‐treated neurons, regardless of the expression of SP (all synapses) (UT: *n* = 26; TTX: *n* = 33, *n* indicates the number of cells, from three cultures). Homer1c‐GFP cluster area was normalized to untreated condition. *P = 0.019 (Mann–Whitney test).Plots showing synaptic AMPAR fluorescence intensity vs. synapse size for neurons treated with TTX (dark blue) or untreated (UT, light blue). The two curves were fitted using linear equations and the convergence of the traces to a common fit was tested using the extra sum of squares *F* test. The *F* test indicates that the traces are best fitted by two divergent linear models (*P* < 0.0001). Small and large synapses were defined according to the area of Homer1c‐GFP clusters with a cut‐off set at 0.5 μm^2^.AMPAR synaptic fluorescence intensity at small vs. large synapses in UT and TTX‐treated neurons. AMPAR synaptic fluorescence intensity was normalized to small synapses from UT condition (UT: SP^−^, *n* = 1,455, SP^+^, *n* = 516; TTX: SP^−^, *n* = 1,529, SP^+^, *n* = 724, *n* indicates the number of synapses, from three cultures). **P < 0.01, ****P < 0.0001, ns, not significant, *P* > 0.05 (Kruskal–Wallis test followed by Dunn's multiple comparison test). Data information: Data represent mean ± SEM.

Hippocampal neurons cultured in this medium were transfected at DIV10 with Homer1c‐GFP as a postsynaptic marker and immunostained at DIV15 for endogenous SP and surface AMPARs using an antibody raised against the GluA2 subunit that also recognizes GluA1 (Fig 1A and B, and Appendix Figs S1F and S2). Under basal conditions, ∼25% of postsynapses contained SP clusters (Fig. 1A and B), consistent with previous reports showing the accumulation of SP at the neck of a small fraction of spines in hippocampal pyramidal neurons (Deller *et al*, 2003; Orth *et al*, 2005; Vlachos *et al*, 2009). Synapses that were immunopositive for SP (SP^+^) were larger in size and displayed higher fluorescence intensity for immunostained AMPARs compared with synapses that were deprived of SP (SP^−^; Figs 1C and EV1A), indicating that the presence of SP is predictive of large and strong synapses (Vlachos *et al*, 2009). Upon 48‐h TTX treatment, neurons displayed a higher percentage of SP+ synapses compared with untreated neurons, accompanied by an increase in synaptic AMPARs and Homer1c‐GFP signals (Figs 1A–D, and EV1A and B). Interestingly, TTX treatment also enhanced the abundance of AMPARs selectively at SP^+^ synapses, leaving SP^−^ synapses with unchanged AMPAR content (Fig 1C). Consistent with this finding, TTX treatment induced a significant increase in immunostained AMPARs at large synapses (defined as Homer1c‐GFP cluster area > 0.5 μm^2^) but not at small ones (Homer1c‐GFP cluster area < 0.5 μm^2^; Fig EV1C and D). To investigate whether the increase in synaptic AMPARs abundance (Fig 1D) was multiplicative, we scaled synaptic AMPAR fluorescence intensities from control cells by the same factor (1.26) to match the average synaptic AMPAR fluorescence intensity from TTX‐treated cells. We next compared the cumulative distributions of AMPARs fluorescence intensities from scaled control and TTX‐treated cells and found a significant difference, indicating that HSP was not multiplicative and selectively occurred at synapses with the highest AMPAR content (Fig 1E). Together, these results reveal a nonuniform synaptic recruitment of both SP and AMPARs across synapses during HSP and a selective contribution of the synapses displaying large size and high AMPAR content.

### TTX‐induced activity deprivation leads to a non‐multiplicative increase in synaptic strengths

**Figure EV2:**
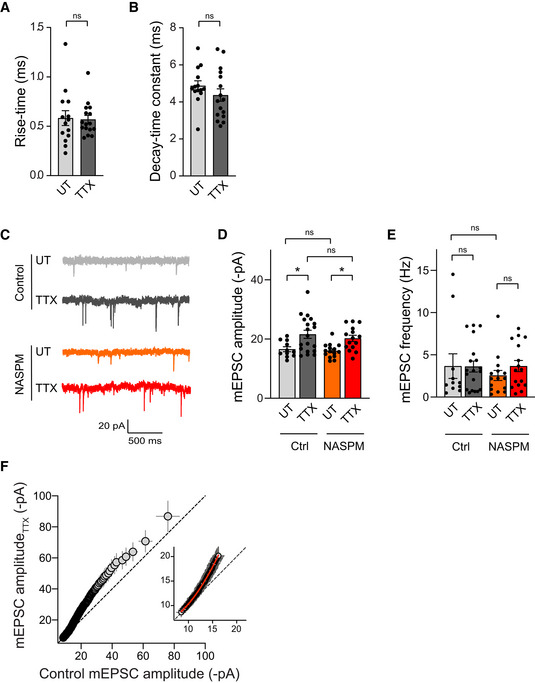
TTX‐induced upscaling is not accompanied by change in mEPSC kinetics or sensitivity to NASPM A, BRise‐time (A) and decay‐time constant (B) for neurons treated with TTX or left untreated (UT) (UT: *n* = 14, TTX, *n* = 16; *n* indicates the number of cells from three cultures). *P* > 0.05, ns, not significant (Mann Whitney test).CRepresentative traces of AMPAR‐mediated mEPSCs from neurons treated with TTX, or untreated (UT) and recorded with or without (control, Ctrl) 10 μM NASPM.D, EMean mEPSC amplitudes (D) and frequencies (E) for each condition (Ctrl: UT, *n* = 11, TTX, *n* = 19; NASPM: UT, *n* = 15, TTX, *n* = 15; *n* indicates the number of cells from three cultures). *P < 0.05, ns, not significant, *P* > 0.05 by two‐way ANOVA test followed by Tukey's multi comparison test (D) or Kruskal–Wallis test (E).FPlot showing the rank‐ordered AMPAR‐mediated mEPSC amplitudes measured in TTX‐treated neurons vs. UT neurons (200 events). The rank‐order plot was obtained by sorting from smallest to largest amplitude in untreated and TTX data and plotting them against each other. The extra sum of squares F test indicates that the first 100 events are better fitted with a second‐order polynomial quadratic curve (in red, TTX = 6.00 − 0.29 × UT + 0.07 × UT^2^; *R* ^2^ = 0.99; ****P < 0.0001) than with a linear regression (not shown, TTX = −5.23 + 1.53 × UT). Data information: Data represents mean ± SEM.

To examine the functional correlate of these molecular changes observed by immunofluorescence, we performed patch‐clamp recordings of AMPAR‐mediated miniature currents (mEPSCs) in the same conditions. TTX‐treated neurons exhibited larger mEPSCs (+4.4 pA higher) compared with untreated neurons (Fig 1F and G), confirming previous results (Turrigiano *et al*, 1998; Sutton *et al*, 2006; Vitureira *et al*, 2011). These currents displayed similar rise time and decay time constant as compared with the untreated condition (Fig EV2A and B) and were insensitive to a selective antagonist of Ca^2+^‐permeable AMPARs, NASPM (Fig EV2C–E). This suggested that AMPARs that were recruited at synapses upon TTX treatment contained the GluA2 subunit (Gainey *et al*, 2009). In addition, TTX treatment did not alter mEPSC frequency (Appendix Fig S1C and E, and Fig EV2C and E), suggesting no change in the presynaptic release probability or number of active synapses. As we did for AMPAR fluorescence intensities (Fig 1E), we next investigated whether the increase in mEPSC amplitudes was multiplicative by comparing the distribution of mEPSC amplitudes from control cells scaled to the TTX condition (factor 1.25) with the distribution obtained for TTX‐treated neurons. Consistent with AMPAR immunostaining, we found that the distributions were significantly different, further supporting that individual synapses were not scaled by a common factor (Fig 1H). Finally, the rank‐ordered mEPSC amplitudes from TTX‐treated cells plotted against rank‐ordered mEPSC amplitudes from control cells confirmed that the scaling factor was not uniform across amplitudes, that is, being close to one for small amplitudes and increasing for larger amplitudes (Fig EV2F). Together, these results suggest that large SP^+^ synapses are the ones displaying the largest increase in mEPSC amplitude upon TTX treatment.

### Surface‐diffusing GluA2‐containing AMPARs get immobilized at SP ^+^ vs. SP^−^ synapses

**Figure 2:**
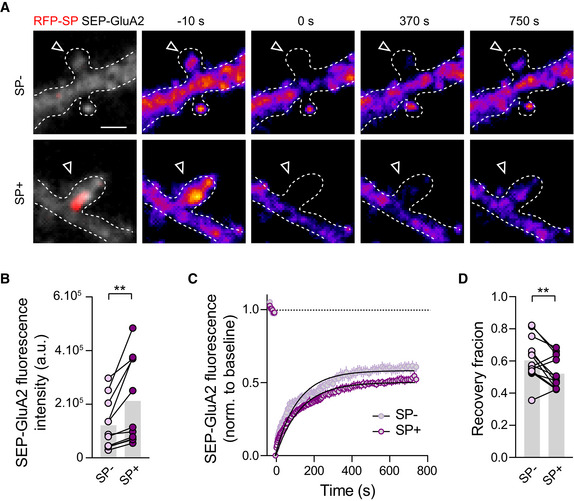
Higher immobilization of surface diffusing AMPARs at SP^+^ vs. SP^−^ synapses Example images of a FRAP experiment: SP^−^ and SP^+^ spines from the same cultured hippocampal neuron transfected with SEP‐GluA2 (gray and color‐coded) + RFP‐SP (red) 10s before, and 0, 370, 750 s after the photobleaching. Scale bars: 1 μm. Dotted lines represent the outline of dendrites. Arrowheads indicate dendritic spines which were targeted for photobleaching.SEP‐GluA2 fluorescence intensity at SP^−^ vs. SP^+^ spines. Each pair of dot plots represents average fluorescence for SP^−^ and SP^+^ spines from a same neuron (four cultures). **P = 0.0061 (two‐tailed paired Student's *t*‐test).Quantification of FRAP dynamics for SP^−^ vs. SP^+^ spines. Recovery curves represent SEP‐GluA2 fluorescence average per cell (*n* = 14 cells, from four cultures). The two traces were fitted using double exponential components equations and the convergence of the traces to a common fit was tested using the extra sum of squares *F* test. The *F* test indicates that the traces are best fitted by two divergent models (*P* < 0.0001).Quantification of the recovery fraction 750 s after the photobleaching. Each pair of dot plots represents average recovery for SP^−^ and SP^+^ spines from a same neuron (four cultures). **P = 0.0067 (Wilcoxon matched‐pairs signed rank test). Data information: Data are represented as mean ± SEM.

To better understand how SP^+^ synapses get enriched in AMPARs relatively to SP^−^ synapses, we next investigated the dynamics of surface GluA2‐containing AMPARs at those two types of synapses by performing fluorescence recovery after photobleaching (FRAP; Ashby *et al*, 2006). We expressed the GluA2 AMPAR subunit containing an N‐terminal super‐ecliptic (SEP) tag (SEP‐GluA2) along with recombinant RFP‐tagged SP (RFP‐SP) in DIV8 cultured hippocampal neurons and performed live fluorescence imaging at DIV10. Under basal conditions, spines containing RFP‐SP clusters exhibited a higher SEP‐GluA2 intensity, compared to spines without RFP‐SP (Fig 2A and B), demonstrating that recombinant SEP‐GluA2 containing AMPARs behaved similarly to endogenous AMPARs, that is, by accumulating preferentially at SP^+^ synapses. In dendritic spines lacking SP, SEP‐GluA2 recovered from photobleaching with a time constant τ = 118.0 s; the recovery was still incomplete after 750 s with an immobile fraction of ∼42% (Fig 2C and D), consistent with the synaptic turnover of surface diffusing GluA2‐containing AMPARs previously reported (Czöndör *et al*, 2012; Penn *et al*, 2017). The recovery was lower for SP^+^ spines with a significantly larger immobile fraction of ∼50% and a time constant τ = 128.3 s, showing greater ability of SP^+^ synapses to stabilize surface diffusing AMPARs in comparison with SP^−^ synapses (Fig 2C and D). Together, these observations suggest that the recruitment of SP at a subset of synapses upon TTX treatment (Fig 1A and B) is associated with the stabilization of surface‐diffusing AMPARs during HSP (Fig 1C–E).

### SP is required for TTX‐induced recruitment of synaptic AMPARs

**Figure 3:**
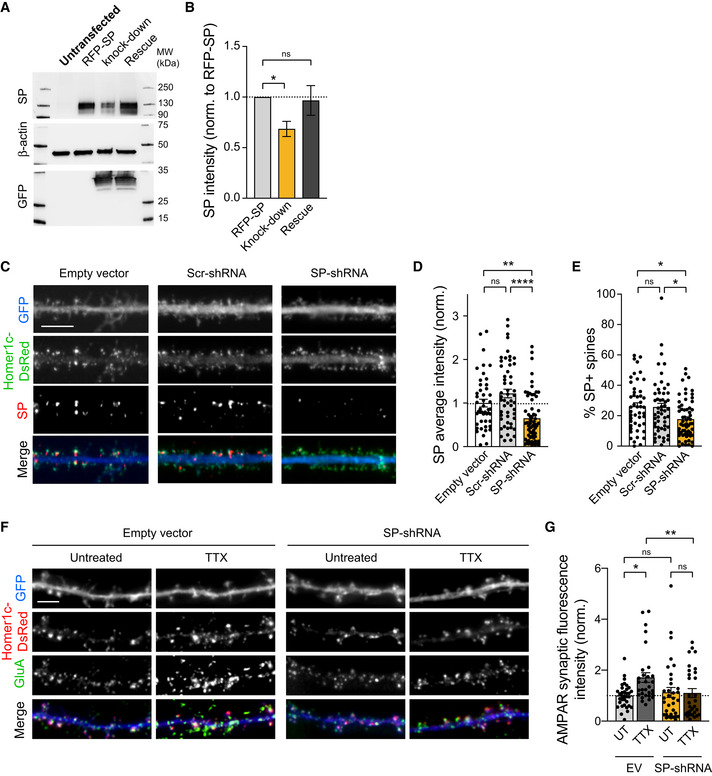
The synaptic recruitment of AMPARs induced by TTX treatment is controlled by SP Immunoblot analysis of SP in lysates from COS‐7 cells which were untransfected, transfected with RFP‐SP + empty vector or co‐transfected with SP‐shRNA‐GFP + RFP‐SP (knock‐down) or shRNA‐resistant RFP‐SP (rescue). β‐actin and GFP signals illustrate equal protein loading and SP‐shRNA expression level, respectively.Quantification of SP expression from six different experiments, normalized to the β‐actin signal. *P < 0.05, ns, not significant, *P* > 0.05 (Kruskal–Wallis test followed by Dunn's multiple comparison test).Homer‐DsRed (green) and immunostained endogenous SP (red) in dendrites from neurons transfected with either empty vector, scramble shRNA or SP‐shRNA with GFP reporter (blue). Scale bar: 10 μm.Average SP intensity for same conditions (EV: *n* = 47; Scr‐shRNA: *n* = 53; SP‐shRNA: *n* = 62, *n* indicates the number of cells, from three cultures; normalized to empty vector condition). **P < 0.01, ****P < 0.0001 (Kruskal–Wallis test followed by Dunn's multiple comparison test).Fraction of SP^+^ synapses for same conditions. (EV: *n* = 47; Scr‐shRNA: *n* = 53; SP‐shRNA: *n* = 62, *n* indicates the number of cells from three cultures). *P < 0.05 (Kruskal–Wallis test followed by Dunn's multiple comparison test).Homer1c‐DsRed (red) and immunostained surface AMPARs (green) in dendrites from neurons transfected with either SP‐shRNA‐GFP or empty vector (EV) with GFP reporter (blue) and treated with TTX or left untreated (UT). Scale bar: 5 μm.AMPAR synaptic fluorescence intensity normalized to untreated empty vector (EV) condition (EV: UT, *n* = 42, TTX, *n* = 33; SP‐shRNA: UT, *n* = 30, TTX, *n* = 31, *n* indicates the number of cells, from three cultures). *P < 0.05, **P < 0.01, ns, not significant, *P* > 0.05 (Kruskal–Wallis test followed by Dunn's multiple comparison test). Data information: Data represent mean ± SEM.

To directly explore the role of endogenous SP in the recruitment of AMPARs during TTX‐induced HSP, we next used a loss‐of‐function approach relying on the expression of a SP‐targeting shRNA along with a GFP reporter (SP‐shRNA‐GFP). Using Western blotting, we found that SP‐shRNA‐GFP downregulates by ∼30% recombinant RFP‐SP expressed in COS‐7 cells, and this effect was rescued when expressing a shRNA‐resistant RFP‐SP construct, thus validating the specificity of the knockdown approach (Fig 3A and B). We then expressed SP‐shRNA‐GFP in DIV8 hippocampal neurons and performed immunostainings at DIV14‐15. The immunofluorescence signal from endogenous SP and the percentage of SP^+^ synapses were decreased by ∼40 and ∼35%, respectively, in neurons expressing SP‐shRNA‐GFP when compared to control neurons transfected with an empty vector or with a scrambled shRNA (Fig 3C–E). These results validated both the specificity of the antibody against SP and the knockdown efficiency. Importantly, knocking down endogenous SP in cultured neurons did not alter the basal synaptic accumulation of AMPARs, but inhibited the increase in synaptic AMPARs induced by TTX treatment (Fig 3F and G). Together with the observation that the presence of SP is predictive of large synapses and correlates with AMPAR stabilization (Figs 1C and EV1A), these results indicate that a sufficient amount of SP is required for synapses to undergo HSP.

### miR‐124 is downregulated in cultured hippocampal neurons upon prolonged TTX treatment

**Figure 4:**
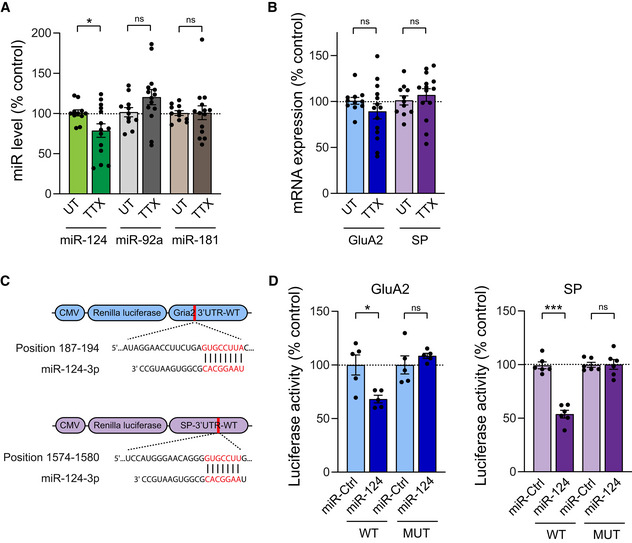
miR‐124 level is selectively downregulated upon TTX treatment and directly inhibits GluA2 /SP translation A, BExpression levels of miR‐124, miR‐92a and miR‐181 (A), and GluA2 and SP mRNAs (B) determined by qRT–PCR in neurons treated with TTX or left untreated (UT). Data are expressed as a percentage of the UT condition (miR‐124: UT, *n* = 11, TTX, *n* = 14; miR‐92a: UT, *n* = 11, TTX, *n* = 14; miR‐181: UT, *n* = 11, TTX, *n* = 14; GluA2 mRNA: UT, *n* = 11, TTX, *n* = 14; SP mRNA: UT, *n* = 11, TTX, *n* = 14, *n* indicates the number of experiments). *P < 0.05, ns, *P* > 0.05 (Mann Whitney test).CSequence alignment showing complementarity between Gria2 or SP 3'UTR and miR‐124 binding seed region (highlighted in red).DLevels of luciferase activity measured from HEK‐293 expressing miR‐Ctrl or miR‐124 together with the Renilla luciferase coding sequence reporter fused to the Gria2 or SP 3'UTR wild‐type (WT) or mutated (MUT) to prevent miR‐124 binding. Data are expressed as a percentage of miR‐Ctrl condition (GluA2‐3'UTR‐WT: miR‐Ctrl, *n* = 5, miR‐124, *n* = 5; GluA2‐3'UTR‐MUT: miR‐Ctrl, *n* = 5, miR‐124, *n* = 5; SP‐3'UTR‐WT, miR‐Ctrl, *n* = 6, miR‐124, *n* = 6; SP‐3'UTR‐MUT: miR‐Ctrl, *n* = 6, miR‐124, *n* = 6, *n* indicates the number of experiments). *P < 0.05, ***P < 0.001, ns, not significant, *P* > 0.05 (Mann–Whitney test). Data information: Data are represented as mean ± SEM.

We next wondered whether the nonuniform upregulation of synaptic AMPAR and SP levels upon TTX treatment could result from the downregulation of miR‐124, which is predicted to bind the 3'UTRs of both GluA2 and SP transcripts and thus might repress their translation in basal conditions (Gascon *et al*, 2014; Ho *et al*, 2014; Elramah *et al*, 2017). To test this hypothesis, we first investigated whether treating cultured hippocampal neurons with TTX could alter miR‐124 expression levels and its targets *GluA2* and *synaptopodin* mRNAs by performing quantitative RT–PCR (qRT–PCR) in DIV15 neurons. The level of miR‐124 was decreased by ∼20% after 48‐h TTX treatment compared with untreated neurons (Fig 4A). In contrast, the amounts of two other miRNAs, miR‐92a and miR‐181, which target GluA1 and GluA2 3'UTRs, respectively, and whose expression levels are altered in response to different plasticity paradigms (Saba *et al*, 2012; Letellier *et al*, 2014; Sambandan *et al*, 2017) were not affected by TTX (Fig 4A). This findings illustrates the selective contribution of miRNAs according to the activity deprivation paradigm (Dubes *et al*, 2019). Importantly, the drop in miR‐124 levels induced by TTX treatment was compatible with the increased expression of SP and GluA2‐containing AMPARs at a subset of spines. However, this was not accompanied by any change in the cellular levels of *GluA2* or *synaptopodin* mRNA (Fig 4B), suggesting that miR124 regulates SP or GluA2 expression in a local manner and/or by inhibiting the translation process.

### miR‐124 inhibits translation of GluA2 and SP through direct interactions with their 3'UTR

To directly assess the ability of miR‐124 to repress GluA2 and SP translation through binding to their respective 3'UTRs, we generated reporter plasmids by fusing the 3'UTR of *GluA2* or *SP* mRNA to the 3′ terminus of a *Renilla* luciferase coding sequence. We then co‐expressed these constructs in HEK‐293 cells together with miR‐124 and measured luciferase activity (Fig 4C and D). miR‐124 expression significantly decreased the luciferase signal by ∼30–45% for both constructs, compared with control miR‐67 (miR‐Ctrl) from *Caenorhabditis elegans* with no reported target in mammals (Fig 4D). Importantly, these effects were prevented when deleting miR‐124 target regions in the 3'UTR of GluA2 and SP (Fig 4D) indicating that miR‐124 can inhibit both GluA2 and SP translation by directly interacting with their 3'UTR.

### miR‐124 overexpression inhibits the synaptic recruitment of endogenous SP and the increase in synaptic strength upon TTX treatment

**Figure 5:**
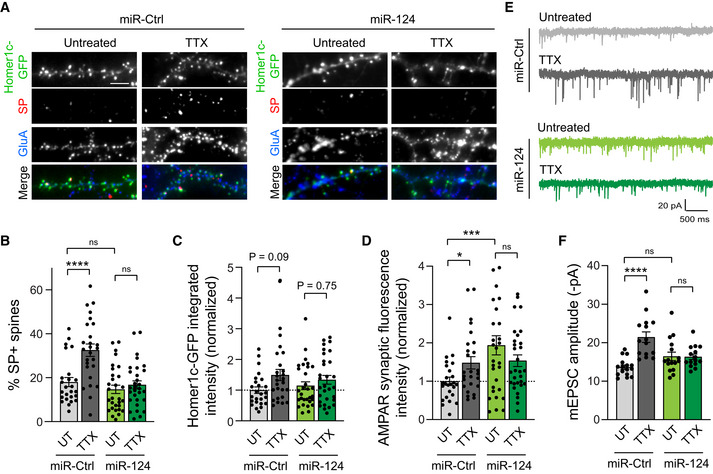
miR‐124 overexpression inhibits TTX‐induced HSP Micrographs showing Homer1c‐GFP (green) and immunostaining for surface AMPARs (blue) and endogenous SP (red) in neurons transfected with miR‐124 or control miR‐67 (miR‐Ctrl), and treated with TTX, or left untreated (UT). Scale bar: 5 μm.Percentage of SP^+^ synapses for same conditions as in (A) (miR‐Ctrl: UT, *n* = 25, TTX, *n* = 26; miR‐124: UT, *n* = 30, TTX, *n* = 30, *n* indicates the number of cells, from three cultures). ****P < 0.0001, ns, not significant, *P* > 0.05 (two‐way ANOVA test followed by Tukey's multiple comparison test).Homer1c‐GFP intensity for same condition as in (A), normalized to untreated miR‐Ctrl (miR‐Ctrl: UT, *n* = 25, TTX, *n* = 26; miR‐124: UT, *n* = 30, TTX, *n* = 30, *n* indicates the number of cells, from three cultures). ns, not significant, *P* > 0.05 (two‐way ANOVA test followed by Tukey's multiple comparison test).AMPAR synaptic fluorescence intensity for same condition as in (A), normalized to untreated miR‐Ctrl (miR‐Ctrl: UT, *n* = 25, TTX, *n* = 26, miR‐124: UT, *n* = 30, TTX, *n* = 30, *n* indicates the number of cells, from three cultures). ***P < 0.001, *P < 0.01, ns, not significant, *P* > 0.05 (two‐way ANOVA test followed by Tukey's multiple comparison test).Representative traces of AMPAR‐mediated miniature currents (mEPSCs) recorded from neurons expressing miR‐124 or miR‐Ctrl in TTX‐treated or untreated neurons.AMPAR‐mEPSC average amplitudes for same condition as in (E) (miR‐Ctrl: UT, *n* = 16, TTX, *n* = 15, miR‐124: UT, *n* = 16, TTX, *n* = 15, *n* indicates the number of cells, from four cultures). ****P < 0.0001, ns, not significant, *P* > 0.05 (Kruskal–Wallis test followed by Dunn's multiple comparison test). Data information: Data are represented as mean ± SEM.

**Figure EV3:**
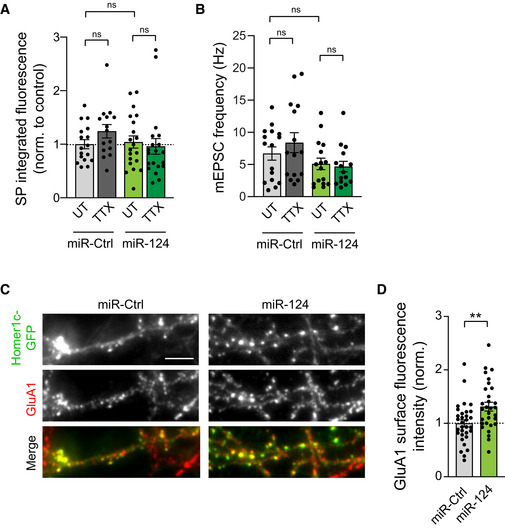
Effect of miR‐124 overexpression on SP expression, mEPSCs frequency and GluA1 synaptic expression SP integrated fluorescence intensity in neurons expressing Homer1c‐GFP and either miR‐124 or control miR‐67 (miR‐Ctrl), and treated with TTX, or left untreated (UT). SP integrated fluorescence intensity was normalized to untreated miR‐Ctrl condition (miR‐Ctrl: UT, *n* = 17, TTX, *n* = 15; miR‐124: UT, *n* = 20, TTX, *n* = 20; *n* indicates the number of cells from two cultures). ns, not significant, *P* > 0.05 (Kruskal–Wallis test).mEPSCs frequency for same conditions as in (A) (miR‐Ctrl: UT, *n* = 16, TTX, *n* = 15, miR‐124: UT, *n* = 16, TTX, *n* = 15; *n* indicates the number of cells from four cultures). ns, not significant, *P* > 0.05 (Kruskal–Wallis test).Micrographs showing neurons expressing Homer1c‐GFP (green) and either miR‐Ctrl or miR‐124, and immunostained for surface GluA1 (red) in untreated neurons. Scale bar: 5 μm.Surface GluA1 fluorescence intensity for same conditions as in (C). GluA1 surface intensity was normalized to miR‐Ctrl condition (miR‐Ctrl: *n* = 32; miR‐124: *n* = 30; *n* indicates the number of cells from two cultures). **P = 0.041 (two‐tailed unpaired *t* test). Data information: Data represents mean ± SEM.

To examine whether miR‐124 downregulation caused by TTX treatment was responsible for HSP, we next asked whether overexpressing miR‐124 in cultured hippocampal neurons could impair the upregulation of endogenous synaptic AMPARs and SP induced by TTX. In basal conditions, miR‐124 overexpression did not significantly affect SP fluorescence intensity at synapses from DIV14 neurons, nor the percentage of SP^+^ synapses in comparison with miR‐Ctrl (Figs 5A and B, and EV3A). These results suggest that endogenous miR‐124 is highly expressed and already strongly represses SP expression in basal conditions. Moreover, Homer1c‐GFP integrated fluorescence intensity and AMPAR‐mEPSC amplitude remained unchanged upon miR‐124 overexpression, while immunostained synaptic AMPARs were increased (Fig 5C–F). This could be explained by the fact that miRNA‐induced suppression of GluA2 expression promotes the assembly and synaptic recruitment of GluA2‐lacking AMPARs at synapses (Hou *et al*, 2015; Silva *et al*, 2019). To test the latter hypothesis, we performed immunostaining of endogenous surface GluA1 using a specific antibody against the N‐terminal domain of GluA1 (Letellier *et al*, 2014) and found higher signal for neurons transfected with miR‐124 in comparison with neurons transfected with miR‐Ctrl (Fig EV3C and D). This observation suggests the molecular replacement of GluA2‐containing AMPARs by GluA1‐containing AMPARs in neurons overexpressing miR‐124.

Importantly, overexpressing miR‐124 blocked the increase in the percentage of SP^+^ synapses, synaptic AMPAR intensity, and average mEPSC amplitudes induced by TTX treatment (Fig 5A–F). These results suggest that the decrease in miR‐124 level upon TTX treatment is required to enable the synaptic recruitment of AMPARs and SP as well as the increase in mEPSC amplitude upon TTX treatment. No significant change in mEPSC frequency was observed in any of the conditions (Fig EV3B), indicating that miR‐124 expression or TTX treatment have no effect on presynaptic function or the number of active synapses.

### The synaptic recruitment of GluA2‐containing AMPARs upon TTX treatment does not require the GluA2‐3'UTR miR‐124 binding region

**Figure 6:**
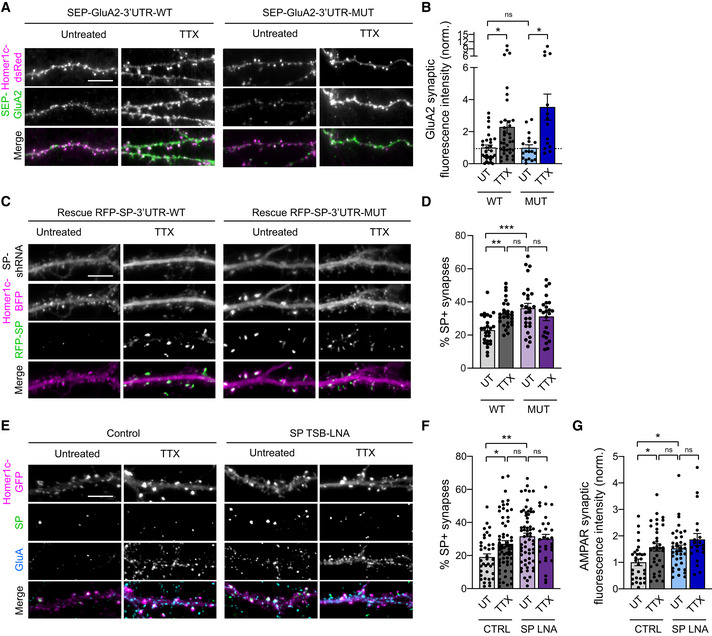
SP but not GluA2 synaptic recruitment upon TTX is controlled by endogenous miR‐124 Micrographs showing dendrites from neurons transfected with Homer1c‐DsRed (magenta) and SEP‐GluA2 constructs containing wild‐type (WT) or mutated (MUT) 3'UTR (green) and treated with TTX for 48 h, or left untreated (UT). Scale bar: 10 μm.SEP‐GluA2 synaptic fluorescence intensity for each condition, normalized to GluA2‐3'UTR‐WT untreated neurons (3'UTR‐WT: UT, *n* = 29, TTX, *n* = 37; 3'UTR‐MUT: UT, *n* = 18, TTX, *n* = 14; *n* indicates the number of cells, from three cultures). *P < 0.05, ns, not significant, *P* > 0.05 (Kruskal‐Wallis test followed by Dunn's multiple comparison test).Micrographs showing dendrites from neurons transfected with Homer1c‐BFP (magenta), GFP‐SP‐shRNA (gray) and a rescue RFP‐SP construct containing wild‐type (WT) or mutated (MUT) 3'UTR (geen) and treated with TTX, or left untreated (UT). Scale bar: 10 μm.Percentage of SP^+^ synapses for each condition (3'UTR‐WT: UT, *n* = 26, TTX, *n* = 28; 3'UTR‐MUT: UT, *n* = 26, TTX, *n* = 24; *n* indicates the number of cells, from three cultures). **P < 0.01, ***P < 0.001, ns, not significant, *P* > 0.05 (two‐way ANOVA test followed by Tukey's multiple comparison test).Micrographs showing Homer1c‐GFP (magenta) and immunostaining for surface AMPARs (cyan) and endogenous SP (green) in neurons transfected with or without 50 nM SP TSB‐LNA and treated with TTX, or left untreated (UT). Scale bar: 10 μm.Percentage of SP^+^ synapses for each condition (Control: UT, *n* = 36, TTX, *n* = 52; SP TSB‐LNA: UT, *n* = 62, TTX, *n* = 29; *n* indicates the number of cells from three cultures). *P < 0.05, **P < 0.01, ns, not significant, *P* > 0.05 (Kruskal–Wallis test followed by Dunn's multiple comparison test).AMPAR synaptic fluorescence intensity for each condition, normalized to control untreated neurons (Control: UT, *n* = 30, TTX, *n* = 36; SP TSB‐LNA: UT, *n* = 37, TTX, *n* = 20; *n* indicates the number of cells from two cultures). *P < 0.05, ns, not significant, *P* > 0.05 (Kruskal–Wallis test followed by Dunn's multiple comparison test). Data information: Data are represented as mean ± SEM.

We next investigated whether miR‐124 binding to the GluA2 3'UTR could control the upregulation of synaptic AMPARs during HSP. We hypothesized that miR‐124 downregulation could lead to the derepression of GluA2 expression upon TTX treatment, thereby contributing to HSP by increasing the total pool of available AMPARs. To test this hypothesis, we designed recombinant SEP‐GluA2 constructs fused to their respective 3'UTR sequences containing or lacking the miR‐124 binding region (‐WT or ‐MUT, respectively). This allowed us to selectively impair the interaction of endogenous miR‐124 with recombinant GluA2 transcripts without compromising the pathways mediated by other miR‐124 targets. Cultured neurons were transfected at DIV8 with SEP‐GluA2‐3'UTR‐WT or SEP‐GluA2‐3'UTR‐MUT plus Homer1c‐DsRed as a postsynaptic marker and subsequently processed at DIV14 for live immunostaining of surface recombinant AMPARs using an anti‐GFP antibody (Fig 6A). In neurons transfected with SEP‐GluA2‐3'UTR‐WT, TTX treatment induced a twofold increase in the synaptic accumulation of SEP‐GluA2 compared with untreated neurons, showing that recombinant AMPARs contributed to HSP similarly to endogenous ones (Fig 6A and B). Surprisingly, however, deleting the miR‐124 interacting region in the GluA2‐3'UTR did not affect the basal surface levels of SEP‐GluA2, nor its ability to get recruited at synapses upon TTX treatment (Fig 6A and B). These results suggest that endogenous miR‐124 does not strongly repress GluA2 expression in basal conditions and make unlikely that GluA2 derepression by miR‐124 contributes to HSP.

### The synaptic recruitment of SP upon TTX treatment requires the SP‐3'UTR miR‐124 binding region

**Figure EV4:**
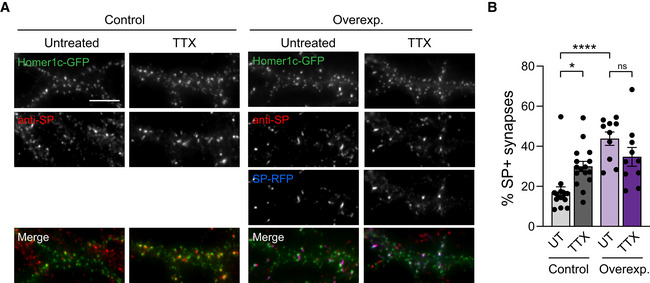
TTX‐induced increase in the percentage of SP^+^ synapses is occluded by SP overexpression Micrographs showing transfected neurons with Homer1c‐GFP alone (control, green) or with SP‐RFP (overexp., blue), and treated with TTX, or left untreated (UT). SP (endogenous and exogenous) was immunostained using anti‐SP antibody (red). Scale bar: 10 μm.Percentage of SP^+^ synapses for each condition (Control: UT, *n* = 16, TTX, *n* = 16, Overexp.: UT, *n* = 10, TTX, *n* = 10; *n* indicates the number of the cells from). *P < 0.05, ****P < 0.0001, ns, not significant, *P* > 0.05 (two‐way ANOVA test followed by Tukey's multiple comparison test). Data information: Data represent mean ± SEM.

Using a similar strategy as for GluA2, we next investigated whether miR‐124 binding to the SP 3'UTR could control the upregulation of SP in neurons during HSP. To this end, we designed recombinant RFP‐SP constructs fused to their respective 3'UTR sequences containing or lacking the miR‐124 binding region (‐WT or ‐MUT, respectively) and expressed them in cultured hippocampal neurons. To limit the effect of overexpressing exogenous RFP‐SP that could occlude the homeostatic response (Fig EV4), we opted for a replacement strategy and co‐expressed SP‐shRNA with GFP reporter along with shRNA‐resistant RFP‐SP‐3'UTR‐WT/MUT constructs and Homer1c‐BFP as a postsynaptic marker. In neurons expressing RFP‐SP‐3'UTR‐WT, ∼23% of synapses were found associated with RFP‐SP clusters and this percentage reached ∼33% after 48‐h TTX treatment, thus reproducing the behavior of endogenous SP in control neurons and validating our replacement strategy (Figs 1A and B, 6C and D, and Fig EV4). In contrast, neurons transfected with RFP‐SP‐3'UTR‐MUT displayed as high as ∼35% of synapses containing RFP‐SP clusters in basal conditions (Fig 6C and D). Importantly, this percentage did not further increase following TTX treatment, indicating that deleting the miR‐124 binding region in the SP 3'UTR occluded the homeostatic response by de‐repressing SP translation.

### Expression of a target site blocker LNA to prevent the interaction between endogenous miR‐124 and endogenous SP occludes HSP

To further test the hypothesis that the regulation of SP expression by miR‐124 is sufficient to promote HSP, we next sought to directly impair the interaction between endogenous miR‐124 and endogenous SP using a target site blocker locked nucleic acid (TSB‐LNA). Because this strategy aims at protecting SP mRNA from miR‐124 rather than directly inhibiting miR‐124, it is expected to spare the other mRNA targets of miR‐124 and therefore to be more specific of the endogenous SP interaction. Similar to our replacement strategy using SP‐3'UTR‐MUT, we found that hippocampal neurons transfected with SP TSB‐LNA together with Homer1c‐GFP as a postsynaptic marker displayed a higher percentage of SP^+^ synapses compared with control neurons (SP TSB‐LNA: ∼31% vs. control: ∼18%; Fig 6E and F). Interestingly, this effect was accompanied by an increase in the fluorescence intensity of immunostained synaptic AMPARs (Fig 6E and G), suggesting that blocking the interaction between endogenous SP mRNA and miR‐124 was sufficient to promote AMPAR synaptic recruitment. Finally, 48‐h TTX treatment did not further increase the percentage of SP^+^ synapses or synaptic AMPAR immunosignal in neurons transfected with SP TSB‐LNA, which was in contrast with the control condition (Fig 6E–G). Therefore, transfecting neurons with SP‐TSB‐LNA occluded the effect of the TTX treatment, showing that derepression of SP by miR‐124 upon activity deprivation is sufficient to mediate HSP.

### Local SP translation is enhanced upon TTX treatment and occurs preferentially in proximity of large synapses

Our results thus far suggest that miR‐124 downregulation upon TTX treatment allows the derepression of SP (but not GluA2) expression at synapses, thereby promoting HSP. Importantly, the discrete distribution of SP at a subset of large synapses and the fact that TTX treatment increased the fraction of those synapses indicate that the homeostatic response is not uniform, possibly involving the local translation of SP. This idea is supported by the fact that both SP mRNA and miR‐124 have been detected in the synapto‐dendritic compartment through *in situ* hyridization or RNA analysis of synaptosomal fraction or micro‐dissected neuropil (Kye *et al*, 2007; Lugli *et al*, 2008; Yamazaki *et al*, 2008; Siegel *et al*, 2009; Cajigas *et al*, 2012; Ho *et al*, 2014; Hafner *et al*, 2019; Konietzny *et al*, 2019). We could notably confirm by FISH the presence of miR‐124 in dendrites from hippocampal neurons at DIV14 (Appendix Fig S3).

**Figure 7:**
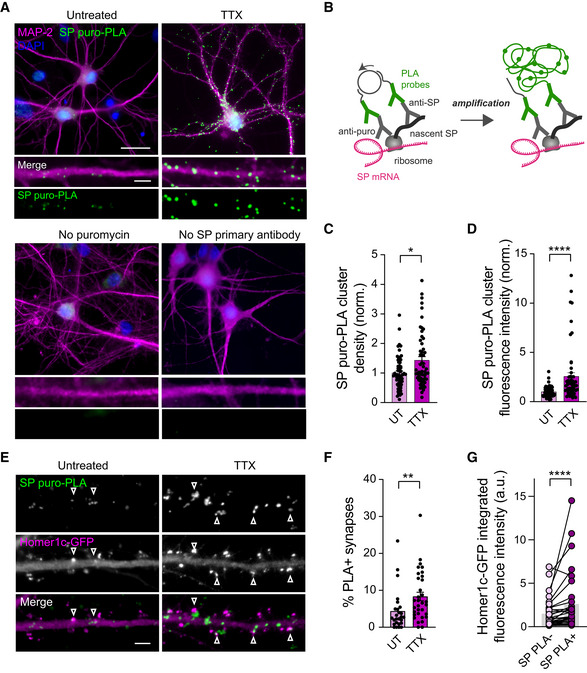
Local SP translation is enhanced upon TTX treatment and occurs preferentially in proximity of large synapses Micrographs showing puro‐PLA staining of newly synthesized SP (green) in neurons immunostained for MAP‐2 (magenta) and DAPI (blue) and treated with TTX for 24 h or left untreated. The images on bottom panels show no staining in the absence of puromycin treatment or when omitting SP primary antibody. Scale bars: 30 μm (large view), 5 μm (insets).Puro PLA labeling strategy showing ligation and amplification steps.Density of SP puro‐PLA puncta, normalized to the untreated condition (UT) (UT: *n* = 62; TTX: *n* = 57, *n* represents the number of cells, from three cultures). *P < 0.05 (Mann–Whitney test).SP puro‐PLA cluster fluorescence intensity normalized to the untreated condition (UT) (UT: *n* = 62; TTX: *n* = 57, *n* indicates the number of cells, from three cultures). ****P < 0.0001 (Mann–Whitney test).Micrographs showing puro‐PLA staining of newly synthesized SP (green) in neurons transfected with Homer1c‐GFP (magenta) and treated with TTX for 24 h or left untreated. Scale bar: 5 μm. Arrowheads indicate puro‐PLA^+^ spines.Percentage of SP puro‐PLA^+^ synapses (UT, *n* = 26, TTX, *n* = 33; *n* indicates the number of cells, from two cultures). **P < 0.01, ns, not significant (Mann–Whitney test).Homer1c‐GFP integrated fluorescence intensity at SP puro‐PLA^−^ vs. SP puro‐PLA^+^ synapses (*n* = 32 cells, from two cultures). ****P < 0.0001, ns, not significant (Wilcoxon matched‐pairs signed rank test). Data information: Data are represented as mean ± SEM.

To test the hypothesis that SP is locally synthesized in dendrites during HSP, we next performed a puromycin proximity ligation assay (PLA) which was previously developed to visualize nascent protein synthesis (tom Dieck *et al*, 2015). Taking advantage of our antibody against SP and following an established protocol (tom Dieck *et al*, 2015), we could directly reveal SP translation sites within individual neurons immunostained for MAP‐2 or transfected with Homer1c‐GFP (Fig 7A and B). We detected SP Puro‐PLA signal puncta in the cell body and along dendrites (Fig 7A) consistent with local SP translation. No Puro‐PLA signal was detected when omitting the SP antibody or puromycin incubation (Fig 7A), validating the specificity of the signal. Interestingly, a fraction of Homer1c‐GFP‐positive synapses (∼5%) was found overlapping with SP puro PLA clusters (PLA^+^ synapses, see Materials and Methods; Fig 7E and F), suggesting that SP can be directly synthesized within spines, possibly reflecting the direct assembly of SP clusters previously reported (Konietzny *et al*, 2019). Incubating neurons with TTX significantly increased both the number and signal intensity of SP puro‐PLA puncta along dendrites (Fig 7A–D), suggesting that the number of SP translation sites and the amount of newly synthesized SP at individual sites were both increased. We also found that the fraction of SP puro‐PLA^+^ synapses was increased by twofold (∼10%) following the TTX treatment (Fig 7E and F). Based on the homer1c‐GFP signal, those synapses were larger in size compared with SP puro‐PLA^−^ synapses (Fig 7G), suggesting a preferential translation of SP at large vs. small synapses.

### Sparse input silencing reveals synapse‐autonomous recruitment of SP and AMPARs in cultured hippocampal neurons

**Figure EV5:**
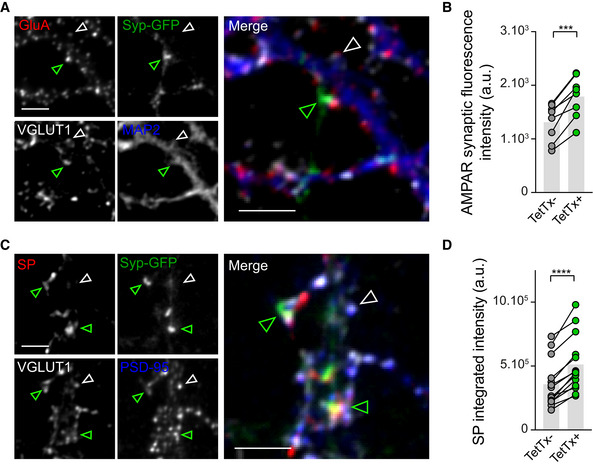
Synapse‐autonomous homeostatic regulation of endogenous AMPARs and SP upon presynaptic silencing in cultured hippocampal neurons Micrographs showing dendrites from cultured hippocampal neurons immunostained for MAP2 (blue), VGLUT1 (gray) and AMPAR (red) and contacted by presynaptic terminals expressing Syp‐GFP + TetTx (green). Arrowheads indicate GFP^+^ (green) and GFP^−^ (white) terminals, immunopositive for VGLUT1. Scale bar: 5 μm.AMPAR synaptic fluorescence intensity of clusters apposed to TetTx^−^ vs. TetTx^+^ terminals (*n* = 8 cells from three cultures). ****P < 0.0001 (Mann Whitney test).Micrographs showing dendrites from cultured hippocampal neurons immunostained for endogenous PSD‐95 (blue), VGLUT1 (gray) and SP (red) and contacted by presynaptic terminals expressing Syp‐GFP + TetTx (green). Arrowheads indicate GFP^+^ (green) and GFP^−^ (white) terminals, immunopositive for VGLUT1. Scale bar: 5 μm.SP integrated fluorescence intensity of clusters apposed to TetTx^−^ vs. TetTx^+^ terminals (*n* = 11 cells from three cultures). ****P < 0.0001 (Mann Whitney test). Data information: Data represent mean ± SEM.

Considering the ability of neurons to synthesize SP in proximity of synapses upon global activity deprivation, we next investigated whether individual synapses could undergo HSP in response to local presynaptic activity blockade and whether this response was synapse‐autonomous. To test this possibility, we opted for a genetic approach where individual synaptic inputs are silenced through the expression of the tetanus toxin light chain (TetTX), which blocks neurotransmitter release through the proteolytic activity of the toxin against the requisite synaptic vesicle SNARE protein VAMP2 (Ehlers *et al*, 2007). We first examined in cultured hippocampal neurons whether the silencing of a subset of synaptic inputs using TetTx expression could induce a local homeostatic upregulation of AMPARs and SP at corresponding postsynapses. To this end, we carried out a sparse transfection of cultured neurons with a DNA construct in which the TetTx coding sequence was inserted downstream of the synaptophysin‐GFP (Syp‐GFP) sequence followed by the internal ribosome entry sequence (IRES) to visualize silenced presynaptic terminals with GFP (Ehlers *et al*, 2007). After 48‐h expression, the cultures were processed for immunostaining of surface endogenous AMPARs or endogenous SP and counterstained for MAP2 or PSD‐95 to visualize dendrites or postsynaptic densities, respectively, and for VGLUT1 to visualize glutamatergic terminals. AMPARs and SP immunosignals in the postsynaptic neuron were higher when clusters were opposed to GFP‐positive boutons from transfected neurons compared with VGLUT1 immunopositive boutons from non‐transfected neurons (Fig EV5A–D). This suggested that SP and AMPARs accumulate more at silenced but not active synapses, thus representing a synapse‐autonomous mechanism for HSP.

### Synapse‐autonomous HSP requires SP‐3'UTR miR‐124 binding region at CA3 recurrent synapses from organotypic hippocampal slices

**Figure 8:**
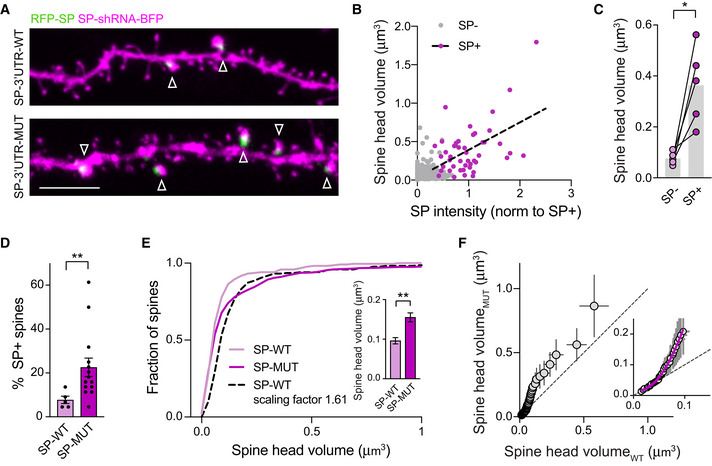
Non‐uniform regulation of spine size by SP‐3'UTR miR‐124 binding region Confocal images showing dendrites from CA3 pyramidal cells transfected with RFP‐SP (green) containing wild‐type (WT) or mutated (MUT) 3'UTR + SP‐shRNA‐BFP (magenta). Arrowheads indicate SP^+^ spines. Scale bar: 10 μm.Graph plot showing spine head volume vs. SP integrated intensity (*n* = 5 cells). Each plot represents a single spine for which SP integrated intensity has been normalized to average SP intensity of SP^+^ spines in the same neuron. Plots for SP^−^ and SP^+^ spines appear in gray and magenta, respectively. The black dotted line represents linear regression (*R* ^2^ = 0.25, *P* = 0.0004).Paired data showing spine head volume for SP^−^ vs. SP^+^ spines. Each pair of plot represents average values for SP^−^ vs. SP^+^ spines for a given neuron. *P = 0.0031 (two‐tailed Student's *t*‐test).Percentage of SP^+^ spines in CA3 pyramidal cells transfected with SP SP‐shRNA‐BFP and rescue RFP‐SP containing wild‐type (WT) vs. mutated (MUT) 3'UTR (SP‐3'UTR‐WT: *n* = 5 cells, from five slices; SP‐3'UTR‐MUT: *n* = 14 cells, from five slices). **P = 0.0029 (Mann–Whitney test).Cumulative probability distributions of spine head volumes for neurons expressing RFP‐SP‐3'UTR‐WT (SP‐WT, light magenta) or RFP‐SP‐3'UTR‐MUT (SP‐MUT, dark magenta). The black dotted curve represents cumulative probability distribution corresponding to RFP‐SP‐3'UTR‐WT condition scaled to RFP‐SP‐3'UTR‐MUT (scale factor = 1.61). ****P < 0.0001 by Kolmogorov–Smirnov test. The inset shows average spine head volume for same conditions (SP‐3'UTR‐WT: *n* = 220 from five cells; SP‐3'UTR‐MUT: *n* = 616, from 14 cells, *n* indicates the number of spines). **P < 0.01 (Mann–Whitney test).Plot showing the rank‐ordered spine head volumes for neurons expressing RFP‐SP‐3'UTR‐WT vs. RFP‐SP‐3'UTR‐MUT. The rank‐order plot was obtained by sorting from smallest to largest spine head volumes in SP‐WT vs. SP‐MUT and plotting them against each other. The extra sum of squares F test indicates that the first values are better fitted with a second‐order polynomial quadratic curve (in magenta, SP‐MUT =  0.02 − 0.42 × SP‐WT + 25.32 × SP‐WT^2^; *R* ^2^ = 0.99; ****P < 0.0001) than with a linear regression (not shown, SP‐MUT = −0.05 + 2.42 × SP‐WT). Data information: Data are represented as mean ± SEM.

To test whether such synapse‐autonomous HSP is also present in preserved neuronal circuits and depends on the interaction between miR‐124 and SP‐3'UTR, we turned to the CA3 recurrent circuit in organotypic hippocampal slices. We first sought to determine how SP distributes across spines from dendrites contacted by recurrent axon collaterals and whether its expression is regulated by miR‐124. To this end, we transfected single CA3 pyramidal cells at DIV21 with RFP‐SP‐3'UTR‐WT or ‐MUT through infusing plasmids by whole‐cell patch‐clamp (Letellier *et al*, 2019) while knocking down endogenous SP with SP‐shRNA‐BFP allowing us to visualize dendritic spine morphology (Fig 8A). In neurons expressing RFP‐SP‐3'UTR‐WT, we found that ∼8% of dendritic spines contained SP‐RFP clusters (Fig 8A and D). Those SP^+^ spines were larger compared with SP− spines, which was in agreement with our results in dissociated cultures and suggested that they contained more AMPARs (Fig 8A–C; Matsuzaki *et al*, 2001; Vlachos *et al*, 2009). Mutating SP‐3'UTR to prevent miR‐124 binding led to a threefold increase in the percentage of SP^+^ spines (Fig 8D). In parallel, we found that neurons expressing SP‐3'UTR‐MUT displayed larger spines compared to neurons expressing SP‐3'UTR‐WT. Interestingly, the difference was greater when comparing the largest spines than when comparing the smallest spines (Fig 8E and F), suggesting a nonuniform regulation of spines that was reminiscent of the nonuniform HSP observed in primary neurons upon TTX treatment (Figs 1, EV1 and EV2). These results demonstrate that miR‐124 exerts a continuous repression on SP translation in a subpopulation of spines.

**Figure 9:**
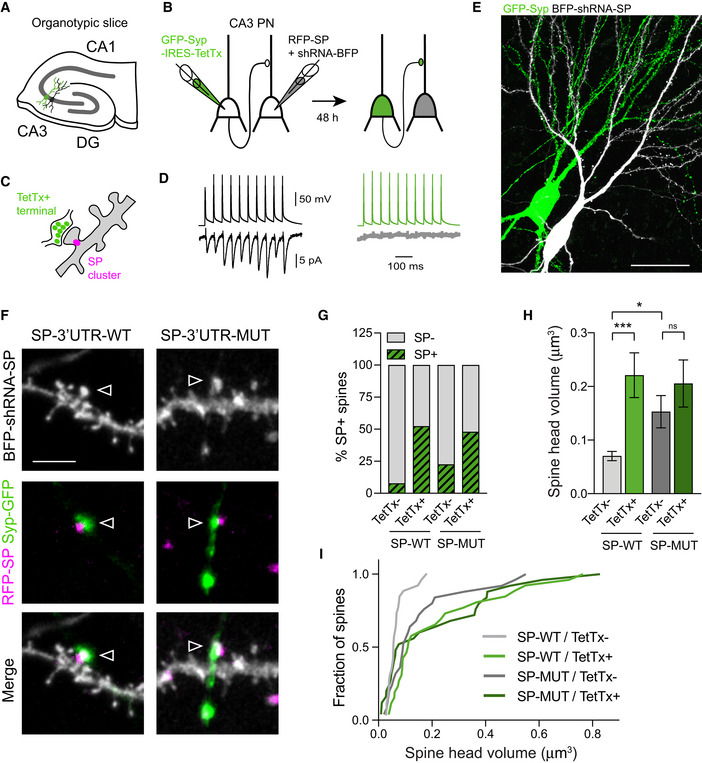
miR‐124‐dependent synapse‐autonomous HSP at CA3 recurrent synapses AExperimental design to silence individual presynaptic inputs in recurrent circuits between CA3 pyramidal cells in organotypic hippocampal slices.B, CWhole‐cell recordings are used to ensure the functional connectivity between two CA3 pyramidal cells while infusing plasmids encoding GFP‐Syp‐IRES‐TetTx and rescue RFP‐SP + BFP‐shRNA‐SP in the pre‐ and postsynaptic cells, respectively (B). Slices were fixed 48 h after transfection and processed for confocal microscopy to analyze spines contacted by GFP^+^ terminals vs. neighboring spines from the same dendritic branch (C).DPair recordings from functionally connected neurons during transfection (left) and 24 h after transfection (right). Top traces (black and green) show train of 10 action potentials elicited in the presynaptic cell. Bottom traces show corresponding evoked postsynaptic currents (black, average from 20 sweeps) during transfection but not 24 h after (gray, average from 20 sweeps).EConfocal image showing a pair of pre and postsynaptic CA3 pyramidal neurons transfected with GFP‐Syp‐IRES‐TetTx (green) and rescue RFP‐SP (not shown) + SP‐shRNA‐BFP (gray), respectively. Scale bar: 40 μm.FHigher magnification of dendritic spines from neurons transfected with rescue RFP‐SP (magenta) containing wild‐type (WT) or mutated (MUT) 3'UTR and BFP‐shRNA‐SP (gray) and contacted by GFP‐Syp + terminals (green). Arrowheads indicate putative synaptic contacts. Scale bar: 5 μm.GPercentage of SP^+^ spines in neurons expressing RFP‐SP‐3'UTR‐WT vs. ‐MUT and depending on the apposition or not to a GFP‐Syp + terminal (SP‐3'UTR‐WT: TetTx^−^, *n* = 220, TetTx^+^, *n* = 26; SP‐3'UTR‐MUT: TetTx^−^, *n* = 616, TetTx^+^, *n* = 25; *n* indicates the number of spines from 5–7 pairs).HSpine head volume in same conditions as in (G) (SP‐3'UTR‐WT: TetTx^−^, *n* = 26, TetTx^+^, *n* = 26 spines from five pairs, four slices; SP‐3'UTR‐MUT: TetTx^−^, *n* = 25, TetTx^+^, *n* = 25; *n* indicates the number of spines from seven pairs, seven slices). ***P < 0.001, *P < 0.05, ns, not significant (Kruskall‐Wallis test followed by Dunn's multiple test).ICumulative probability distributions of spine head volumes for TetTx^−^ and TetTx^+^ spines from neurons expressing RFP‐SP‐3'UTR‐WT (SP‐WT, light gray and light green) or RFP‐SP‐3'UTR‐MUT (SP‐MUT, dark gray and dark green). Data information: Data are represented as mean ± SEM.

We next sought to determine whether miR‐124‐dependent SP expression could be regulated upon local activity deprivation in organotypic slices. To do so, we took advantage of an approach where functionally connected CA3 pyramidal cells at DIV 21 are genetically manipulated through dual whole‐cell patch‐clamp recordings (Fig 9A and B; Letellier *et al*, 2019). Using this strategy, presynaptic terminals from a single input were silenced through the whole‐cell infusion of the GFP‐syp‐IRES‐TetTx plasmid into the presynaptic neuron while endogenous SP was knocked down in the postsynaptic cell through the infusion of SP‐shRNA‐EBFP and replaced by RFP‐SP‐3'UTR‐WT or ‐MUT (Fig 9A–E). Forty‐eight hours after transfecting neuron pairs through whole‐cell patch‐clamp, slices were fixed and processed for confocal microscopy. The occurrence of SP clusters as well as spine size were significantly higher for dendritic spines opposed to GFP‐positive presynaptic terminals compared to neighboring spines from the same dendritic branch (Fig 9F–I). These results indicate that presynaptic silencing promotes the local recruitment of SP as well as spine growth. Importantly, this effect was partially occluded by the deletion of the miR‐124 binding region in the SP 3'UTR, indicating the involvement of the derepression of SP translation by miR‐124 (Fig 9F–I).

## Discussion

**Figure 10:**
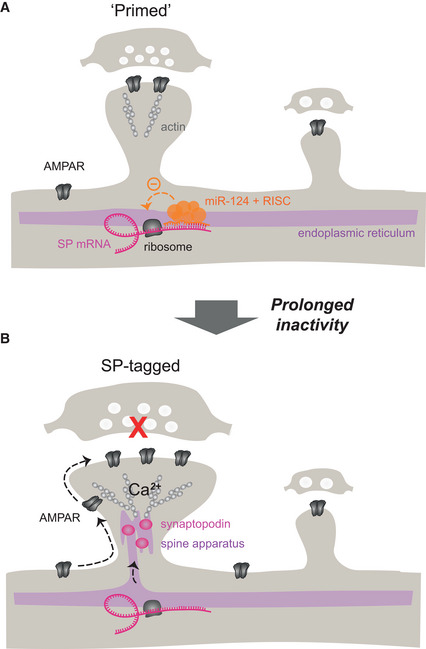
Working model Under basal conditions, a subpopulation of synapses having SP transcripts bound to miR‐124 in their proximity are “primed” for HSP. Upon prolonged inactivity, SP translation by miR‐124 is released at those synapses which get “tagged” by SP. Synapse‐autonomous SP expression promotes capture of surface diffusing AMPARs, spine growth and synaptic strengthening. RISC, RNA‐induced silencing complex.

In this article, we reveal an unsuspected synaptic tagging mechanism for HSP in which the ability of individual synapses to increase their strength following activity deprivation depends on the local expression of SP under the control of miR‐124. Importantly, not only do we demonstrate the differential ability of synapses to undergo HSP but also identify the underlying biochemical and functional determinants. Specifically, our results support a model where SP behaves as a “postsynaptic tag” whose expression at some synapses, but not others, is derepressed by miR‐124 and allows for the capture of surface‐diffusing AMPARs and spine growth to support HSP (Fig 10). Overall, our study shifts the current paradigm that HSP is a process in which all synapses behave uniformly regardless of their activation history, to a more complex view where HSP unexpectedly complies to similar rules as Hebbian plasticity (i.e., input‐specificity and dependence on initial functional and biochemical states), despite opposite induction mechanisms.

### Multiplicative vs. nonuniform HSP

Based on the princeps studies in cultured cortical neurons (O'Brien *et al*, 1998; Turrigiano *et al*, 1998; Turrigiano, 2008), the prevailing view has been that postsynaptic strengths are uniformly scaled by a same factor in response to global change of network activity, regardless of their initial physiological or biochemical state. However, we report here in cultured hippocampal neurons that, in response to 48‐h TTX incubation, relatively large and strong synapses scale with a higher gain compared with small, weaker synapses, resulting in a nonlinear transformation of synaptic weight distribution. Our results are in line with previous findings obtained *in vitro* or *in vivo* showing a “divergent scaling” of synaptic strengths (Thiagarajan *et al*, 2005; Echegoyen *et al*, 2007; Lee *et al*, 2013; Hanes *et al*, 2020) or spine sizes (Hobbiss *et al*, 2018) upon activity deprivation, where synaptic gain increases with initial postsynaptic strength or size. Besides the possible bias introduced by the analysis method used to test the multiplicative nature of HSP (Kim *et al*, 2012; Hanes *et al*, 2020), the discrepancy across studies might result from differences in experimental conditions, including the developmental stage (Goel & Lee, 2007), the duration of the activity perturbation (Hobbiss *et al*, 2018) and the culture conditions (Bardy *et al*, 2015). Interestingly, the fact that the initial synaptic strength determines the homeostatic gain suggests that the ability of a given synapse to undergo future HSP depends on its own activation history, a phenomenon referred to as “metaplasticity” and which was initially thought to be restricted to Hebbian plasticity (Christie & Abraham, 1992; Huang *et al*, 1992). Therefore, while nonuniform HSP still counteracts activity deprivation by making the cell more excitable, it surprisingly promotes runaway dynamics at the single synapse level (the stronger a synapse is, the stronger it will become during HSP) until saturation is reached.

The non‐multiplicative HSP that we describe here, by selectively strengthening the strongest synapses, may serve to restore normal firing rate in an optimized way (Hanes *et al*, 2020). However, by enhancing relative differences between postsynaptic strengths rather than scaling them uniformly, this form of HSP likely interferes with prior Hebbian synaptic changes and is expected to affect the relative capacity of large vs. small synapses to undergo subsequent LTP (Thiagarajan *et al*, 2007). Accordingly, it was recently shown in organotypic slices that TTX‐induced HSP prevents scaled synapses to undergo future LTP by altering short‐term glutamate release dynamics (Soares *et al*, 2017) while reducing the threshold for postsynaptic LTP at small synapses (Hobbiss *et al*, 2018). Together, these results suggest that HSP induced by activity deprivation at the network level alters synaptic strengths distribution and profoundly affects the rules for individual synapses to undergo Hebbian plasticity and to functionally interact together (Lee & Kirkwood, 2019), with possible consequences for synaptic circuit development (Tien & Kerschensteiner, 2018) and the formation of memory engrams (Lee & Kirkwood, 2019). In particular, nonuniform HSP resulting from network‐wide activity alterations could become maladaptive in some pathological contexts such as Alzheimer's disease and epilepsy where it may be achieved at the expense of synaptic input integration and plasticity (Styr & Slutsky, 2018; Li *et al*, 2019; Galanis & Vlachos, 2020; Lignani *et al*, 2020; Letellier & Cingolani, 2021).

### SP as a molecular tag to promote input‐specific synaptic plasticity

We provide a molecular mechanism for the nonuniform and synapse‐autonomous HSP that is induced by activity deprivation. Specifically, we demonstrate that the divergent behavior of synapses facing local or global activity deprivation can be attributed to the nonuniform distribution of SP, an essential component of the smooth ER‐derived organelle called spine apparatus (Deller *et al*, 2003) which associates with a subpopulation of large and strong synapses (Spacek & Harris, 1997; Orth *et al*, 2005). Our immunocytochemistry data suggest that the increased expression of SP at a subset of synapses following activity deprivation with TTX is responsible for the recruitment and stabilization of AMPARs. Our FRAP experiments further indicate that AMPARs stabilization at SP^+^ synapses occurs through the alteration of lateral diffusion, as previously evidenced for mGluR5 (Wang *et al*, 2016a); this may involve calcium release from internal stores (Vlachos *et al*, 2009) and/or actin remodeling events (Wang *et al*, 2016a; Konietzny *et al*, 2019). Whatsoever, the role of SP in AMPARs synaptic stabilization is activity‐dependent as SP knockdown or knockout strongly impairs both HSP and LTP but not average basal synaptic properties (Deller *et al*, 2003; Vlachos *et al*, 2013). In addition, our experiments in primary cultures and organotypic slices indicate that SP cluster formation, AMPARs synaptic accumulation, and spine growth can be stimulated at individual synapses in response to the blockade of presynaptic glutamate release through TetTx expression. The appearance of SP clusters likely reflects the elaboration and the stabilization of the spine apparatus (Perez‐Alvarez *et al*, 2020), that is, together with the presence of polyribosomes, predictive of activity‐dependent spine enlargement (Chirillo *et al*, 2019). In agreement with our results, a recent electron microscopy study performed in the intact hippocampus revealed that mushroom spines but not smaller protrusions (e.g., thin, stubby, or filopodial) exhibit higher volume and PSD size when contacted by TetTx^+^ presynaptic terminals and more likely contained a spine apparatus (Zhu *et al*, 2021). The input‐specificity of SP recruitment upon activity perturbation is also supported by previous reports showing that high‐frequency stimulation in the dentate gyrus enhances SP immunoreactivity selectively in the corresponding stimulated layers (Yamazaki *et al*, 2008; de Solis *et al*, 2017). Such synapse‐specific regulations are in agreement with the nonuniform distribution of SP, which serves as a molecular “tag” enabling individual synapses to “capture” and accumulate AMPARs, a mechanism that is reminiscent of the “synaptic tagging and capture hypothesis” proposed for late‐LTP (Frey & Morris, 1997). While contrasting on the induction protocol (acute hyperactivity vs. chronic silencing), both models rely on protein synthesis and molecular tagging to ensure input‐specificity of synaptic strengthening. The fact that SP enables individual synapses to undergo potentiation in both Hebbian and homeostatic forms of plasticity underscores its fundamental role and raises the possibility that both types of plasticity coexist and/or occlude each other at individual synapses, possibly reflecting a shift in homeostatic setpoint and/or LTP sliding threshold (Lee & Kirkwood, 2019; Li *et al*, 2019; Galanis & Vlachos, 2020).

### Synapse‐autonomous regulation of SP but not GluA2 expression by miR‐124

What are the mechanisms regulating SP recruitment in response to synapse‐specific activity alteration? We demonstrate that the appearance of SP clusters at synapses following chronic activity deprivation depends on the presence of the miR‐124 binding region in the 3'UTR of SP mRNA. Specifically, deleting the miR‐124 interaction region in SP‐3'UTR or impairing the targeting of endogenous SP mRNA by miR‐124 with TSB‐LNA both occluded the increased expression of SP at a subset of synapses that is induced by activity deprivation, revealing that miR‐124 exerts a continuous repression on SP translation in some synapses and that this repression is released when activity is blocked. Together with the fact that (i) this mechanism can be implemented in a synapse‐specific manner, (ii) activity deprivation stimulates SP translation in proximity of large synapses, and (iii) SP mRNA and miR‐124 are both present in the dendritic compartment according to *in situ* hybridization and transcriptomics experiments (Kye *et al*, 2007; Lugli *et al*, 2008; Siegel *et al*, 2009; Cajigas *et al*, 2012; Ho *et al*, 2014; de Solis *et al*, 2017; Hafner *et al*, 2019), these observations strongly suggest that SP synthesis can be locally stimulated through the derepression by miR‐124 to support input‐specific increase in synaptic strength. The role of miR‐124 downregulation in activity‐dependent synaptic potentiation is further evidenced by the fact that miR‐124 overexpression inhibits synaptic strength increase upon TTX treatment (our study) or after eliciting LTP (Yang *et al*, 2012) while miR‐124 neutralization using a sponge promotes AMPAR synaptic recruitment on its own (Hou *et al*, 2015).

In contrast to SP, deleting the miR‐124 interaction region in the GluA2 3'UTR alone was not sufficient to increase the basal expression of AMPARs at synapses, or their recruitment upon TTX treatment. This might be explained by the fact that additional mechanisms are required to stabilize AMPARs at synapses, among which the derepression of SP synthesis by miR‐124, as discussed above. It is also possible that GluA2 basal expression is not strongly repressed by miR‐124 in proximity of synapses; this is suggested by ISH experiments showing low abundance of GluA2 transcripts in the synapto‐dendritic compartment (Ho *et al*, 2014) and by the fact that GluA2 synaptic expression can be repressed when overexpressing miR‐124 cell‐wide (Gascon *et al*, 2014; Ho *et al*, 2014; Hou *et al*, 2015). In one possible model, GluA2 is primarily synthesized at the cell body level and subsequently targeted to activity‐deprived synapses through trafficking mechanisms (Hangen *et al*, 2018), in which SP and the spine apparatus participate. While our study suggests an important role of the miR‐124/SP mRNA complex in proximity of synapses to mediate HSP, the active mechanisms that (i) drive the localization of miR‐124 and/or SP mRNA at specific synapses and (ii) regulate interactions between miR‐124/SP‐mRNA in relation with the translation machinery or RISC remain to be investigated.

Although the implication of miR‐124 downregulation in our HSP paradigm does not seem to rely on GluA2 synthesis, the situation might be different in other activity deprivation paradigms involving different miRNAs. In particular, miR‐186‐5p exerts a continuous repression on GluA2 synthesis in primary hippocampal neurons, that is released upon pharmacological blockade of AMPARs and NMDARs (Silva *et al*, 2019). Interestingly, this paradigm affects neither miR‐124 nor miR‐92a global levels, and rather results in a uniform upscaling of synaptic strengths. Therefore, the control of GluA2 expression by miR‐186‐5p occurs at the cell body level and affects synapses more widely and uniformly than following action potentials blockade with TTX. Altogether, these findings underscore the existence of multiple miRNA‐specific pathways that neurons can use to adjust HSP in time and space depending on the type of activity perturbations (Dubes *et al*, 2019).

### miR‐124, a versatile miRNA controlling various types of synaptic plasticity

Several lines of evidence indicate that miR‐124 can regulate synaptic function through other targets than SP. In particular, it was previously reported that miR‐124 elevation can also drive HSP by directly repressing GluA2 expression, when activity deprivation is induced by inhibiting both action potentials and NMDARs with TTX and AP5, respectively (Hou *et al*, 2015). In this case, the repression of GluA2 synthesis by miR‐124 promotes the synaptic insertion of homomeric GluA1‐containing AMPARs, and may act in conjunction with the derepression of GluA1 synthesis by miR‐92a (Letellier *et al*, 2014). The fact that miR‐124 elevation favors the assembly and insertion of GluA2‐lacking AMPARs is also supported by our experiments where overexpressing miR‐124 induces a synaptic increase in the GluA1 AMPAR subunit, although we found that this effect is not accompanied by an increase in synaptic strength. Therefore, the bidirectional regulation of miR‐124 expression, which likely depends on NMDARs activity and the duration of activity deprivation, may represent a functional switch to produce a selective homeostatic response with respect to the AMPARs subunit composition that confers specific plastic properties to synapses (Gascon *et al*, 2014; Diering & Huganir, 2018). In addition to the role of miR‐124 in HSP through the targeting of SP or GluA2, there is also evidence that miR‐124 negatively regulates synaptic transmission, LTP, and spine density through the targeting of tyrosine‐protein phosphatase non‐receptor type 1 (PTPN1) that regulates GluA2 synaptic insertion (Wang *et al*, 2018), and of the transcription factors Zif268 (Yang *et al*, 2012) and CREB1 (Wang *et al*, 2016b), with possible implications in spatial learning, epilepsy, and Alzheimer's disease. In one possibility, the derepression of these pathways caused by miR‐124 downregulation could contribute to the homeostatic increase in synaptic strengths that we describe here. Together with previous studies, our results thus position miR‐124 as a master regulator of synaptic plasticity, whose expression can be either upregulated or downregulated depending on the physiopathological context and allows for an exquisite control of key synaptic parameters such as spine size, AMPA receptor number, and subunit composition.

## Materials and Methods

### Reagents and Tools table

**Table:** 

Primary antibodies				
WB, Western Blotting; IF, Immunofluorescence; PLA, Proximity Ligation Assay				
Antibody	Species	Supplier	Cat #	Dilution
Synaptopodin	Rabbit	Synaptic Systems	163002	WB 1:1,000 IF 1:600
Beta‐actin	Mouse	Sigma‐Aldrich	A5316	WB 1:5,000
GFP	Mouse	Roche	11867423001	WB 1:5,000
GFP	Mouse	Roche	11814460001	IF live 1:200
GluA2	Mouse	Synaptic Systems	182411	IF live 1:100
GluA1	Rabbit	Agrobio (Choquet)	2144 clone G02141	IF live 1:100
VGLUT1	Guinea Pig	Merck Millipore	AB5905	IF 1:5,000
MAP2	Rabbit	Merck Millipore	AB5622	IF 1:1,000
MAP2	Guinea Pig	Synaptic Systems	188004	IF 1:2,000
PSD‐95	Mouse	Merck Millipore	MA1‐046	IF 1:500
HA	Rat	Roche	11867423001	IF live 1:100
Pan neuronal	Mouse	Merck Millipore	MAB2300	IF 1:500
Puromycin	Mouse	Kerafast	EQ0001	PLA 1:2,500

Secondary antibodies				
Antibody	Conjugation	Supplier	Cat #	Dilution
anti‐rabbit	HRP	Jackson IR	711‐035‐152	WB 1:5,000
anti‐mouse	HRP	Jackson IR	711‐035‐152	WB 1:10,000
anti‐rabbit	Alexa Fluor 350	Thermo Fisher	A11046	IF 1:800
anti‐rabbit	Alexa Fluor 568	Thermo Fisher	A11011	IF 1:800
anti‐rabbit	Alexa Fluor 647	Thermo Fisher	A21244	IF 1:800
anti‐Guinea Pig	Alexa Fluor 568	Thermo Fisher	A11075	IF 1:800
anti‐mouse	Alexa Fluor 488	Thermo Fisher	A11001	IF 1:800
anti‐mouse	Alexa Fluor 568	Thermo Fisher	A11031	IF 1:800
anti‐mouse	Alexa Fluor 647	Thermo Fisher	A21236	IF 1:800
anti‐rat	Alexa Fluor 568	Thermo Fisher	A11077	IF 1:200
anti‐mouse	Alexa Fluor 647	Thermo Fisher	A21236	IF 1:800

DNA constructs			
Backbone	Promoter	Insert	Source
pcDNA3.1	CMV	Homer1c‐GFP	S. Okabe (Tokyo University)
pcDNA3.1	CMV	Homer1c‐DsRed	Mondin *et al* (2011)
pcDNA3.1	CMV	Homer1c‐BFP	Mondin *et al* (2011)
pcDNA3.1	CMV	SEP‐GluA2‐3'UTR‐WT/‐MUT	This study
pGW1	CMV	HA‐GluA1	Gift from D. Choquet (IINS, Bordeaux)
pGW1	CMV	HA‐GluA2	Gift from D. Choquet (IINS, Bordeaux)
pEGFP	CMV	RFP‐SP	Gift from A. Triller (ENS, PARIS)
pEGFP	CMV	RFP‐SP‐3'UTR‐WT/‐MUT	This study
pEGFP	CMV	shRNA resistant RFP‐SP‐3'UTR‐WT/‐MUT	This study
pIRES‐EGFP	CMV	EGFP‐synaptophysin:IRES:TetTx	Gift from D. Choquet/D. Perrais (IINS, Bordeaux)
pCAG‐miR30	CAG	SP‐shRNA with GFP reporter	This study
pCAG‐miR30	CAG	SP‐shRNA with BFP reporter	This study
pCAG‐miR30	CAG	Scrambled shRNA with GFP reporter	Gift from D. Choquet (IINS, Bordeaux)
phRL‐CMV	CMV	Luciferase	Favereaux *et al* (2011)
pcDNA3.1	U6	miR‐124	This study

Other products		
Product	Reference	Supplier
miRCURY LNA miRNA Target Site Blocker	339199	Qiagen
miRCURY LNA miRNA Detection Probe	339111	Qiagen
TSATM‐Plus Fluorescein System	NEL741001KT	PerkinElmer
Duolink	DUO92008	Merck
DAPI Fluoromount‐G	0100‐20	SouthernBiotech
Bicuculline	0130	Tocris
TTX	1069/1	Tocris
NASPM	2766/10	Tocris

### Methods and Protocols

#### DNA plasmids

The pcDNA Homer1c‐GFP was gift from S. Okabe (Tokyo University, Japan). The pcDNA Homer1c‐DsRed and SEP‐GluA2 3'UTR plasmids were already described Mondin *et al* (2011). To generate SEP‐GluA2‐3'UTR‐MUT, we inserted the mutated sequence (synthetized by Eurofins) at the HpaI/AflII sites in place of the WT sequence. HA‐GluA1 and HA‐GluA2 plasmids were gift from D. Choquet (Interdisciplinary Institute for Neuroscience, Bordeaux). RFP Synaptopodin was a gift from A. Triller (École Normale Supérieure, Institut de Biologie de l'ENS, Paris). RFP‐SP‐3'UTR‐WT/‐MUT were generated by inserting the partial WT or mutated 3'UTR sequence of SP (GTCTCCATGGGAACAGGGGTGCCTTGTCAGTG or GTCTCCATGGGAACAGGGCACGGAAGTCAGTG, respectively) at ApaI/MluI sites in the RFP‐SP plasmid. The corresponding rescue forms Rescue RFP‐SP‐3'UTR‐WT/‐MUT were generated by inserting the sequence resistant to the sh (synthetized by Eurofins) at the XbaI/BamHI sites. EGFP‐synaptophysin:IRES:TetTx construct was a gift from D. Choquet/D. Perrais (Interdisciplinary Institute for Neuroscience, Bordeaux). The target sequence of the shRNA against SP (SP‐shRNA with GFP reporter) was designed with BLOCK‐it RNAi designer (https://rnaidesigner.thermofisher.com/rnaiexpress/), the selected sequence (5'‐GGTGTATAGTGAAGTACATCT‐3′) was inserted in a miR‐30 context (pCAG‐miR‐30, from T. Matsuda). The SP‐shRNA BFP was created by replacing the GFP sequence by BFP2 sequence at the BspeI/BspeI sites. Luciferase constructs were obtained by PCR amplifying the 3'UTR regions from cDNA extracts and cloning them into a modified phRL‐CMV vector (Promega), as previously described (Favereaux *et al*, 2011). For miRNA overexpression, a genomic sequence spanning 150 bp upstream and downstream of the miRNA sequence was PCR‐amplified and cloned into a modified pcDNA3.1 with a U6 promoter (Invitrogen).

#### Target site blocker LNA design

To specifically inhibit the interaction between miR‐124 and SP mRNA, we used a custom‐designed miRCURY LNA miRNA Target Site Blocker (Qiagen). This oligonucleotide contains 5′ and 3′ modifications, a phosphorothioate backbone chemistry and Locked Nucleic acid base chemistry. The nucleotide sequence is designed to overlap the seed region for miR‐124 in the 3'UTR of SP mRNA at positions 1,574–1,580. The sequence of the Target Site blocker is 5'‐GAGCTCACTGACAAGGCACC‐3′.

#### Primary cultures and transfection

Primary rat hippocampal neurons were prepared from hippocampi of E18 Sprague–Dawley embryos (Janvier Labs, Saint Berthevin, France). Hippocampi were dissected out and processed for enzymatic dissociation for 15 min at 37°C in 0.05% trypsin–EDTA solution (Gibco) buffered with HEPES (Gibco) and containing 1% penicillin–streptomycin (100 mg ml^−1^, Gibco). After washes, hippocampi were triturated with a serological pipette and around 450 k cells were plated on glass coverslips (18 mm diameter, Marienfeld, 117580) precoated with Poly‐L‐lysine hydrobromide (1 mg ml^−1^, Sigma‐Aldrich). Cells were placed into culture dishes containing Neurobasal™ medium (Gibco) supplemented with NeuroCult™ SM1 neuronal supplement (STEMCELL), L‐glutamine (2 mM, PAA) and 3% horse serum (Invitrogen) at 37°C in 5% CO_2_. After 2–3 days *in vitro* (DIV), the culture medium was replaced with Neurobasal™ medium without horse serum and from DIV5‐6 and every other 3–4 days, half of the medium was replaced by the same volume of BrainPhys™ medium (STEMCELL) supplemented with NeuroCult™ SM1 neuronal supplement (STEMCELL). Glial cells proliferation was inhibited by adding Ara‐C (cytosine β‐D‐arabinofuranoside hydrochloride, 2.5 μM, Sigma‐Aldrich) at DIV6‐8. To induce HSP, half of the neuronal cultures were treated at DIV13 with 2 μM TTX (Tocris) for 48 h.

To test the effect of the knockdown of SP on AMPAR expression, neurons were co‐transfected after 8 DIV with Homer1c‐DsRed and either shRNA SP with GFP reporter or empty vector (pCDNA3‐GFP) at a ratio of 1:4 and processed for immunocytochemistry at DIV14‐16. For experiments using recombinant SEP‐GluA2‐containing AMPARs, neurons were co‐transfected after DIV8 with SEP‐GluA2 3'UTR‐WT with SP‐RFP at a ratio 1:1 and processed at 10 DIV. For miR‐124 overexpression experiments, neurons were co‐transfected after 10 DIV with Homer1c‐GFP and either miR‐124, miR‐67 (miR control) or empty vector at a ratio of 1:3 using a lipofection protocol (Effecten, Qiagen) and processed at DIV15. To selectively prevent the interaction between miR‐124 and SP‐3'UTR, the rescue RFP‐SP‐3'UTR‐WT/MUT constructs were co‐expressed in cultured hippocampal neurons at DIV8 together with Homer1c‐BFP and SP‐shRNA with GFP reporter before being processed at DIV14. A similar strategy was used to prevent the interaction between miR‐124 and GluA2‐3'UTR: dissociated hippocampal neurons were co‐transfected at 8 DIV with Homer1c‐DsRed and either SEP‐GluA2 3'UTR‐WT or SEP‐GluA2 3'UTR‐MUT at a ratio 1:3 and processed at DIV10. To impair endogenous SP mRNA targeting by endogenous miR‐124, cultured hippocampal neurons were transfected at DIV8 with 50 nM SP TSB‐LNA together with Homer1c‐GFP and processed at DIV14. To inhibit glutamate synaptic release at individual inputs, neurons were transfected after DIV9‐10 with EGFP‐Synaptophysin:IRES:TetTx and processed at DIV13‐14. To test the specificity of antibody against AMPA receptors, neurons were co‐transfected at DIV9 with Homer1c‐GFP and either HA‐GluA1 or HA‐GluA2 at a ratio 1:3 and processed at DIV10.

#### Organotypic slices and whole‐cell patch‐clamp transfection

Organotypic hippocampal slice cultures were prepared from wild‐type mice (C57Bl6/J strain) as described Stoppini *et al* (1991). All animal experiments complied with all relevant ethical regulations. Animals were raised in our animal facility and they were handled and euthanized according to European ethical rules. Briefly, animals at postnatal day 5–8 were quickly decapitated and brains placed in ice‐cold Gey's balanced salt solution under sterile conditions. Hippocampi were dissected out and coronal slices (350 μm) were cut using a tissue chopper (McIlwain) and incubated at 35°C with horse serum‐containing medium on Millicell culture inserts (CM, Millipore). The medium was replaced every 2–3 days. After 21 DIV, slices were transferred to an artificial cerebrospinal fluid (aCSF) containing (in mM): 130 NaCl, 2.5 KCl, 2.2 CaCl_2_, 1.5 MgCl_2_, 10 D‐glucose, 10 Hepes (pH 7.35, osmolarity adjusted to 300 mOsm). Pairs of CA3 pyramidal cells were processed for whole‐cell patch‐clamp recordings to assess functional connectivity while infusing DNA constructs as previously described Letellier *et al* (2019). Recording pipettes were filled with a solution containing (in mM): 130 K‐gluconate, 10 HEPES, 7 KCl, 0.05 EGTA, 2 Na2ATP, 2 MgATP, and 0.5 NaGTP (pH 7.30, osmolarity adjusted to 290 mOsm). During the recording, the presynaptic cell was infused with a plasmid encoding TetTx downstream of the synaptophysin‐GFP and IRES sequences (100 ng μl^−1^ in the recording solution) to label the silenced presynaptic terminals with GFP. Meanwhile, the postsynaptic cell was infused with plasmids encoding (i) shRNA‐SP with a EBFP reporter (150 ng μl^−1^) allowing to knockdown endogenous SP while subsequently visualizing the cell morphology and (ii) shRNA‐resistant SP‐RFP‐3'UTR‐WT or ‐MUT (150 ng μl^−1^) to replace endogenous SP with a recombinant transcript containing or lacking the miR‐124 binding region, respectively. After the recordings, the patch pipettes were gently retracted to facilitate membrane resealing. The slices were placed back in the incubator for 2 more days before being processed for confocal imaging.

#### miRNA target prediction

Rat GluA2 mRNA sequence (encoded by the Gria2 gene) and SP mRNA sequence (encoded by the SYNPO gene) were PCR amplified and cloned from a brain cDNA library. MiRNA target prediction for the Gria2 and the SYNPO genes were performed with Targetscan algorithm (Agarwal *et al*, 2015).

#### Total RNA isolation and qRT–PCR

For miRNA and mRNA quantifications, cultured neurons at 15 DIV were left untreated or treated for 48 h with 2 μM TTX and then processed for total RNA extraction using the kit Direct‐zol™ RNA MicroPrep (Zymo Research) according to the manufacturer's instructions. qRT–PCR was performed using miScript PCR System kit (Qiagen). Reverse transcription was achieved on 1 μg RNA with specific oligo‐dT primers to generate cDNA library using this program: 60 min at 37°C followed by 5 min at 95°C and cooled down to 4°C. Quantitative PCR was carried out on a LightCycler LC480 (Roche) with primer pairs designed to span exon boundaries and to generate amplicons of ∼100 bp. Primer sets for snRNA U6, GAPDH, Gria2, Synpo, miR‐124, miR‐92a, and miR‐181a were tested by qRT–PCR and gel electrophoresis for the absence of primer‐dimer artifacts and multiple products. Triplicate qRT–PCR reactions were done twice for each sample, using transcript‐specific primers (600 nM) and cDNA (5 ng) in a final volume of 10 μl and using this program: 15 min at 95°C and 35 cycles of 15 s at 95°C, 30 s at 55°C then 30 s at 70°C. The Ct value of each gene was normalized against that of snRNA U6 and GAPDH. The relative level of expression was calculated using the comparative (2^−∆∆Ct^) method (Schmittgen & Livak, 2008).

**Table:** 

Targets	Primer sequences
*miR‐124*	Forward: 5' AGGCACGCGGTGAATGCC 3'
*miR‐92a*	Forward: 5' TATTGCACTTGTCCCGGCCTG 3'
*miR‐181*	Forward: 5' AACATTCAACGCTGTCGGTGAGT 3'
Gria2	Forward: 5' CATTTGTCATCCAGATGCGA 3' Reverse: 5' GTTGATAAGCCTCTGTCACTG 3'
Synpo	Forward: 5' ATGGACGTAGCCAGGT 3' Reverse: 5' GGCCTCCTTCAGATCCT 3'
snRNA U6	Forward: 5' GGAACGATACAGAGAAGATTAGC 3' Reverse: 5' AAATATGGAACGCTTCACGA 3'
GAPDH	Forward: 5' GGCCTTCCGTGTTCCTACCC 3' Reverse: 5' CTCCAGGCGGCATGTCA 3'

#### HEK‐293 cell culture and luciferase assay

HEK‐293 cells (supplied by ECACC 85120602) were used to perform luciferase reporter assay. Cells were plated at 400 k in a 25 cm^2^ flask (Dutcher) and cultured in Dulbecco's modified Eagle's medium supplemented with glutamax (3.87 mM), D‐glucose (25 mM), penicillin (100 UI ml^−1^), and streptomycin (100 mg·ml^−1^, Gibco). Cell cultures were maintained at 37°C in a humidified incubator with 5% CO_2_. For luciferase reporter assay, HEK‐293 cells were transfected with pmirGLO Dual‐Luciferase miRNA Target Expression Vector (1 μg, Promega) containing a control reporter gene Renilla luciferase (hRluc) and either the 3'UTR of Gria2 or Synpo fused to the reporter gene Firefly luciferase (luc2). After 24 h, luciferase activity was measured using Dual‐Luciferase Reporter Assay System kit (Promega) then normalized by the activity level of Renilla luciferase (hRluc).

#### COS‐7 cell culture and Western blotting

For Western blotting, COS‐7 cells (supplied by ECACC 87021302) were plated at 120 k cells per well in a 6‐well plates (culture surface: 9.6 cm^2^ FALCON^®^, Dutscher) and cultured in Dulbecco's modified Eagle's medium supplemented with 10% SVF (Eurobio), 1% sodium pyruvate (Sigma‐Aldrich), 1% glutamax (Gibco) and 1% penicillin–streptomycin (100 UI ml^−1^ and 100 mg ml^−1^, Gibco, Invitrogen), and maintained at 37°C in a humidified incubator with 5% CO_2_. After 12–24 h, cells were transfected with SP‐RFP and either shRNA SP‐GFP or empty vector (pCDNA3) using a lipofection method (X‐tremeGENETM HP DNA Transfection Reagent, Roche) at a ratio 1:3. After 36–48 h, cells were rinsed twice in ice‐cold PBS (ET330, Euromedex) and then scraped into 85 μl of RIPA buffer (50 mM Tris–HCl pH 7.5, 1 mM EDTA, 150 mM NaCl, 1% Triton‐X100) containing protease inhibitor cocktail (Millipore). Homogenates were kept for 30 min on ice and then centrifuged at 8,000 × *g* for 15 min at 4°C to remove cell debris. The supernatant was recovered and the protein concentration was estimated using the Direct Detect^®^ Infrared Spectrophotometer (Millipore). Twenty microgram proteins were loaded on 4–15% Mini‐PROTEAN^®^ TGX Precast Protein Gels Stain Free™ (Bio‐Rad^®^) for separation (200 V, 400 mA, 40 min) and were subsequently transferred to nitrocellulose membranes for semi‐dry immunoblotting (25 V, 1.3 A, 7 min, Bio‐Rad^®^). Membranes were dried 5 min at 37°C and then incubated with 5% nonfat dried milk in Tris‐buffered salineTween‐20^®^ (TBST containing 28 mM Tris, 137 mM NaCl, 0.05% Tween‐20^®^, pH 7.4) for 45 min at room temperature. Membranes were rinsed in TBST and cut to be incubated for 1 h at room temperature in 0.5% nonfat dried milk in TBST containing the appropriate primary antibody anti‐SP (rabbit polyclonal, Synaptic Systems, 163002, 1:1,000), anti‐Beta‐actin (mouse monoclonal, Sigma‐Aldrich, A5316, 1:5,000) and anti‐GFP (mouse polyclonal, Roche, 11867423001, 1:5,000). After washing three times with TBST buffer, blots were incubated with horseradish peroxidase (HRP)‐conjugated donkey anti‐rabbit secondary antibody (Jackson Immunoresearch, 711‐035‐152, 1:5,000) or HRP‐conjugated donkey anti‐mouse secondary antibody (Jackson Immunoresearch, 711‐035‐150, 1:10,000) accordingly, in 0.5% non‐fat dried milk in TBST for 1 h at room temperature. Target proteins were detected by chemiluminescence with Clarity™ (Beta‐actin and GFP) and Clarity Max™ (SP) Western ECL Substrate kit (Bio‐Rad^®^) on the Odyssey FC system (LI‐COR). Average intensity values were calculated using Image Studio 5.2 software (LI‐COR^®^). The intensity of SP signal was normalized by the intensity of beta‐actin signal of the sample.

#### Immunocytochemistry

For surface staining of AMPAR subunits, cultured hippocampal neurons were incubated live for 10 min at 37°C with a mouse monoclonal antibody raised against the N‐terminal domain of GluA2 that also recognizes GluA1 and GluA3 (Synaptic Systems, 182411 Clone 248B7) and diluted to 1:100 in the culture medium. For some experiments, neurons were incubated with a rabbit polyclonal antibody raised against the N‐terminal domain of GluA1 and diluted to 1:100 in the culture medium. This antibody was a gift from D. Choquet (IINS; Bordeaux) and has been customized by Agrobio (Agrobio, 2144 clone G02141) using the immunogene RTSDSRDHTRVDWKRC within the extracellular region of the GluA1 subunit (Nair *et al*, 2013). Cells were subsequently fixed in paraformaldehyde (PFA) 4% with 4% sucrose for 10 min and permeabilized with 0.1% Triton X100 (Sigma‐Aldrich) for 5 min. Endogenous SP, VGLUT1, MAP2 and/or PSD‐95 were immunostained using primary antibodies diluted in PBS‐BSA 1% for 30 min (SP: rabbit polyclonal, Synaptic System, 163002, 1:600; VGLUT1: guinea pig polyclonal, Merck Millipore, AB5905, 1:5,000; MAP2: rabbit polyclonal, Merck Millipore, AB5622, 1:1,000; PSD‐95: mouse monoclonal, Merck Millipore, MA1‐046, clone 7E3‐1B8, 1:500). After washes in PBS, neurons were incubated with PBS‐BSA 1% (Sigma‐Aldrich) for 30 min at room temperature and then stained with a secondary antibody conjugated to Alexa fluorophore diluted in PBS‐BSA 1% at 1:800 for 30 min: anti‐rabbit conjugated to either Alexa 350 (A11046, Thermo Fisher), Alexa 568 (A11011, Thermo Fisher) or Alexa 647 (A21244, Thermo Fisher); anti‐guinea pig conjugated to Alexa 568 (A11075, Thermo Fisher); anti‐mouse conjugated to either Alexa 488 (A11001, Thermo Fisher,), Alexa 568 (A11031, Thermo Fisher) or Alexa647 (A21236, Thermo Fisher). For surface staining of HA‐GluA1/GluA2 or SEP‐ GluA2, neurons were incubated live for 10 min at 37°C with anti‐HA rat monoclonal antibody (1:100, 11867423001, Roche) or anti‐GFP mouse monoclonal antibody (1:200, 11814460001, clones 7.1, 13.1, Roche), respectively. Cells were fixed and stained with anti‐rat or anti‐mouse secondary antibody conjugated to Alexa 568 (1:200, A11077, Thermo Fisher) or Alexa 647 (1:800, A21236, Thermo Fisher), respectively.

#### *In situ* hybridization

For *in situ* hybridization of miR‐124, double‐digoxigenin locked nucleic acid probe (RNO‐MIR‐124‐3P: CATTCACCGCGTGCCTTA, Tm: 84°C, 339111 YD00614870‐BGC, miRCURY LNA™ miRNA Detection Probe, Qiagen) was used at a final concentration of 30 nM. Hybridization was performed according to the manufacturer's instructions. Digoxigenin was revealed with a tyramide‐based method (1:50, TSATM ‐Plus Fluorescein System, NEL741001KT, PerkinElmer). Pan neuronal staining was also processed (monoclonal mouse antibodies, 1:500, Neuro‐ChromTM Pan Neuronal Marker Millipore MAB 2300) and revealed with a goat anti‐mouse antibody conjugated to Alexa 568 (1:500, A11031, Thermo Fisher). Nuclei were stained with DAPI (DAPI Fluoromount‐G^®^, SouthernBiotech).

#### Puromycin proximity ligation assay

Live cultured neurons were first incubated with 5 μM puromycin for 10 min in culture medium at 37°C in a humidified atmosphere with 5% CO_2_. The incubation was stopped by two fast washes in prewarmed PBS and cells were fixed for 10 min in PFA‐sucrose at room temperature. After fixation, cells were washed, permeabilized with 0.1% Triton X100 (Sigma‐Aldrich) for 5 min and treated for proximity ligation assay according to the manufacturer's recommendations with minor modifications (Merck). Labeling of newly synthesized SP was performed using mouse anti‐puromycin antibody (EQ0001, Kerafast, 1:2,500) in combination with rabbit anti‐SP antibody (163002, Synaptic System, 1:600) and detection was achieved using Duolink reagents (DUO92008, Merck). Cells were first blocked in PBS‐BSA 1% for 30 min at room temperature and incubated with anti‐puromycin and anti‐SP antibodies for 1 h at room temperature. After washing in wash buffer A, cells were incubated with plus and minus PLA probes (1:5 in dilution buffer) in a humidified chamber at 37°C for 1 h, washed again with wash buffer A, and subsequently treated with the ligation reaction containing the circularization oligonucleotides and ligase in a humidified chamber at 37°C for 30 min. After washes with wash buffer A, amplification and label probe binding was performed with the amplification reaction mixture containing the polymerase and the fluorophore‐labeled detection oligonucleotides in a humidified chamber at 37°C for 100 min. The amplification reaction was stopped by three washes in wash buffer B, followed by washes in PBS. Cells were postfixed for 10 min in PFA‐sucrose at room temperature, washed with PBS and further processed for MAP2 immunostaining using guinea pig anti‐MAP2 (188004, Synaptic Systems, 1:2,000) as described (tom Dieck *et al*, 2015).

#### Epifluorescence microscopy and image analysis

Fluorescence imaging for immunocytochemistry and *in situ* hybridization on primary neuronal cultures was performed using an inverted microscope (Nikon Ti‐E‐Eclipse) equipped with a CMOS Prime 95B Scientific camera (Photometrics), an apochromatic (APO) ×60/1.49 numerical aperture (NA) oil objective and filter sets allowing to image Alexa 350/DAPI (Excitation: FF01‐379/34; Dichroic: FF‐409Di03; Emission: FF‐440/40; semROCK), GFP/Alexa 488 (Excitation: FF01‐472/30; Dichroic: FF‐495Di02; Emission: FF01‐520/35; SemROCK), RFP/Alexa 568 (Excitation: FF01‐543/22; Dichroic: FF‐562Di02; Emission: FF01‐593/40; SemROCK) and Alexa 647 (Excitation: FF02‐628/40; Dichroic: FF‐660Di02; Emission: FF01‐692/40; SemROCK).

Image analysis was performed using custom routines in Metamorph^®^ 7.8 software (Molecular Devices, Sunnyvale, USA). An intensity threshold was first applied on Homer1c‐GFP/‐dsRed/‐BFP signal to define the neurite outline. Within the neurite outline, synaptic regions of interest (ROIs) were defined (regardless of their localization in spine vs. dendritic shaft) through an unbiased binary segmentation of Homer1c‐GFP/dsRed/‐BFP clusters based on wavelet decomposition of the signal (Multidimensional Image Analysis Software ran by Metamorph). The area and average intensity for both Homer1c‐GFP and AMPAR signal were measured for each synaptic ROI. To quantify the percentage of SP^+^ synapses, synaptic ROIs were transferred to the SP image and an intensity threshold of the SP signal was applied. We considered synaptic ROIs as “SP^+^” when ≥ 20% of their pixels were covered by thresholded SP signal. No discrimination was made between shaft and spine synapses.

For experiments using TetTx expression in primary hippocampal cultures, VGLUT1 and MAP2 immunosignals were used to define glutamatergic presynaptic terminals and dendritic regions, respectively. A 14 × 14 pixels region surrounding VGLUT1 puncta was then defined. Within this region, integrated intensity for either AMPAR or SP signal was measured using Metamorph^®^ 7.8 software (Molecular Devices). Three to four dendritic areas per neuron were analyzed.

#### FRAP experiment and analysis

FRAP experiments were performed on SEP‐GluA2 signal for SP^+^ and SP^−^ spines using an inverted microscope (Nikon Ti‐E‐Eclipse) equipped with an EMCCD camera (Evolve 512, Photometrics) driven by Metamorph^®^ software 7.10 (Molecular Devices), an APO ×100/1.49 NA oil objective and filter sets to image SEP (Excitation: FF01‐472/30; Dichroic: FF‐495Di02; Emission: FF01‐520/35) and RFP (Excitation: FF01‐543/22; Dichroic: FF‐562Di03; Emission: FF01‐593/40). A laser bench comprising 491 and 561 lasers (100 mW each, Roper Scientific) was used to image or bleach SEP signal through a secondary optical fiber output connected to a device containing galvanometric scanning mirrors (ILAS, Roper Instrument). This device was driven by Metamorph^®^ software and allowed for precise spatial and temporal control of photobleaching in a user‐defined targeted area. Switching between the two fibers for alternating between imaging and bleaching was performed in the millisecond range using a mirror. After acquiring a 30‐s baseline every 5 s, a rapid selective photobleaching of 6 to 10 spines (10 × 10 pixels ROIs covering the entire spine) was achieved using the 491 nm laser at higher level power (3 mW at the front of the objective) in less than 200 ms. Spines emerging laterally from the dendritic shaft were selected without further discrimination. Fluorescence recovery within the ROIs used for the FRAP was then recorded immediately after the bleaching sequence during 750 s as follows: every 2.5 s for 50 s, every 5 s for 200 s and every 10 s for 500 s. Observational photobleaching was assessed by observing control nearby spines that were unbleached. Data were plotted as normalized fluorescence intensity vs. time and fitted by a nonlinear regression (F = (1−IM_f_) (1−exp(−t/τ))) where F is SEP average fluorescence intensity, IM_f_ is the immobile fraction and τ the time constant. Each value of average intensity for SEP signal was normalized by the average intensity of the baseline and by the average intensity of SEP signal for unbleached control spines. The time constant τ and immobile fraction IM_f_ were calculated for 3 to 5 spines for each category of spine from several neurons.

#### Confocal microscopy and image analysis

For visualization of spines and putative synaptic contacts between CA3 transfected cells, organotypic slices were fixed in 4% PFA with 4% sucrose for > 4 h, washed in PBS, and subsequently mounted in Mowiol. Images were acquired on a commercial Leica TCS SP8 microscope (Leica Microsystems, Manheim, Germany) using a ×63/1.4 NA oil objective and a pinhole opened to 1 Airy unit. Images of 2,048 × 2,048 pixels, corresponding to a pixel size of 80–85 nm, were acquired at a scanning frequency of 400 Hz. The axial step size was set at 0.3 μm. Spine morphology was analyzed from 2D projections of confocal image stacks in ImageJ (NIH) using the custom‐written plugin SpineJ as described (Letellier *et al*, 2019; Levet *et al*, 2020). For the analysis, we only considered spines from apical or basal oblique dendrites, which correspond to recurrent synapses made with other CA3 pyramidal cells.

#### mEPSC recording and analysis

Whole‐cell voltage clamp recordings were made from 15 to 16 DIV hippocampal neurons at room temperature in a recording chamber continuously perfused at 2–3 ml min^−1^ with extracellular aCSF solution (pH 7.3, osmolarity adjusted to 290 mOsm) and containing in mM (Sigma‐Aldrich): 130 NaCl, 2.5 KCl, 2.2 CaCl_2_, 1.5 MgCl_2_, 10 D‐glucose and 10 HEPES. aCSF was supplemented with bicuculline (20 μM, Tocris) and TTX (0.5 μM, Tocris). When stated, mEPSCs were recorded in aCSF supplemented with 10 μM *N*‐[3‐[4‐(3‐Aminopropylamino)butylamino]propyl]‐2‐naphthalen‐1‐ylacetamide trihydrochloride (NASPM, Tocris) to selectively block CP‐AMPARs. Transfected neurons were identified with epifluoscence microscopy and differential interference contrast (DIC) illumination using an upright microscope (Nikon Eclipse FN1) equipped with Infinity 3 s camera (Lumenera) driven by Metamorph^®^ software 7.13 (Molecular Devices) and with apochromatic ×60/1.0 NA water objective. Patch pipettes with a resistance range of 5–6 MΩ were made from borosilicate glass capillaries (GC150T‐10, Harvard Apparatus) using a vertical puller (PC‐10, Narishige) and filled with an intracellular solution (pH 7.25, osmolarity adjusted to à 290 mOsm) containing in mM: 135 Cs‐MeSO_4_, 8 CsCl, 10 HEPES, 4 MgATP, 0.3 NaGTP, 5 Qx‐314‐bromide and 0.3 EGTA. The electrodes were subsequently approached to target cells to achieved whole‐cell patch‐clamp using motorized micromanipulators (Scientifica). mEPSCs were recorded from neurons clamped at −70 mV using the multiclamp 700B amplifier (Molecular Devices), digitized at 10 kHz using a Digidata 1440A (Molecular Devices) and acquired using Clampex 10.3 (Molecular Devices). Access resistance (< 20 MΩ) was monitored every 2 min during the recording. Series resistance was left uncompensated. mEPSCs amplitude, frequency, 20–80% rise‐time and decay‐time constant were analyzed using MiniAnalysis software (v6.0, Synaptosoft).

#### Statistical analysis

Graphs and statistical analyses were performed by using GraphPad Prism software. Outliers were identified with the ROUT method on raw experimental data. Data are presented as mean ± SEM. Statistical differences were analyzed as indicated in figure captions. For normally distributed data (as determined by the Agostino‐Pearson omnibus normality test), differences were tested using the two‐tailed Student's *t*‐test and one‐ or two‐way ANOVA test in case of three or more experimental groups. The Mann–Whitney and Kruskal–Wallis tests were used when criteria for normality were not met. Tukey and Dunn's tests were used as multiple‐comparison *post hoc* tests.

## Author contributions

**Sandra Dubes:** Conceptualization; investigation; data curation; formal analysis; writing—review and editing; visualization. **Anaïs Soula:** Investigation; data curation; formal analysis. **Sébastien Benquet:** Investigation; data curation. **Béatrice Tessier:** Methodology; investigation; data curation; formal analysis. **Christel Poujol:** Methodology; resources. **Alexandre Favereaux:** Conceptualization; methodology; investigation; writing—review and editing; supervision. **Olivier Thoumine:** Conceptualization; methodology; writing—review and editing; funding acquisition; supervision; project administration. **Mathieu Letellier:** Conceptualization; methodology; investigation; data curation; formal analysis; writing—original draft; writing—review and editing; visualization; funding acquisition; supervision; project administration.

In addition to the CRediT author contributions listed above, the contributions in detail are:

ML, OT, AF and SD designed the experiments; ML and SD performed and analyzed immunostainings in primary cultures; SD performed and analyzed mEPSC recordings; SD performed and analyzed FRAP experiments; SD performed and analyzed FISH experiments; AS, AF and SB performed and analyzed RT‐qPCR experiments and luciferase assays; BT performed and analyzed Western Blotting experiments; ML performed and analyzed puromycin PLA experiments; ML performed and analyzed experiments in organotypic slices; CP designed the automated image analysis workflow in Metamorph; AF, BT and SB designed and generated DNA constructs; ML wrote the original draft and ML, OT, AF and SD wrote, reviewed and edited the manuscript.

## Disclosure and competing interests statement

The authors declare that they have no conflict of interest.

## Supporting information




Appendix

Expanded View Figures PDF

PDF+

## Data Availability

This study includes no data deposited in external repositories.
