# Supplementary material for: miR‐124‐dependent tagging of synapses by synaptopodin enables input‐specific homeostatic plasticity
Source: EMBO J. 2022 Jul 25;41(20):e109012. doi: 10.15252/embj.2021109012 (PMC9574720; doi:10.15252/embj.2021109012)
Supplement: Supplementary file 1 — Appendix [file EMBJ-41-e109012-s001.pdf]

Appendix

miR-124-dependent tagging of synapses by synaptopodin enables input-specific homeostatic plasticity

Sandra Dubes[1], Anaïs Soula[1], Sébastien Benquet[1], Béatrice Tessier[1], Christel Poujol[2], Alexandre Favereaux[1], Olivier Thoumine[1]*, Mathieu Letellier[1]*#

Table of contents

Appendix Figure S1. Culturing hippocampal neurons in Neurobasal-containing medium occludes HSP.

Appendix Figure S2. Specificity of AMPAR immunostaining in neurons.

Appendix Figure S3. miR-124 is detected at both somatic and dendritic level in cultured hippocampal neurons.

Appendix Figure S1. Culturing hippocampal neurons in Neurobasal-containing medium occludes HSP.

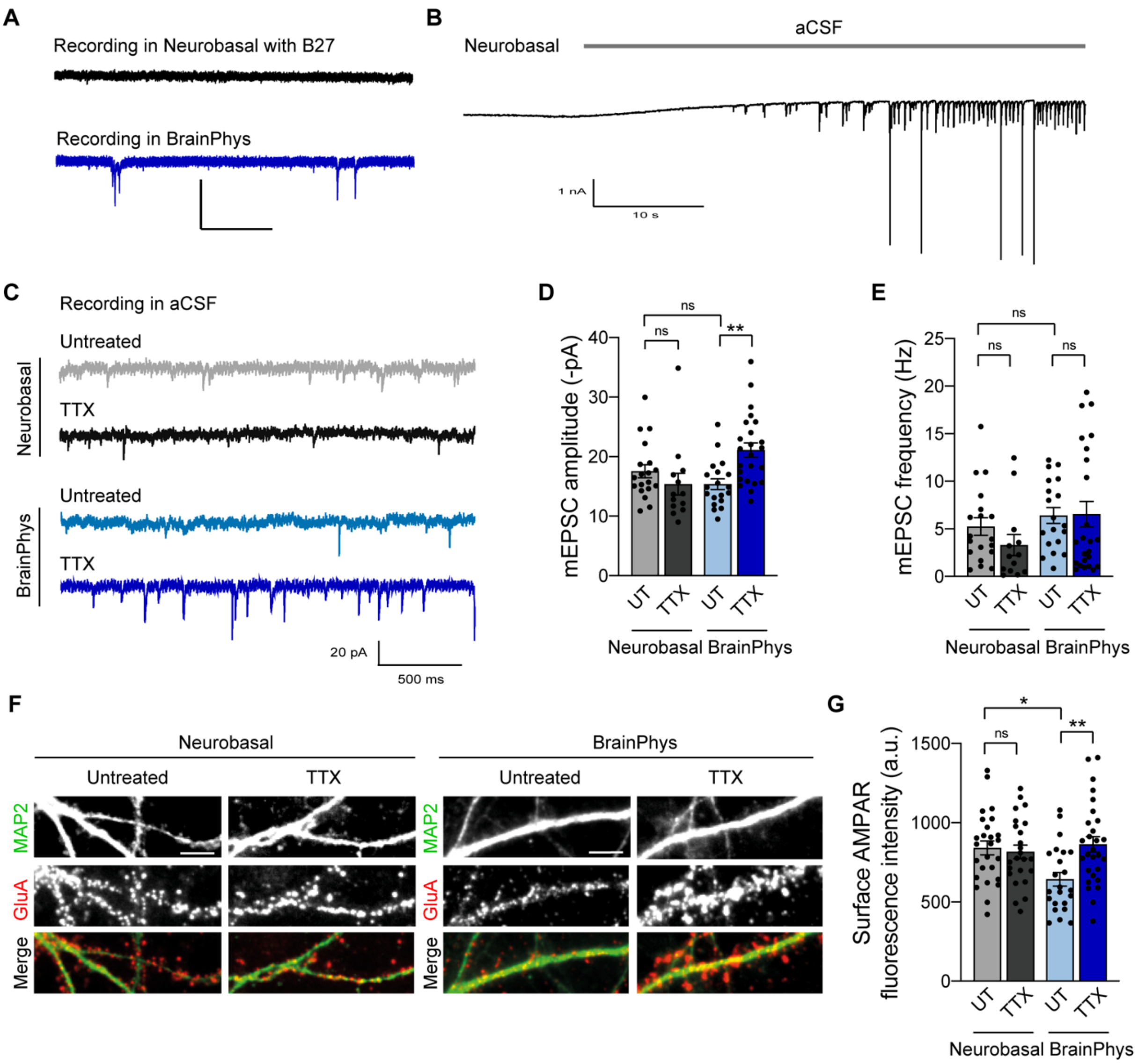

(A) Representative traces of spontaneous synaptic currents recorded either in Neurobasal or BrainPhys-containing medium.

(B) Recording of spontaneous activity from a neuron while washing-out Neurobasal-containing medium with articial cerebro-spinal fluid (aCSF).

(C) Representative traces of AMPAR-mediated miniature currents (mEPSCs) recorded from neurons cultured in Neurobasal or BrainPhys and treated with TTX or not (untreated, UT).

(D,E) AMPAR-mEPSC average amplitudes (D) and frequencies (E) for same condition as in (C) (Neurobasal: UT, n = 19, TTX, n = 13, BrainPhys: UT, n = 19, TTX, n = 24; n indicates cells from 3 cultures). n indicates the number of cells. **P < 0.01, ns, not significant, P > 0.05 by Kruskal-Wallis test followed by Dunn's multiple comparison test.

(F) Micrographs showing immunostaining for MAP2 (green) and surface endogenous AMPARs (red) for same conditions as in (C-E). Scale bar: 5 µm.

(G) AMPAR synaptic fluorescence intensity for same condition as in (F) (Neurobasal: UT, n = 26, TTX, n = 24; BrainPhys: UT, n = 23, TTX, n = 28; n indicates cells from 3 cultures). **P < 0.01, *P < 0.05, ns, not significant, P > 0.05 (two-way ANOVA test followed by Tukey's multiple comparison test).

Data represent mean ± SEM.

Appendix Figure S2. Specificity of AMPAR immunostaining in neurons.

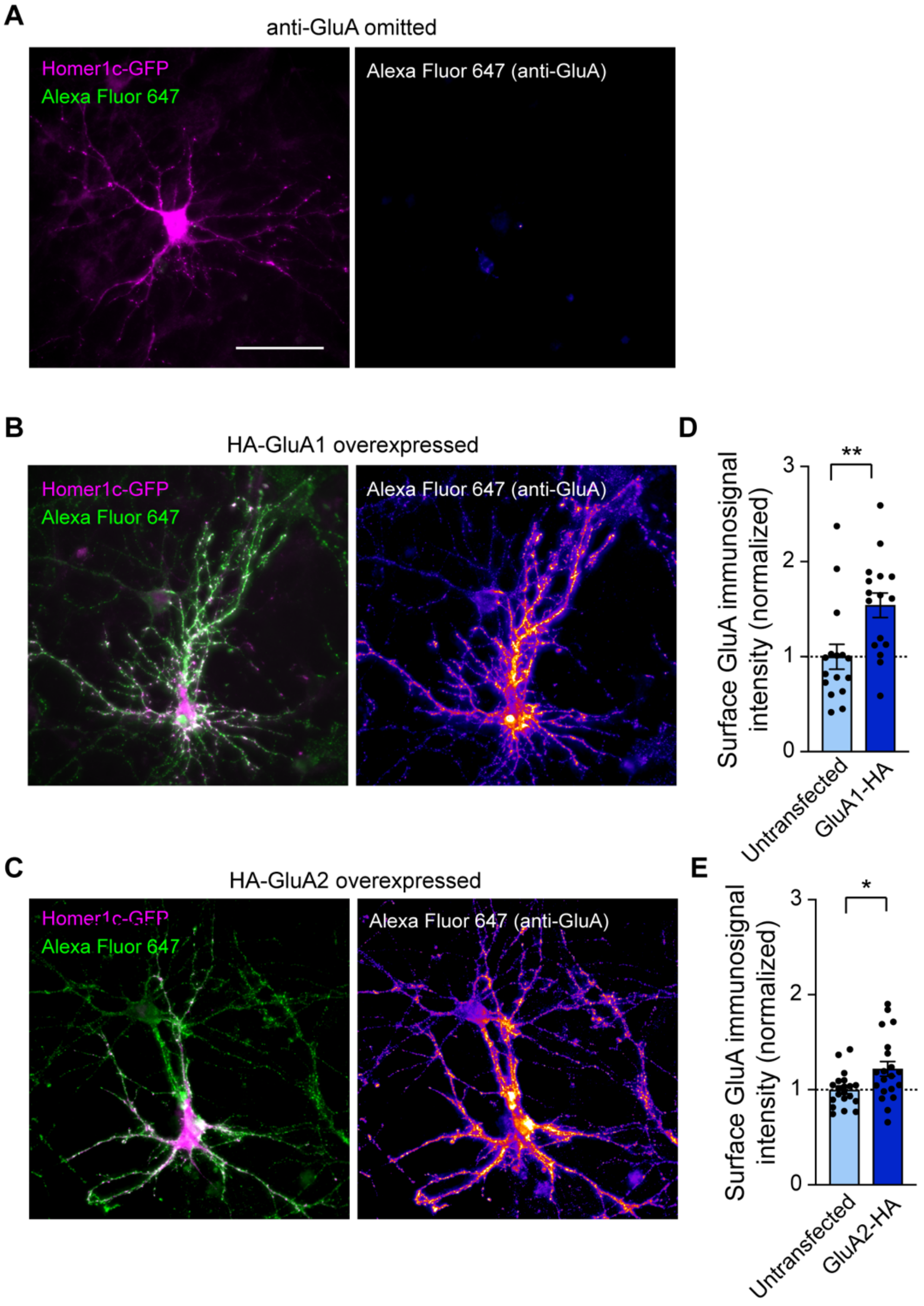

(A) Micrographs showing neurons transfected with Homer1c-GFP (magenta) when anti-GluA antibody was omitted before incubation with secondary antibody conjugated to AlexaFluor 647 (green). On the right panel, Alexa Fluor 647 signal has been coded with pseudo-colors. Scale bar: 60 µm.

(B,C) Micrographs showing neurons transfected with Homer1c-GFP (magenta) and either GluA1-HA (B) or GluA2-HA (C), and immunostained for surface AMPARs with anti-GluA primary antibody and secondary antibody conjugated to Alexa Fluor 647 (green). On the right panel, Alexa Fluor 647 signal has been coded with pseudo-colors. Scale bar: 60 µm.

(D,E) Quantification of surface GluA fluorescence intensity from untransfected vs transfected neurons expressing GluA1-HA (D) or GluA2-HA (E). GluA fluorescence intensity was normalized to untransfected condition (GluA1-HA: untransfected, $n = 16$, transfected, $n = 16$; GluA2-HA: untransfected, $n = 20$, transfected, $n = 20$; n represents the number of cells from 1 culture). GluA1-HA: $^{**}P = 0.0039$ (Mann Whitney test); GluA2-HA: $^{*}P = 0.0160$ (unpaired t test).

Data represent mean ± SEM.

Appendix Figure S3. miR-124 is detected at both somatic and dendritic level in cultured hippocampal neurons.

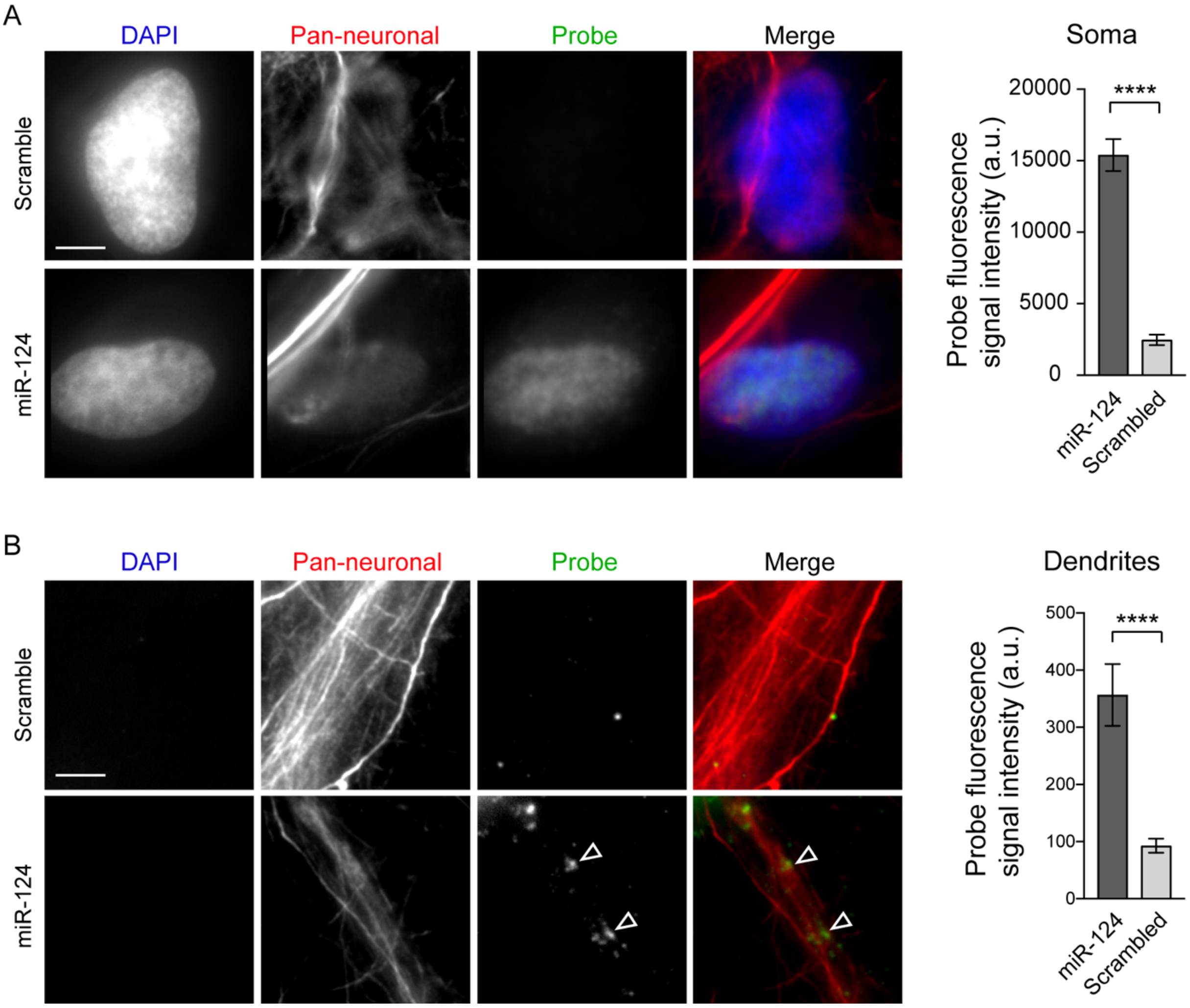

Micrographs showing fluorescent *in situ hybridization* of miR-124 or a control scramble probe (green) in soma **(A)** and dendrites **(B)** of neurons stained with DAPI (blue) and a pan-neuronal marker (red) to visualize neurites. Scale bar: 8 µm. Arrowheads indicate miR-124 puncta in neurites. Graphs (right) represents average fluorescence intensity of the probes within the soma (A) or dendrites (B) (soma: miR-124, n = 20; scrambled probe, n = 26; dendrite: miR-124, n = 9; scrambled, n = 16; n indicates the number of cells from 3 cultures). ****P < 0.0001 (soma: Mann-Whitney test; dendrites: unpaired t test).
