# Supplementary material for: miR‐124‐dependent tagging of synapses by synaptopodin enables input‐specific homeostatic plasticity
Source: EMBO J. 2022 Jul 25;41(20):e109012. doi: 10.15252/embj.2021109012 (PMC9574720; doi:10.15252/embj.2021109012)
Supplement: Supplementary file 2 — Expanded View Figures PDF [file EMBJ-41-e109012-s004.pdf]

Expanded View Figures

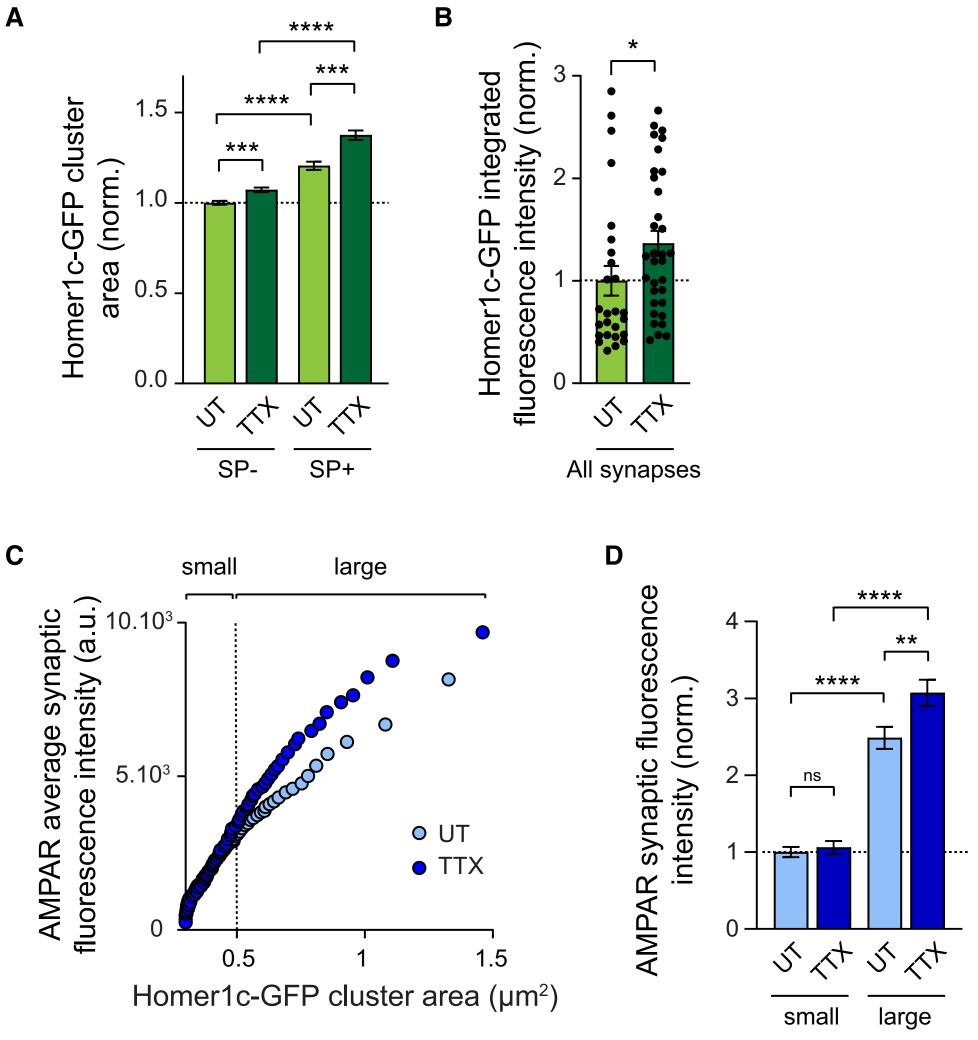

Figure EV1. Synapse size correlates with SP expression and predicts synaptic AMPAR content depending on network activity.

A Area of Homer1c-GFP clusters containing SP (SP^+) or not (SP^-) from neurons treated with TTX or left untreated (UT). Homer1c-GFP cluster area was normalized to untreated SP^- synapse condition (SP^-: UT, n = 1,455, TTX, n = 1,529; SP^+: UT, n = 516, TTX, n = 724; n indicates the number of synapses from three cultures). ***$P < 0.001$, ****$P < 0.0001$ (Kruskall–Wallis test followed by Dunn's multiple comparison test).

B Integrated fluorescence intensity of Homer1c-GFP clusters in UT or TTX-treated neurons, regardless of the expression of SP (all synapses) (UT: n = 26; TTX: n = 33, n indicates the number of cells, from three cultures). Homer1c-GFP cluster area was normalized to untreated condition. *$P = 0.019$ (Mann–Whitney test).

C Plots showing synaptic AMPAR fluorescence intensity vs. synapse size for neurons treated with TTX (dark blue) or untreated (UT, light blue). The two curves were fitted using linear equations and the convergence of the traces to a common fit was tested using the extra sum of squares F test. The F test indicates that the traces are best fitted by two divergent linear models ($P < 0.0001$). Small and large synapses were defined according to the area of Homer1c-GFP clusters with a cut-off set at 0.5 μm^2.

D AMPAR synaptic fluorescence intensity at small vs. large synapses in UT and TTX-treated neurons. AMPAR synaptic fluorescence intensity was normalized to small synapses from UT condition (UT: SP^-, n = 1,455, SP^+, n = 516; TTX: SP^-, n = 1,529, SP^+, n = 724, n indicates the number of synapses, from three cultures). **$P < 0.01$, ****$P < 0.0001$, ns, not significant, $P > 0.05$ (Kruskal–Wallis test followed by Dunn's multiple comparison test).

Data information: Data represent mean ± SEM.

© 2022 The Authors

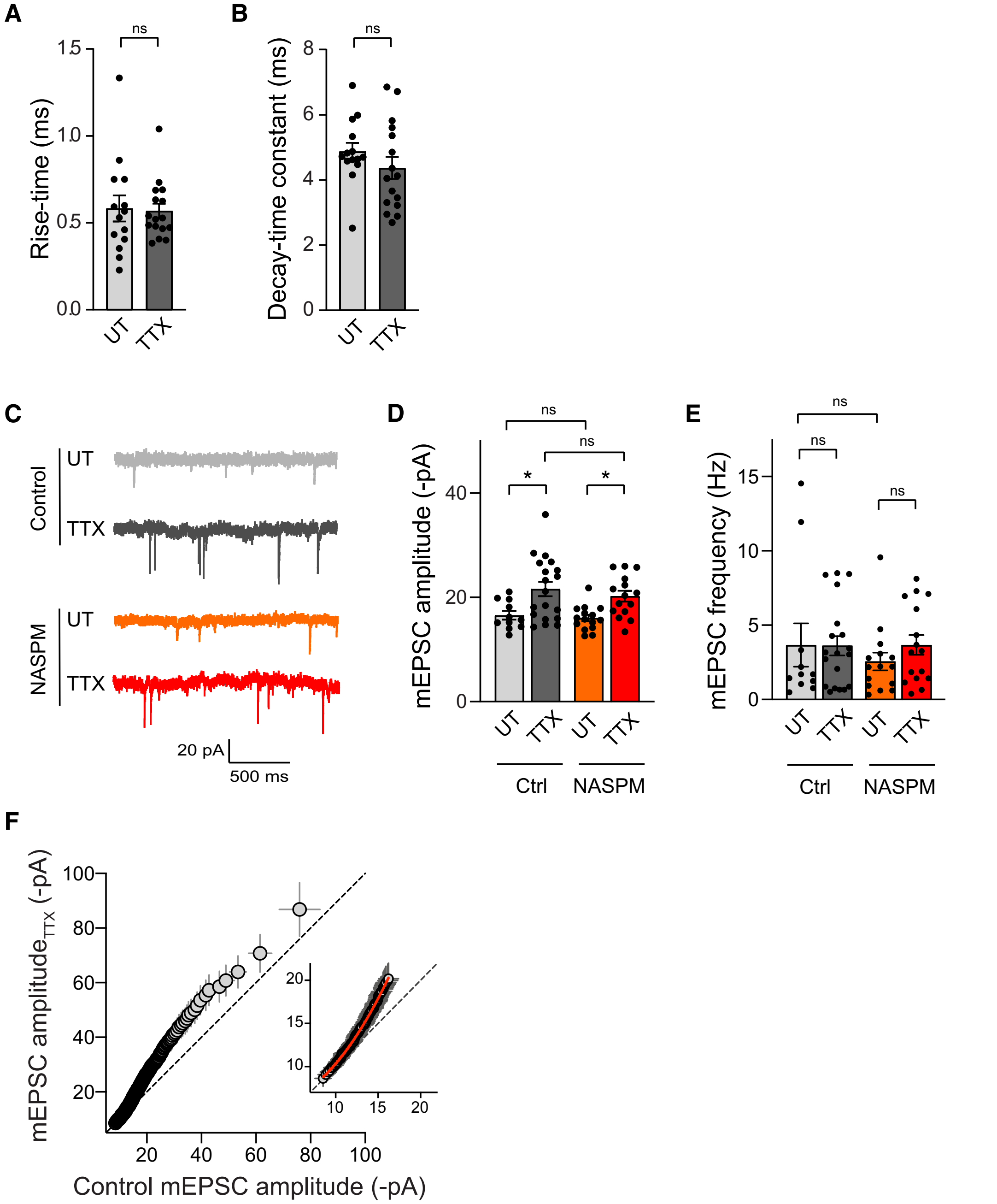

Figure EV2. TTX-induced upscaling is not accompanied by change in mEPSC kinetics or sensitivity to NASPM.

A, B Rise-time (A) and decay-time constant (B) for neurons treated with TTX or left untreated (UT) (UT: n = 14, TTX, n = 16; n indicates the number of cells from three cultures). $P > 0.05$, ns, not significant (Mann Whitney test).

C Representative traces of AMPAR-mediated mEPSCs from neurons treated with TTX, or untreated (UT) and recorded with or without (control, Ctrl) 10 µM NASPM.

D, E Mean mEPSC amplitudes (D) and frequencies (E) for each condition (Ctrl: UT, n = 11, TTX, n = 19; NASPM: UT, n = 15, TTX, n = 15; n indicates the number of cells from three cultures). *$P < 0.05$, ns, not significant, $P > 0.05$ by two-way ANOVA test followed by Tukey's multi comparison test (D) or Kruskal–Wallis test (E).

F Plot showing the rank-ordered AMPAR-mediated mEPSC amplitudes measured in TTX-treated neurons vs. UT neurons (200 events). The rank-order plot was obtained by sorting from smallest to largest amplitude in untreated and TTX data and plotting them against each other. The extra sum of squares F test indicates that the first 100 events are better fitted with a second-order polynomial quadratic curve (in red, TTX = 6.00 – 0.29 × UT + 0.07 × UT^2; R^2 = 0.99; ****$P < 0.0001$) than with a linear regression (not shown, TTX = –5.23 + 1.53 × UT).

Data information: Data represents mean ± SEM.

 © 2022 The Authors

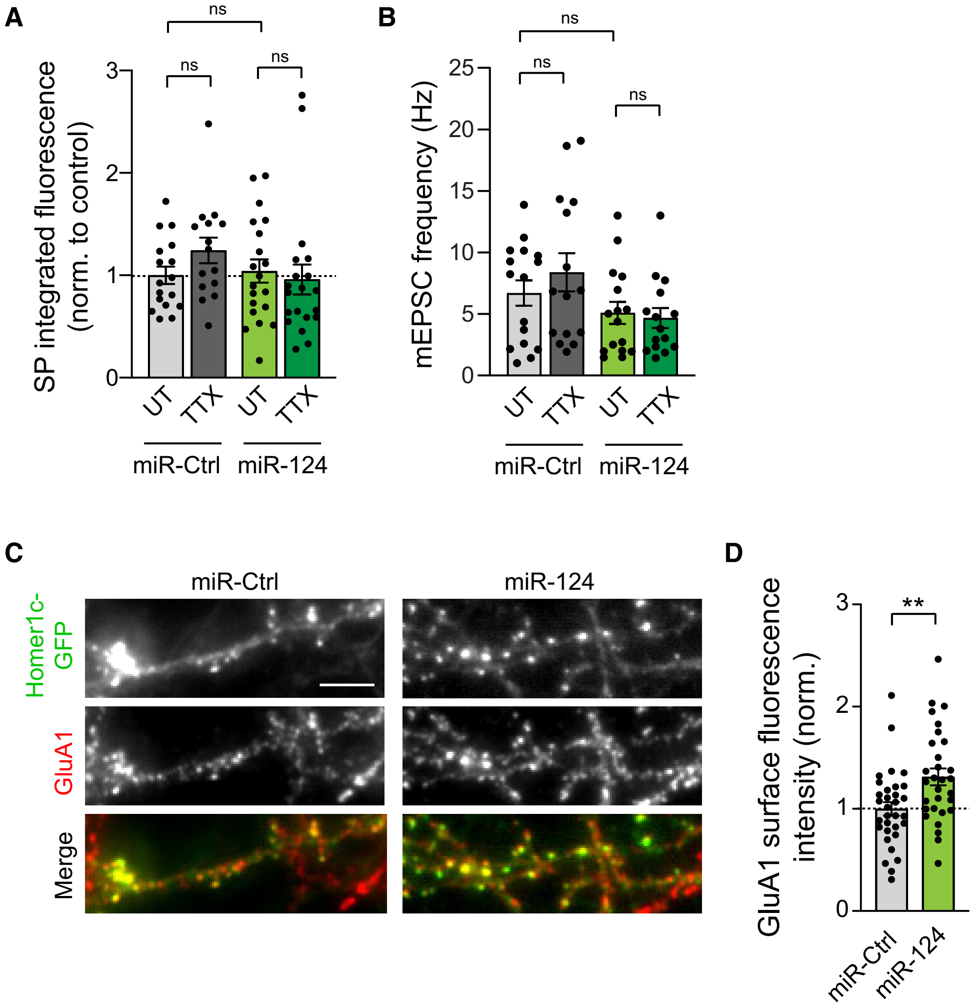

Figure EV3. Effect of miR-124 overexpression on SP expression, mEPSCs frequency and GluA1 synaptic expression.

A SP integrated fluorescence intensity in neurons expressing Homer1c-GFP and either miR-124 or control miR-67 (miR-Ctrl), and treated with TTX, or left untreated (UT). SP integrated fluorescence intensity was normalized to untreated miR-Ctrl condition (miR-Ctrl: UT, $n = 17$, TTX, $n = 15$; miR-124: UT, $n = 20$, TTX, $n = 20$; n indicates the number of cells from two cultures). ns, not significant, $P > 0.05$ (Kruskal–Wallis test).

B mEPSCs frequency for same conditions as in (A) (miR-Ctrl: UT, $n = 16$, TTX, $n = 15$, miR-124: UT, $n = 16$, TTX, $n = 15$; n indicates the number of cells from four cultures). ns, not significant, $P > 0.05$ (Kruskal–Wallis test).

C Micrographs showing neurons expressing Homer1c-GFP (green) and either miR-Ctrl or miR-124, and immunostained for surface GluA1 (red) in untreated neurons. Scale bar: 5 μm.

D Surface GluA1 fluorescence intensity for same conditions as in (C). GluA1 surface intensity was normalized to miR-Ctrl condition (miR-Ctrl: $n = 32$; miR-124: $n = 30$; n indicates the number of cells from two cultures). **$P = 0.041$ (two-tailed unpaired t test).

Data information: Data represents mean ± SEM.

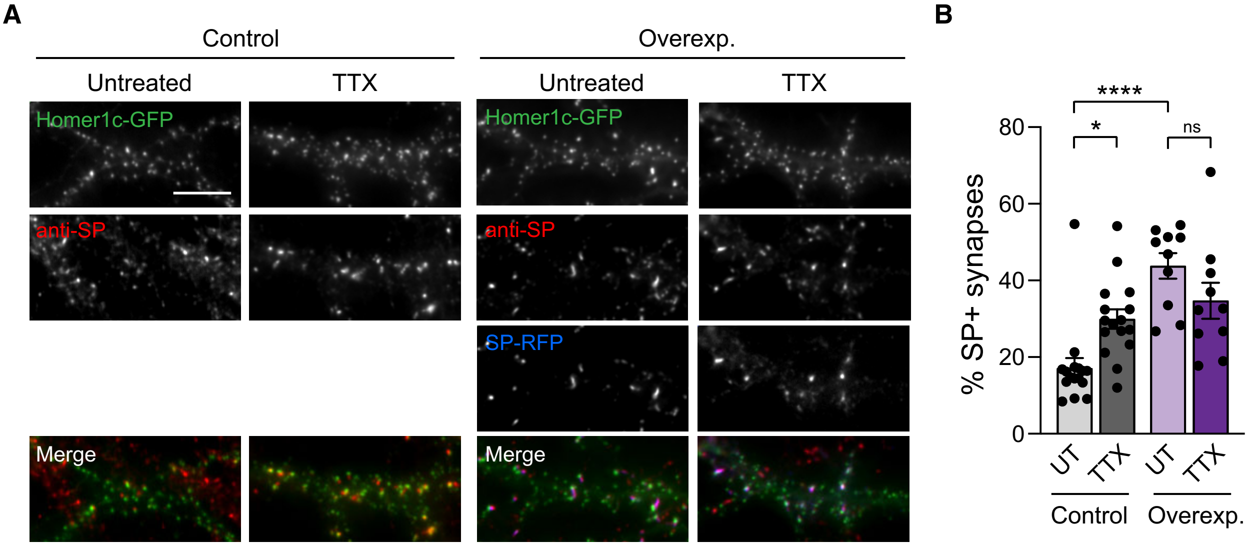

Figure EV4. TTX-induced increase in the percentage of SP$^+$ synapses is occluded by SP overexpression.

A Micrographs showing transfected neurons with Homer1c-GFP alone (control, green) or with SP-RFP (overexp., blue), and treated with TTX, or left untreated (UT). SP (endogenous and exogenous) was immunostained using anti-SP antibody (red). Scale bar: 10 µm.

B Percentage of SP$^+$ synapses for each condition (Control: UT, n = 16, TTX, n = 16, Overexp.: UT, n = 10, TTX, n = 10; n indicates the number of the cells from). *$P < 0.05$, ****$P < 0.0001$, ns, not significant, $P > 0.05$ (two-way ANOVA test followed by Tukey's multiple comparison test).

Data information: Data represent mean ± SEM.

 © 2022 The Authors

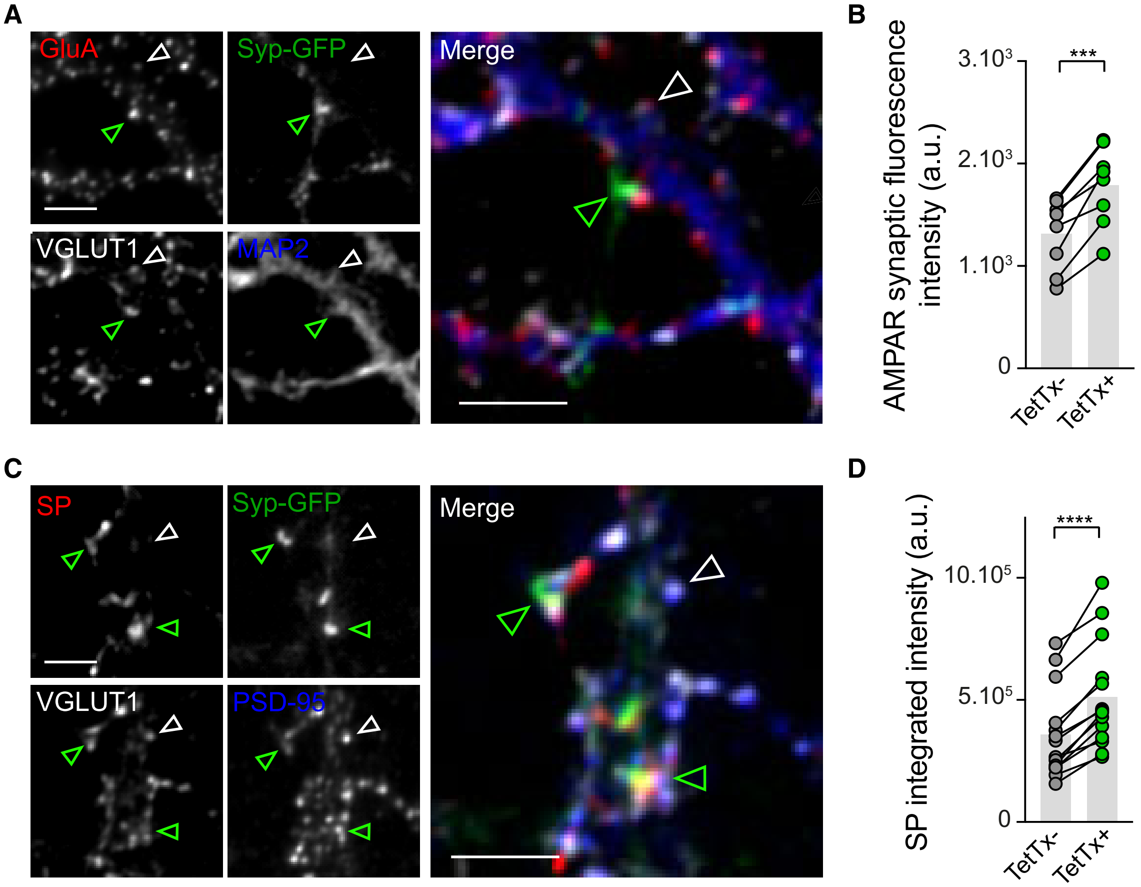

Figure EV5. Synapse-autonomous homeostatic regulation of endogenous AMPARs and SP upon presynaptic silencing in cultured hippocampal neurons.

A Micrographs showing dendrites from cultured hippocampal neurons immunostained for MAP2 (blue), VGLUT1 (gray) and AMPAR (red) and contacted by presynaptic terminals expressing Syp-GFP + TetTx (green). Arrowheads indicate GFP^+ (green) and GFP^- (white) terminals, immunopositive for VGLUT1. Scale bar: 5 μm.

B AMPAR synaptic fluorescence intensity of clusters apposed to $TetTx^-$ vs. $TetTx^+$ terminals (n = 8 cells from three cultures). ****P < 0.0001 (Mann Whitney test).

C Micrographs showing dendrites from cultured hippocampal neurons immunostained for endogenous PSD-95 (blue), VGLUT1 (gray) and SP (red) and contacted by presynaptic terminals expressing Syp-GFP + TetTx (green). Arrowheads indicate GFP^+ (green) and GFP^- (white) terminals, immunopositive for VGLUT1. Scale bar: 5 μm.

D SP integrated fluorescence intensity of clusters apposed to $TetTx^-$ vs. $TetTx^+$ terminals (n = 11 cells from three cultures). ****P < 0.0001 (Mann Whitney test).

Data information: Data represent mean ± SEM.

© 2022 The Authors
