# Supplementary material for: miR‐124‐dependent tagging of synapses by synaptopodin enables input‐specific homeostatic plasticity
Source: EMBO J. 2022 Jul 25;41(20):e109012. doi: 10.15252/embj.2021109012 (PMC9574720; doi:10.15252/embj.2021109012)
Supplement: Supplementary file 4 [file EMBJ-41-e109012-s002.pdf]

THE EMBO JOURNAL

miR-124-dependent tagging of synapses by synaptopodin enables input-specific homeostatic plasticity

Sandra Dubes, Anaïs Soula, Sébastien Benquet, Béatrice Tessier, Christel Poujol, Alexandre Favereaux, Olivier Thoumine, and Mathieu Letellier
DOI: 10.15252/embj.2021109012

Corresponding author(s): Mathieu Letellier (mathieu.letellier@u-bordeaux.fr)

Review Timeline:

Submission Date:	18th Jun 21
Editorial Decision:	27th Jul 21
Revision Received:	14th Mar 22
Editorial Decision:	27th Apr 22
Revision Received:	11th Jun 22
Accepted:	27th Jun 22

Editor: Karin Dumstrei

Transaction Report:

(Note: With the exception of the correction of typographical or spelling errors that could be a source of ambiguity, letters and reports are not edited. Depending on transfer agreements, referee reports obtained elsewhere may or may not be included in this compilation. Referee reports are anonymous unless the Referee chooses to sign their reports.)

Dear Dr. Letellier,

Thank you for submitting your manuscript to The EMBO Journal. Your study has now been seen by three referees and their comments are provided below.

As you can see from the comments, the referees find the analysis interesting. However, they also raise a number of important concerns that should be resolved before further consideration here. Should you be able to address the raised concerns in full then I would like to invite you to submit a revised version. I think it would be helpful to discuss the raised points further and I am available to do so via email or video. Let me know what works best for you.

When preparing your letter of response to the referees' comments, please bear in mind that this will form part of the Review Process File, and will therefore be available online to the community. For more details on our Transparent Editorial Process, please visit our website: https://www.embopress.org/page/journal/14602075/authorguide#transparentprocess

I thank you for the opportunity to consider your work for publication. I look forward to discussing the revisions further with you.

PS I have attached a document with helpful tips on how to prepare the revised version. Please pay attention to the parts on the Data Availability Section and figure legends.

with best wishes

Karin

Karin Dumstrei, PhD
Senior Editor
The EMBO Journal

Guide For Authors: https://www.embopress.org/page/journal/14602075/authorguide

Revision to The EMBO Journal should be submitted online within 90 days, unless an extension has been requested and approved by the editor; please click on the link below to submit the revision online before 25th Oct 2021:

https://emboj.msubmit.net/cgi-bin/main.plex

Referee #1:

In the present manuscript, Dubes et al. report a novel microRNA-dependent tagging mechanism in homeostatic plasticity. Although there is some evidence that local mechanisms are involved in homeostatic synaptic upscaling, it is still unclear how specific synapses are selected for upscaling and which molecular changes could work as a "tag" for such a selective recruitment. Therefore, the elucidation of such a mechanism would represent a major advance in the field. In their experiments, the authors convincingly establish differential accumulation of synaptopodin (SP) and subsequent trapping of GluA2 containing AMPA-Rs as a potential tagging mechanism. Furthermore, using a rather elegant replacement strategy in combination with microscopy and electrophysiology, they obtain evidence for an important contribution of miR-124-dependent SP translation in this process.
However, in order to be published in EMBO J., the authors should perform additional experiments to strengthen the link between the differential upscaling of synapses and miR-124-dependent SP translation. In particular, as outlined in more detail below, they need to confirm the role of the endogenous miR-124 in local dendritic SP translation and synaptic upscaling to support the physiological relevance of their findings.

Major points.
1. The authors convincingly show that over expression of miR-124 prevents accumulation of SP and upscaling of AMPARs at silenced synapses. From this, the authors conclude that miR-124 dependent local translation of SP is driving differential upscaling. More experiments are needed to verify this model. First, they need to provide direct evidence for local translation of SP during synaptic upscaling. This could be addressed for example by performing Puro PLA proximity ligation assay (Dieck et al, Nat methods 2015) to determine if SP is locally translated in dendrites/spines upon TTX treatment. The authors have a nicely specific Sp antibody, which is a prerequisite to establish the PLA assay. In addition, such experiments would also give insight into a preferential translation of SP at large vs. small spines. Second, this assay could also be used to assess how overexpression or inhibition of miR124 impact SP translation. This would nicely complement and strengthen the results of

replacing endogenous SP with a recombinant protein derived from a vector with and without a functional miR-124 binding site (see also 2.).
2. Many of the crucial conclusions are based on their elegant replacement strategy (KD of SP in combination with re-expression from a vector containing either a wild type or mutated 3' UTR, fig.6 and 8). However, this approach relies on the ectopic expression of SP, which could introduce artifacts. Therefore, it is necessary to have an independent approach that shows that endogenous miR-124 regulates endogenous SP during upscaling. In this respect, the most elegant approach would be to use a target protector LNA that prevents the endogenous miR-124 from binding to the endogenous SP, followed by SP immunostaining. This would provide conclusive evidence with regards to the functional importance of this miRNA-target interaction. Alternatively, this experiment could be performed with a miR-124 LNA, which however would not specifically address the association between miR-124 and SP. In any case, in my opinion it is essential to perform miR-124 loss-of-function approaches under basal and silenced condition in vitro and ex vivo to strengthen the results shown in Fig. 6 and 8.
3. Since the dendritic localization of miR-124 is somewhat controversial, showing convincing FISH data in dendrites of control- and TTX-treated neurons is imperative. However, the quality of the miR-124 FISH data (fig S7) provided does not allow to draw any conclusions, so this needs to be repeated. This analysis should be complemented by a confirmation of the dendritic localization of SP. Lastly if dendritic localization of miR-124/SP can be confirmed confirmed, then correlating the distribution of miR-124 to endogenous SP protein +/- TTX would be an interesting piece of data.

More minor points
4. Why were two different statistical methods used in fig. 3E and H? please explain.
5. An empty vector is not an appropriate control for the SH-RNA experiments performed in figure 3. This should be replaced with a vector expressing an shRNA of an unrelated (scrambled) sequence. The multiple rescue experiments are reassuring with regards to the specificity of the effects observed, but at least one experiment comparing the effect of the Sp-sh to a control vector expressing a scrambled Sh sequence should be performed.
6. In Fig. 4, Sp regulation by endogenous miR-124 should also be validated in luciferase assays (TTX +/- pLNA)
7. In Fig. 5, It would be informative to know whether blocking miR-124 is sufficient to induce HSP at specific synapses (via induction of Sp expression)?
8. In their model, the authors postulate that the miR-124-Sp-mRNA complex is selectively active at large synapses. This in turn would invoke a selective recruitment of this complex. The authors should provide some discussion about possible mechanism(s) which could play a role in the selective recruitment of miRNA-target complexes to specific types of synapses.

Additional, non-essential suggestions:
the study would greatly benefit from life imaging, e.g assessing the accumulation of SP during TTX-induced upscaling at specific spines over time. However, this might be technically difficult over extended periods of time (e.g. 48 h) and therefore beyond the scope of the present study.

Referee #2:

In this paper, the authors propose an interesting role for miR-124 regulation of synaptopodin expression in homeostatic synaptic plasticity (HSP) and AMPAR recruitment to synapses. The work provides novel mechanistic insight into synaptic regulation during HSP, with the additional interest of a synapse-specific form of HSP. However, there are a number of issues that should be addressed prior to publication, as described below.

Fig 2:
I found it surprising that the authors used neurons at DIV10 for the experiments imaging surface GluA2 diffusion. The experiments presented in Fig 1, 3, etc used neurons at DIV15. More specifically, it is surprising that neurons at DIV10 have mature synapses and spines. Is this a characteristic of neurons cultured in BrainPhys?
The authors should include a statement justifying why immature neurons are used for this experiment, supporting evidence for SP expression, and (if the alternative culture medium is involved) an analysis of spine/synapse development in neurons cultured in this way.
Also, spine morphology is likely to be a critical determinant of AMPAR surface diffusion into or out of spines. The authors need to include a statement defining what morphological category of spines were analysed.
The bleach area/recording area is not well defined. Was the whole spine bleached and subsequently analysed? This must be defined more accurately.

Fig 5:
Some of these data, which appear to be fundamentally important to the conclusions of the study, are unconvincing. The authors state there is a "trend" in synaptic recruitment of AMPARs in response to TTX. A P-value of 0.47 is not even close to statistical significance. This is not a significant effect, and the effect of miR-124 is also non-significant. I acknowledge the mEPSC data and their statistical significance, so can the authors provide a strong explanation for the results in Figs 5c and d?

Fig.7:
In these, and other experiments in which the knockdown/rescue strategy is used, the SP present in neurons will be 30-40% RFP-SP with recombinant 3'UTRs, and 60-70% endogenous SP. Can this relatively small contribution of the recombinant gene

account for all the effects seen in Figs 6, 7 and 8? This must be discussed.

I don't understand how the authors come to the conclusion, "This effect was accompanied by a selective increase of size for the largest spines while the smallest spines remained of similar size". This implies that spines were measured before transfection with the molecular replacement constructs, and then the same spines were analysed afterwards. How were the same spines identified several days after transfection? And how were spine sizes measured before transfection in the absence of a fluorescent marker? Maybe I'm missing something here, but this needs to be clarified.

Fig. 8h: the authors refer to a partial occlusion of the effect of synaptic silencing by expressing the mutated SP-3'UTR. For this to be an acceptable conclusion, the difference between SP-WT and SP-MUT in the absence of TetTx must be statistically significant. However, this essential comparison is not included on the graph. This must be rectified.
Also, why are there such massive differences in sample size for the different conditions? The TetTX- conditions have n=220 and 616, while the TetTX+ conditions have n=25 and 26. I wonder if the authors were pushing for a statistically significant difference between SP-WT and SP-MUT in the absence of TetTX, hence they conducted hundreds of repeats. Perhaps if SP-MUT in the presence of TetTX had a comparable number of repeats, this difference might also be significantly different? This should be tested.

Minor points:
Fig 4c:
Is the miR-124 binding site really in position 1574-1580 in both Gria2 and synaptopodin mRNA? If this is just a typo, it should be corrected.

Fig. S7A,B:
The authors state, "staining was absent at both somatic and dendritic levels when using a control scramble probe, demonstrating the specificity of the signal." There is clearly a punctum present in control dendrites, hence this claim needs to be modified.

Referee #3:

The manuscript by Dubes et al. describes how mir-124-dependent expression of synaptopodin regulates input-specific homeostatic scaling of synapses in rat hippocampal neurons. This is an important and elegantly designed study. Although overall data are solid and support the hypothesis, there are still some gaps which should be filled at the experimental or editorial level. I would recommend the paper for publication after thorough revision.

General points:
1) Some of Homer1c fusion constructs seem to have altered localization and no longer can be considered as specific postsynaptic marker: the construct seems to be all over the dendrites in some example images (e.g. Fig. 6A, Fig. S2). How was it even possible to calculate the homer1c-clusters in such a case? Although previous studies has not found any effect of Homer1c overexpression on PSD- and spine-size (e.g. Meyer, D., Bonhoeffer, T., and Scheuss, V. (2014). Neuron 82, 430-443), there might be other parameters affected, as overexpression of proteins always introduces a disturbance to cellular homeostasis, as well as levels of overexpression differing between different cells, this has the potential to introduce a lot of "noise" to the experiments. It is unclear why the authors chose to use overexpression as opposed to staining endogenous Homer1, since most of the data shown is from fixed cells where overexpression seems unnecessary for the assay setup.

2) It is not described clearly in the methods & protocols how the intensity measurements of synapse-components (SP, AMPAR) were performed. For example, it is unclear whether the intensity was measured in a single plane, or whether the authors recorded z-stacks to image the entire dendritic volume - the latter would yield way more precise results, as imaging a single z-plane entails the risk of under-estimating fluorescence intensity if the signal is slightly out of focus, as well as introducing an additional level of error. Further, it says in line 732 that "we estimated the percentage of Homer1c-GFP clusters for which at least 20 % of pixels was also positive for SP; these clusters were considered as SP+ synapses". What does it mean "we estimated"? How was it estimated? Please describe your data analysis workflow in more detail. It would help to include an example image of the analysis workflow where the detected homer-clusters are outlined. Also, the way the workflow is described in methods & protocols, it seems the authors did not discriminate at all between excitatory shaft-synapses and spine-synapses for any of the analyses? If so, this should be clearly stated in the text, since there are clear differences in functionality between those two types of synapses, and this has implications for the interpretation of the data.

3) Sometimes n-numbers in graphs represent cells (e.g. Fig. S6), sometimes individual spines (e.g. Fig. S8), i.e. the n-number is

very high, making even small changes "statistically significant". Would it make more sense to average all results per cell, to make the analysis more robust? If not, please argue why it would make sense to regard a single spine as "one n" in some experiments and not in others. Also, please indicate the number of independent experiments / neuronal preparations for the individual experiments. In methods & protocols it is merely stated "Data are presented as mean {plus minus} SEM of three or more experiments performed in independent preparations". Please indicate the exact number for each experiment in the figure legends.

Specific points:

1. Figure 1 and corresponding results: Fig. 1A: Please put the corresponding channel labels (SP, GluA, Merge..) also into the images under the TTX heading to make it easier to grasp everything at a glance. The "untreated" example of synaptopodin staining does not really represent the average 25% of SP+ spines shown in 1B; either the contrast in the SP panel is too low, or it is a bad example.
Line 134 / Fig. 1E: "increase in AMPAR synaptic content observed for TTX-treated neurons compared to untreated ones was not multiplicative and selectively occurred at synapses with the highest AMPAR content" -> it is not immediately clear how the cumulative distribution and the scaling factor shown in Fig. 1E prove this statement. The meaning of the scaling factor or how it was obtained is not explained at all the first time it is introduced (Fig. 1E), but only later for Fig. 1H. Please include one or two explanatory sentences on how the data was analyzed and interpreted.
Line 156: "Consistent with this finding, the rank-ordered mEPSC amplitudes from TTX-treated cells plotted against rank-ordered mEPSC amplitudes from control cells were better fitted with a quadratic function than with a linear function (Fig. S4F)": This is unnecessary statement, because every dataset will be better fitted with a quadratic function than a linear function, because there is simply an additional variable in a quadratic function.

2. Figure 2 and corresponding results: The RFP-synaptopodin construct used in Fig. 2, as well as similar constructs used in literature cited by the authors (e.g. Vlachos et al, 2009; Konietzny et al, 2019), does not contain the 3'UTR of the endogenous synaptopodin, which the authors show later is important for the regulation of local synaptopodin translation. Can the authors speculate on how the regulation of such an overexpression constructs, which seems to behave very similarly to the endogenous protein in terms of localization and abundance in spines, could be mediated despite the lack of the 3'UTR regulatory element? (The authors later show that overexpressed SP-RFP dos not show the same increase upon TTX treatment as endogenous SP)
In line 176 the authors conclude "SP promotes the synaptic stabilization of surface diffusing AMPARs". This conclusion is too specific, as (they later mention this in the discussion), the presence of synaptopodin, indicative of a spine apparatus, indiscriminately inhibits the diffusion of (trans)membrane proteins with a large intracellular domain (Wang et al, 2016a). The causal connection between the presence of SP and AMPAR anchoring is not shown, this is only a positive correlation. Therefore, there is no basis for the statement directly after this sentence.

3. Figure 3, S3 and corresponding results: shRNA knock down experiments - it would be good practice to use another shRNA as control, scrambed or against an exogenous gene, instead of an empty vector.
Line 192: "knocking-down endogenous SP in cultured neurons did not alter the basal synaptic accumulation of AMPARs": How do the authors reconcile this? According to Fig 1C, reducing SP should lead to fewer SP+, less AMPAR anchoring and therefore lower overall AMPAR-density.
Line 193: "... these results indicate that the presence of SP is required for synapses to undergo HSP": This statement needs further validation. A 40% reduction of SP does not mean its absence of all synapses. To make this statement, the authors need to show the SP- and SP+ distribution, as in Fig1, upon shRNA knockdown of SP and show that only the SP+ synapses undergo HSP.
FigS3C: Is the y-axis integrated or averaged intensity?

4. Figure 4 and corresponding results: Fig. 4C: Exactly the same experiment + results was shown in Elramah et al. 2017, so it was good that the authors reproduced those results, but this paper should be cited at this point. Also, double-check the sequences, since Elramah et al. indicate the complementarity between miR-124 and SP 3'UTR at position 1614-1620, while the authors here indicate 1574-1580. Where does this discrepancy come from?

5. Figure 7+8 and corresponding results: For experiments in CA3 neurons it should be stated which dendrites were selected for analysis (basal, apical, oblique...). For instance, thorny excrescences contacted by mossy fibers have very different morphology. They are very big, all of them are positive for synaptopodin and they undergo very different type of plasticity. It would be very interesting to look at these spines or to make sure that they were not included in the analysis.

6. Discussion:
Line 372: "However, we report.... synaptic weight distribution": The authors do not track the fates of individual synapses as TTX is applied. The data is solid, but e.g. whether the smallest SP- synapses indeed remain as such and the other slightly larger SP- ones become SP+ is not shown. One could also see the data such that, if one puts the threshold of SP-/SP+ a little lower, that ONLY SP+ synapses are linearly scaled. The manuscript would benefit strongly from such a tracking of fates, but this experiment is not necessarily required.

Minor points:

Line 23: "to a same" -> "to the same"
line 40: "synapses" plural should be synapse singular, "with a same gain" -> "with the same gain"
line 92: "interesting" -> "interestingly"
Line 359 "not only we demonstrate" - "not only do we demonstrate"
Line 317 "apposed" -> "opposed"
In Fig. 1, 3, 5, and 6, the label size inside the microscopy images is too small; it is hard to read. This is partly due to the poor quality of the exported pdf, try to enhance the quality / reduce compression. The colour of the blue channel is hard to make out against the black background. Maybe choose a lighter blue that contrasts better with the black background.
FigS2: It is unclear with which AB this staining was performed. Additionally, no information can be found on the Agrobio AB against GluA1.
Fig. S8A: the white arrowhead indicates two different synaptic terminals in the different panels
For all of the overexpression plasmids used, it would be nice to also specify the respective promoters in the Methods & Protocols. Were all those expressed under the CMV promoter, synapsin, beta-actin or a different one?

Point-by-point response to referees – Dubes et al.

We thank the referees for their highly constructive and helpful comments. Our responses are shown below in blue font.

Referee #1:

In the present manuscript, Dubes et al. report a novel microRNA-dependent tagging mechanism in homeostatic plasticity. Although there is some evidence that local mechanisms are involved in homeostatic synaptic upscaling, it is still unclear how specific synapses are selected for upscaling and which molecular changes could work as a "tag" for such a selective recruitment. Therefore, the elucidation of such a mechanism would represent a major advance in the field. In their experiments, the authors convincingly establish differential accumulation of synaptopodin (SP) and subsequent trapping of GluA2 containing AMPA-Rs as a potential tagging mechanism. Furthermore, using a rather elegant replacement strategy in combination with microscopy and electrophysiology, they obtain evidence for an important contribution of miR-124-dependent SP translation in this process. However, in order to be published in EMBO J., the authors should perform additional experiments to strengthen the link between the differential upscaling of synapses and miR-124-dependent SP translation. In particular, as outlined in more detail below, they need to confirm the role of the endogenous miR-124 in local dendritic SP translation and synaptic upscaling to support the physiological relevance of their findings.

Major points.

1. The authors convincingly show that over expression of miR-124 prevents accumulation of SP and upscaling of AMPARs at silenced synapses. From this, the authors conclude that miR-124 dependent local translation of SP is driving differential upscaling. More experiments are needed to verify this model. First, they need to provide direct evidence for local translation of SP during synaptic upscaling. This could be addressed for example by performing Puro PLA proximity ligation assay (Dieck et al, Nat methods 2015) to determine if SP is locally translated in dendrites/spines upon TTX treatment. The authors have a nicely specific Sp antibody, which is a prerequisite to establish the PLA assay. In addition, such experiments would also give insight into a preferential translation of SP at large vs. small spines. Second, this assay could also be used to assess how overexpression or inhibition of miR124 impact SP translation. This would nicely complement and strengthen the results of replacing endogenous SP with a recombinant protein derived from a vector with and without a functional miR-124 binding site (see also 2.).

We followed reviewer's suggestion and carried out a Puromycin proximity ligation assay (puro-PLA) to directly visualize newly synthesized SP during HSP in cultured hippocampal neurons. The results, which are summarized in the new Figure 7, support the hypothesis that SP is locally synthesized to support synapse-autonomous HSP.

Taking advantage of our antibody against SP and following a protocol similar to **Dieck et al. (Nat Methods 2015)**, we detected SP Puro-PLA signal in the cell body as well as along

dendrites, consistent with local protein translation. No Puro-PLA signal was detected when omitting the SP antibody or puromycin incubation, validating the specificity of the signal. Interestingly, a small fraction of Homer1c-positive synapses (~5%) was found overlapping with puro-PLA puncta (PLA+ synapses), suggesting that SP can be directly synthesized within spines, possibly reflecting the direct assembly of SP clusters in spines **(Konietzny et al., 2019)**. Incubating neurons for 24 h with TTX induced a significant increase in both the number and the signal intensity of SP puro-PLA clusters along dendrites, suggesting that the number of SP translation sites and the amount of newly synthesized SP at individual sites were both increased. We also found that the percentage of PLA+ synapses was increased by 2-fold (~10%) following the TTX treatment; those synapses were larger in size compared to PLA- synapses, suggesting a preferential translation of SP at large vs small synapses. Altogether, these new results fit perfectly well with our initial immunostaining data: (1) the almost 2-fold increase in the number of SP+ synapses upon TTX treatment (Fig. 1); (2) the correlation between SP expression and synapse size (as well as AMPARs content and diffusion) (Fig 1 and Fig EV1) and (3) the preferential contribution of large versus small synapses to HSP (Fig 1 and Fig EV1).

2. Many of the crucial conclusions are based on their elegant replacement strategy (KD of SP in combination with re-expression from a vector containing either a wild type or mutated 3' UTR, fig.6 and 8). However, this approach relies on the ectopic expression of SP, which could introduce artifacts. Therefore, it is necessary to have an independent approach that shows that endogenous miR-124 regulates endogenous SP during upscaling. In this respect, the most elegant approach would be to use a target protector LNA that prevents the endogenous miR-124 from binding to the endogenous SP, followed by SP immunostaining. This would provide conclusive evidence with regards to the functional importance of this miRNA-target interaction. Alternatively, this experiment could be performed with a miR-124 LNA, which however would not specifically address the association between miR-124 and SP. In any case, in my opinion it is essential to perform miR-124 loss-of-function approaches under basal and silenced condition in vitro and ex vivo to strengthen the results shown in Fig. 6 and 8.

To address reviewer's concern, we carried out a new experiment and test the ability of endogenous miR-124 to regulate endogenous SP expression during HSP. In order to prevent the targeting of endogenous SP by endogenous miR-124, we followed reviewer's suggestion and designed a miRCURY LNA miRNA Power Target Site Blocker (Qiagen, cat # 339199) that binds miR-124 target site on SP mRNA. This LNA was designed to outcompete miR-124 for binding SP mRNA. In comparison to using a LNA or sponge that directly inhibits miR-124, this strategy is expected not to affect other targets of miR-124 and therefore to be more specific of the SP interaction. Similar to our replacement strategy using SP-3'UTR-MUT, we found that transfecting cultured hippocampal neurons with 50 nM SP-LNA occluded the increase of the fraction of SP+ synapses as well that is normally induced by the TTX treatment (new Fig 6E-G). Overall, the fact that both deleting miR-124 binding region in SP-3'UTR and the SP-LNA approach gave similar results strongly support the role of endogenous miR-124 in the activity-dependent regulation of SP expression. We also found that transfecting neurons with SP-LNA is sufficient to promote synaptic recruitment of AMPARs and occludes HSP induced by TTX: this new data further suggests that endogenous interaction between miR-124 and SP mRNA controls non-uniform HSP.

We could not address the role of endogenous interactions between miR-124 and SP ex vivo as we found that single spine analysis of endogenous SP immunolabelling in organotypic slices is severely hampered by (1) the high synapse density in 3D tissue and (2) the difficulty of the antibody to penetrate >5 µm into the tissue, resulting in variable results depending on the z position of dendrites (see Figure 1 below).

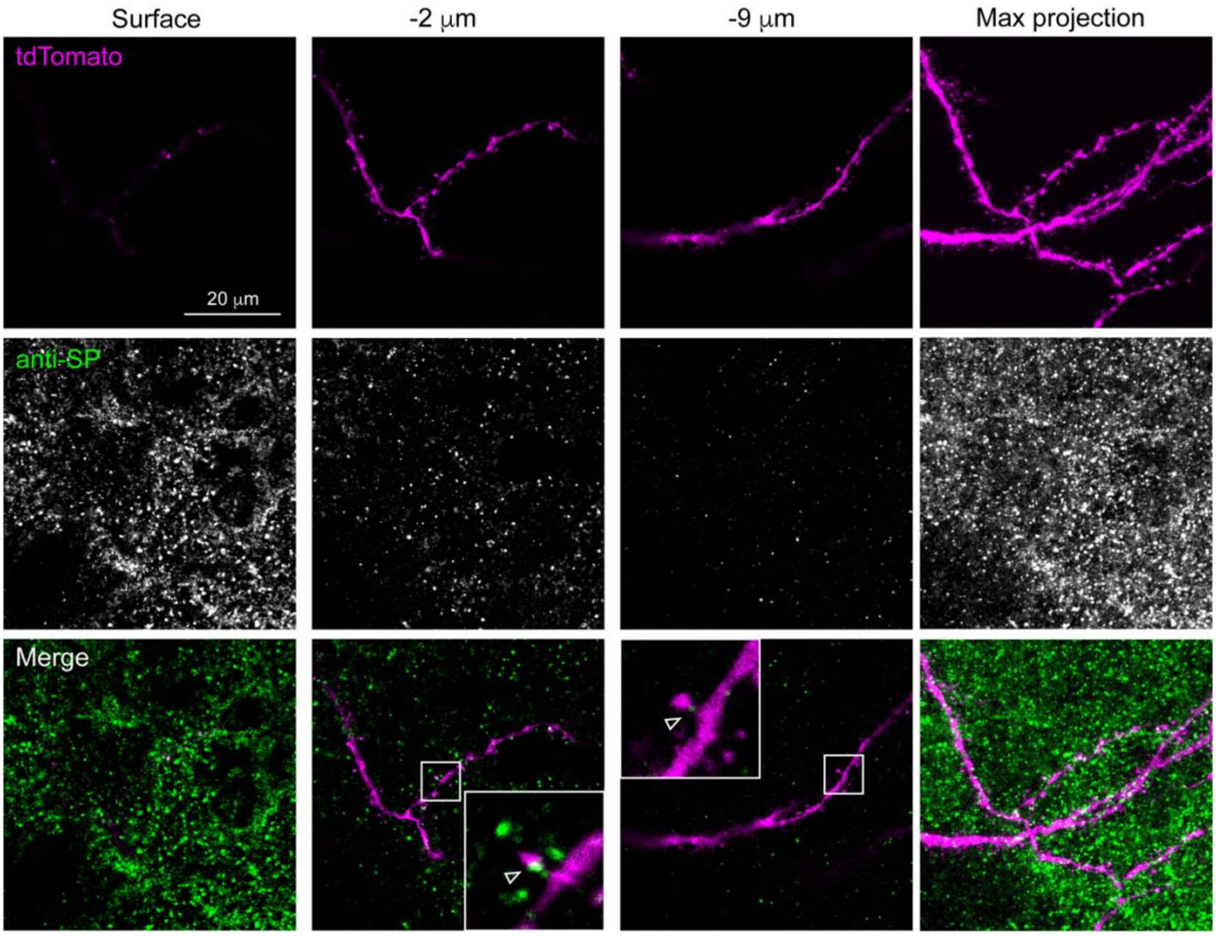

__Figure 1:__ **Immunolabelling of endogenous SP decreases with depth in organotypic slices.** Confocal images showing tdTomato fluorescent signal from electroporated CA1 pyramidal neuron (magenta) and anti-SP immunolabelling (green) at different z positions (surface, -2 µm and -9 µm) as well as in the z projection of maximum intensities. Note that the SP immunolabelling rapidly decreases with depth in the tissue.

3. Since the dendritic localization of miR-124 is somewhat controversial, showing convincing FISH data in dendrites of control- and TTX-treated neurons is imperative. However, the quality of the miR-124 FISH data (fig S7) provided does not allow to draw any conclusions, so this needs to be repeated. This analysis should be complemented by a confirmation of the dendritic localization of SP. Lastly if dendritic localization of miR-124/SP can be confirmed confirmed, then correlating the distribution of miR-124 to endogenous SP protein +/- TTX would be an interesting piece of data.

We agree with the reviewer that any evidence that both miR-124 and SP mRNA are present within dendrites is important to strengthen the idea that SP can be locally synthesized in dendrites to support miR-124 dependent HSP. In this respect, the presence of both miR-124

and SP mRNA within the synapto-dendritic compartment has been clearly reported previously, while we could not find in the literature any data supporting the opposite:

(1) The presence of miR-124 in the synapto-dendritic compartment has been evidenced in at least four previous reports from different labs **(Kye et al., RNA 2007; Lugli et al., J Neurochem 2008; Ho et al., Mol Cell Neurosci 2014; Siegel et al., Nat Cell Biol 2009)**, through direct visualization of miR-124 by FISH and/or RNA analysis of synaptosomal fractions by microarray **(Lugli et al., J Neurochem 2008)**, RT-qPCR **(Lugli et al., J Neurochem 2008; Ho et al., Mol Cell Neurosci 2014**) or Northern blotting **(Lugli et al., J Neurochem 2008; Siegel et al., Nat Cell Biol 2009)**. While these studies differ by the extent to which miR-124 is expressed in dendrites vs soma, we could not find any evidence in the literature that miR-124 is excluded from dendrites. We are thus puzzled that the reviewer finds the dendritic localization of miR-124 'somewhat controversial'.

(2) Similar to miR-124, SP mRNA was detected in the synapto-dendritic compartment through transcriptomic approaches (on microdissected neuropil or purified synaptosomes) in at least three studies **(Cajigas et al., Neuron 2012; De Solis et al., Front Mol Neurosci 2017; Hafner, Science 2019)**. Of note, the study by de Solis et al. suggests that SP mRNA increases in dendrites after eliciting LTP, consistent with the established role of SP in LTP.

In order to bring further confirmation to these previous reports, we made an attempt to refine/perform the detection of miR-124 and SP mRNA by FISH in mixed primary cultures. We now provide quantitative analysis of the signal obtained using our miR-124 probe (Appendix Fig S3). Our analysis reveals that the specific probe for miR-124 generates a much higher signal compared to the scrambled at both somatic and dendritic levels, strongly suggesting the presence of miR-124 in both compartments, although miR-124 seems to be much more abundant within the soma, consistent with its known role in the regulation of the expression of multiple transcription factors **(Sun et al., Front Cell Neurosci 2015)**. In light of these new quantitative analysis which comes in support of the previous studies listed above, we respectfully disagree with reviewer 1 about his/her view that "the quality of our FISH data does not allow to draw any conclusion".

Regarding the *in situ* detection of SP mRNA, we were unfortunately not able to set appropriate experimental conditions to obtain a specific signal with our probe (see Figure 2 below). Both scrambled and the SP mRNA probes generated similar signals not only in the cell body but also in neurites. However, our new puro-PLA data do confirm the presence of SP-mRNA in dendrites and further indicate that SP-mRNA is actively translated upon activity-deprivation. Overall, our model that local SP synthesis supports miR-124-dependent, non-uniform, HSP is strongly supported by the following observations:

(1) SP expression is required for HSP to occur (Fig 3, see also **Vlachos et al., PNAS 2013**). Furthermore, our experiments in which the endogenous miR-124 / SP-3'UTR interaction is inhibited indicates that HSP requires SP derepression by miR-124 (new Fig 6);

(2) SP translation in the synapto-dendritic compartment is increased upon TTX treatment and occurs preferentially at large synapses (new Fig 7); the presence of SP mRNA within dendrites is further supported by previous studies (see above);

(3) SP+ synapses are larger and preferentially contribute to miR-124-dependent HSP (Fig 1 and Fig EV1);

(4) The fact that miR-124 dependent HSP can be induced locally and occurs in a synapse autonomous manner (Fig 9 and Fig EV5) is best explained by SP local translation that occurs upon TTX treatment and which is compatible with the presence of both SP mRNA and miR-124 within dendrites.

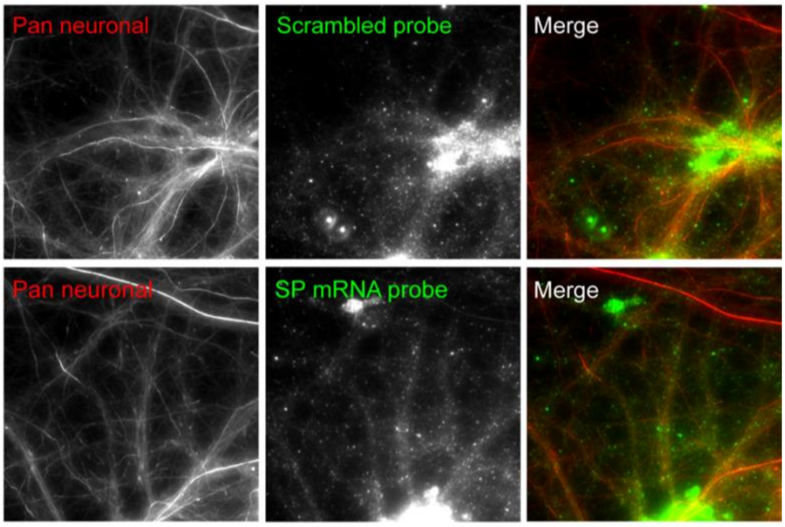

Figure 2: **FISH experiments to detect SP mRNA *in situ*.**

More minor points

4. Why were two different statistical methods used in fig. 3E and H? please explain.

The reviewer probably refers to data set from Fig 1E and Fig 1H. For these data sets, we indeed reported wrong statistical methods. The Kolmogorov-Smirnov test has now been used for both data sets. Statistics information has been corrected in the Fig 1 legend as follows:

(1) Fig 1E (distribution of AMPAR synaptic fluorescence intensities): UT-scaled vs TTX, ***P = 0.0005

(2) Fig 1H (distribution of mEPSC amplitudes): UT-scaled vs TTX, *P = 0.0316

5. An empty vector is not an appropriate control for the SH-RNA experiments performed in figure 3. This should be replaced with a vector expressing an shRNA of an unrelated (scrambled) sequence. The multiple rescue experiments are reassuring with regards to the specificity of the effects observed, but at least one experiment comparing the effect of the Sp-sh to a control vector expressing a scrambled Sh sequence should be performed.

To address this concern, we have repeated the experiment corresponding to initial Fig 3C,D and included a scrambled shRNA control, as requested (see also specific point #3 from referee #3). The scrambled shRNA did not affect the expression of endogenous SP compared to the empty vector condition. This is in contrast with SP-shRNA which induced a 40% decrease of SP expression as well as 35% decrease in the fraction of SP+ synapses, similar to our previous finding. Together with the fact that SP expression can be rescued by transfecting neurons with shRNA-resistant SP, this experiment thus validates the specificity of our knock-down approach. These results now appear in Fig 3C-E.

6. In Fig. 4, Sp regulation by endogenous miR-124 should also be validated in luciferase assays (TTX +/- pLNA)

The experiment suggested by the reviewer involves the exogenous expression of a luciferase construct fused to SP-3'UTR. It appears therefore redundant with our experiments in which endogenous SP is replaced by SP-3'UTR-WT or -MUT in cultured neurons (new Fig 6) or organotypic slices (new Fig 8). Moreover, given the requirement of an exogenous construct in which the 3'UTR can be directly mutated, neither the use of a luciferase reporter nor the use of SP-LNA are justified here. For these reasons, we decided, with all due respect for this suggestion, not to carry out this experiment.

7. In Fig. 5, It would be informative to know whether blocking miR-124 is sufficient to induce HSP at specific synapses (via induction of Sp expression)?

A similar experiment was already performed by Hou et al. **(Nat Comms, 2015)** in cultured hippocampal neurons and revealed that blocking miR-124 with siRNA induces synaptic AMPARs recruitment on its own. Our data in organotypic slices further reveal that mutating SP-3'UTR promotes non-uniform spine growth and leads to a partial occlusion of HSP, suggesting that impairing interactions between SP-mRNA and endogenous miR-124 is sufficient to induce HSP at specific synapses. Our new data in cultured neurons using SP TSB-LNA confirms this idea by revealing that blocking the targeting of endogenous SP by endogenous miR-124 (new Fig 6) is sufficient to both trigger SP expression at new synapses as well as AMPARs recruitment at synapses. Together with the fact that miR-124 overexpression inhibits HSP, these new results indicate that miR-124 downregulation upon activity blockade promotes HSP through SP derepression.

8. In their model, the authors postulate that the miR-124-Sp-mRNA complex is selectively active at large synapses. This in turn would invoke a selective recruitment of this complex. The authors should provide some discussion about possible mechanism(s) which could play a role in the selective recruitment of miRNA-target complexes to specific types of synapses.

The reviewer is asking an important, yet difficult question. Our model implies that the ability of individual synapses to undergo plasticity highly dependents on the presence of SP-mRNA and/or miR-124 as well as components of the translation machinery and of the miRISC complex. Multiple mechanisms might regulate the presence (or absence) of these various actors at proximity of synapses, including:

- The mRNA transport along dendrites and sorting to synapses which depend on 3'UTR and are regulated by activity **(Bauer et al., Nat Comms 2019; Buxbaum et al., Science 2014; Wang et al., Cell 2016; Cougot et al., J Neurosci 2008)**.
- The turnover of the miRISC complex, which depends on neuronal activity **(Banerjee et al., 2009)** and affects protein translation. In particular, a recent study revealed that homeostatic synaptic downscaling is regulated by a tripartite complex comprising translation regulators, the miRISC and 26s proteasome **(Srinivasan et al., PloS Biology 2021)**.

- The maturation of miRNAs through Dicer. A recent study demonstrated that single-synapse stimulation using glutamate uncaging promote the local maturation of miR-181a through Dicer activity **(Sambandan et al., Science 2017)**.
- Specific interactions of miRNAs with cognate mRNA targets which could protect miRNAs from degradation **(Pitchiaya et al., Cell Reports 2017)**.
- Storage in P-bodies whose dendritic location is regulated by neuronal activity **(Cougot et al., J Neurosci 2008)**.
- Interaction with natural circular RNAs serving as miRNA-sponges **(Hansen et al., Nature 2013; Piwecka et al., Science 2017).**

While all these mechanisms should be considered to investigate further the regulation of SP expression at specific synapses, we feel that they are too speculative at this stage. Instead, we propose to raise the question asked by the referee by adding the following sentence L507: "While our study suggests an important role of the miR-124/SP mRNA complex at proximity of synapses to mediate HSP, it remains to investigate the active mechanisms that (1) drive the localization of miR-124 and/or SP mRNA at specific synapses and (2) regulate interactions between miR-124/SP-mRNA in relation with the translation machinery and RISC.

Additional, non-essential suggestions:
the study would greatly benefit from life imaging, e.g assessing the accumulation of SP during TTX-induced upscaling at specific spines over time. However, this might be technically difficult over extended periods of time (e.g. 48 h) and therefore beyond the scope of the present study.

We thank the reviewer for this interesting suggestion. While SP time-lapse imaging would indeed provide very useful information about the time course of the homeostatic response and would allow us to perform pair-wise comparisons, such experiment in primary cultures is indeed highly challenging over extended periods of time and we feel that they are beyond the scope of the present study.

==

Referee #2:

In this paper, the authors propose an interesting role for miR-124 regulation of synaptopodin expression in homeostatic synaptic plasticity (HSP) and AMPAR recruitment to synapses. The work provides novel mechanistic insight into synaptic regulation during HSP, with the additional interest of a synapse-specific form of HSP. However, there are a number of issues that should be addressed prior to publication, as described below.

Fig 2:
I found it surprising that the authors used neurons at DIV10 for the experiments imaging surface GluA2 diffusion. The experiments presented in Fig 1, 3, etc used neurons at DIV15. More specifically, it is surprising that neurons at DIV10 have mature synapses and spines. Is this a characteristic of neurons cultured in BrainPhys? The authors should include a

statement justifying why immature neurons are used for this experiment, supporting evidence for SP expression, and (if the alternative culture medium is involved) an analysis of spine/synapse development in neurons cultured in this way.

For this particular experiment, we decided to transfect neurons at DIV8 and perform FRAP at DIV 10 for two main reasons:

(1) to increase the transfection efficiency as we have had a hard time to find RFP-SP and SEP-GluA2 co-expressed in the same neurons after a transfection at DIV10.

(2) we found that overexpressing for > 3 days SEP-GluA2 induced cytotoxicity and severely reduced the number of surviving cells. Of note, this was in contrast with expressing SEP-GluA2 fused to the 3'UTR (-WT or -MUT), possibly because of a better control of AMPARs expression by the neuron.

Although we did not perform a detailed comparative analysis of synaptic development in our cultures, the neurons that we imaged at DIV10 indeed displayed similar mature spine morphology compared to neurons at DIV14 (see Figure 3 below). The surprising maturity of synapses in our conditions can be explained by the fact that BrainPhys enhances synaptic maturation and network development in cultures obtained from rodent brains or derived from iPSCs **(Jackson et al., Exp Neurol 2018; Satir et al., Sci Rep 2020).** In particular, the study by Jackson et al. analyzed the expression of neuronal, glial and synaptic markers in mixed cultures at DIV10 and found a striking increase of the expression of NMDARs, NeuN and GFAP in neurons cultured in BrainPhys versus Neurobasal media. As for SP expression, it was previously reported to occur as early as from post-natal day 6 (P6) in hippocampal pyramidal cells and to be similar to the adult pattern from P9 **(Deller et al., 2000; Czarnecki et al., J Comp Neurol 2005; Bas Orth et al., 2005)**.

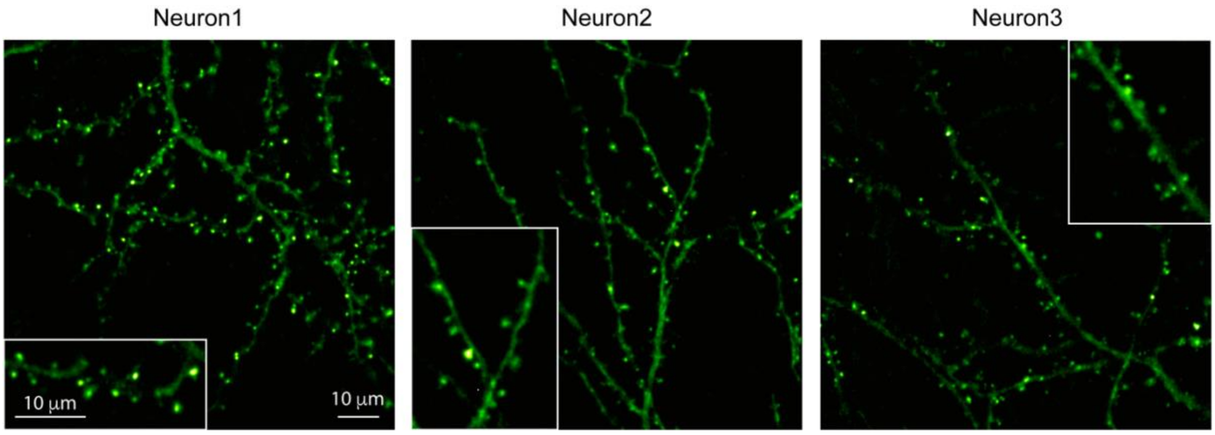

Figure 3: DIV10 neurons grown in BrainPhys display mature spine morphology. Epifluorescence images of neurons expressing SEP-GluA2 (in green). Note the presence of mature -mushroom-like- spines.

In order to address reviewer's concern, we made an attempt to perform experiments on DIV15 neurons that were co-transfected with SP-RFP and SEP-GluA2 at DIV10. Despite the low (co-)transfection efficiency, we could compare fluorescence recovery of SEP-GluA2 on SP+ spines (n = 20) vs SP- spines (n = 19) from 5 neurons. The results obtained are similar to those obtained in DIV10 neurons. In particular, the recovery was equivalent at both ages and the difference between SP+ and SP- spines was maintained (see Figure 4 below). While these

results strengthen our conclusion that SP+ synapses display higher ability to stabilize AMPARs, we feel that they are not solid enough to be part of the main manuscript.

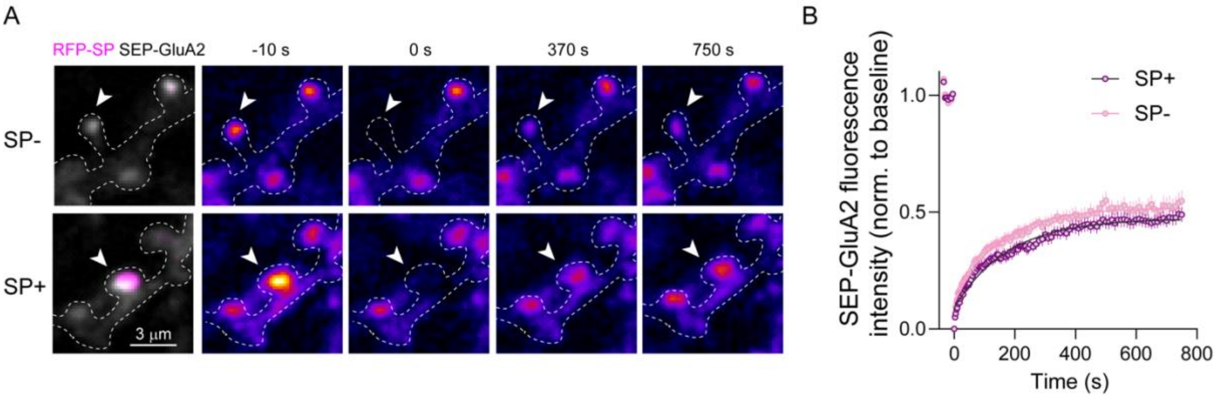

<u>Figure 4:</u> FRAP experiment in DIV15 neurons. (A) Example images of a FRAP experiment: SP- and SP+ spines from the same cultured hippocampal neuron transfected with SEP-GluA2 (gray and color-coded) + RFP-SP (red) 10s before, and 0, 370, 750 s after the photobleaching. (B) Quantification of FRAP dynamics for SP- vs SP+ spines. Recovery curves represent SEP-GluA2 fluorescence (SP+: n = 20 spines; SP-: n = 19 spines; N = 5 neurons from 1 experiment). The two traces were fitted using double exponential components equations and the convergence of the traces to a common fit was tested using the extra sum of squares F test. The F test indicates that the traces are best fitted by two divergent models (P < 0.0001).

Also, spine morphology is likely to be a critical determinant of AMPAR surface diffusion into or out of spines. The authors need to include a statement defining what morphological category of spines were analysed.

We selected spines emerging laterally from the shaft based on the SEP-GluA2 signal (green channel), without further discrimination. This is now indicated in the method section. Because the red channel was used to image recombinant RFP-SP, we could not use an appropriate cytosolic reporter for this experiment and were thus not able to perform spine morphometric analysis. However, our results revealed different AMPAR content for SP- versus SP+ spines while it is expected from the literature (and our slice data, see new Fig. 8) that SP- versus SP+ spines exhibit different sizes, with SP expression being predictive of more mature and larger spines **(e.g., Vlachos et al., J Neurosci 2009; Yap et al., eLife, 2020)**. Moreover, AMPAR content is known to be correlated with spine head volume **(Matsuzaki et al., Nat Neurosci 2001)**.

The bleach area/recording area is not well defined. Was the whole spine bleached and subsequently analysed? This must be defined more accurately.

We now detail further how we determined the bleach/recorded area, which corresponds to a square ROI of 10x10 pixels (1.8 x 1.8 μm) covering the entire spine but excluding the shaft.

Fig 5:
Some of these data, which appear to be fundamentally important to the conclusions of the study, are unconvincing. The authors state there is a "trend" in synaptic recruitment of AMPARs in response to TTX. A P-value of 0.47 is not even close to statistical significance. This is not a significant effect, and the effect of miR-124 is also non-significant. I acknowledge the mEPSC data and their statistical significance, so can the authors provide a strong explanation for the results in Figs 5c and d?

We agree that the results from the anti-GluA ICC and homer1c-GFP quantification on neurons expressing miR-Ctrl (Fig 5C,D) do not convincingly reproduce our results from Fig 1D. This is in contrast with the quantification of the percentage of SP+ synapses obtained from the same neurons (Fig. 5B) and also with the mEPSC data from sister cultures (Fig. 5F) which both indicate that neurons underwent HSP in response to TTX.

We can propose the two following explanations for these contrasting results:

(1) Our results in Fig. 1 and S3 suggest that SP+ /large/ strong synapses preferentially contribute to the increase in synaptic AMPARs. However, both SP- and SP+ synapses were included in the analysis of synaptic AMPARs or Homer1c signal in Fig.5. This analysis is therefore expected to be less sensitive to homeostatic variations.

(2) While values in Fig 5C,D display similar variance and increase upon TTX compared to Fig 1D and Fig EV1B, the sample size was lower while the number of groups to be compared was doubled (Fig 1D and S3: n = 26/33; Fig 5C,D: n=17/19), thus reducing the statistical power and the likelihood to detect significant statistical difference.

Fig.7:
In these, and other experiments in which the knockdown/rescue strategy is used, the SP present in neurons will be 30-40% RFP-SP with recombinant 3'UTRs, and 60-70% endogenous SP. Can this relatively small contribution of the recombinant gene account for all the effects seen in Figs 6, 7 and 8? This must be discussed.

We thank the reviewer for raising this important point. It should be noted that our replacement strategy aimed at expressing a recombinant RFP-SP construct (WT or MUT) that can be visualized while maintaining the global SP expression level at nearly endogenous levels (i.e., compatible with the expression of HSP).

The “effects” of the SP-3’UTR mutation described in Figs. 6, new Fig. 8 and new Fig. 9 are of two types:

(1) **<u>Increase in the fraction of SP+ spines:</u>** we only analyzed the behavior of exogenous RFP-tagged SP whose mutant (RFP-SP-MUT) escaped miR-124 regulation and was found expressed in a higher number of spines compared to the wild-type version. While endogenous SP was still expressed by 30-40% and remain under the control of miR-124, we could not analyze its distribution across spines in the same neurons as our antibody would also have labelled recombinant RFP-SP-MUT.

(2) **<u>Increase in spine size:</u>** the almost complete occlusion of the activity-deprivation effect that we found (Fig. 9H) despite the presence of endogenous SP can be explained by the fact that the SP-3’UTR mutation, promotes SP ‘overexpression’ rather than knock-down or loss-of-function of the protein. Indeed, while one can expect that the endogenous protein compensates for a mutation causing loss-of-function of the protein (i.e., SP not functional or not expressed), it is more difficult to envision how endogenous SP could compensate for a 3’UTR mutation causing an increased expression of the normal SP protein. It should be noted, however, that the effect we found on the fraction of SP+ spines is only partial in organotypic slices (Fig. 9G), and one should consider the relatively high variability of spine size values for the TetTx+ condition which makes uncertain the exact level of occlusion.

Finally, our new experiment in which we use a SP-LNA to directly target endogenous interactions between SP and miR-124 in cultured neurons gave similar results compared to our replacement strategy in cultured neurons, i.e., complete occlusion of the homeostatic response. Although we don't know to what extent the SP-LNA could protect endogenous SP mRNA from miR-124, this experiment further supports our model and suggest that at least a fraction of the SP-mRNA population is involved to support the homeostatic response.

I don't understand how the authors come to the conclusion, "This effect was accompanied by a selective increase of size for the largest spines while the smallest spines remained of similar size". This implies that spines were measured before transfection with the molecular replacement constructs, and then the same spines were analysed afterwards. How were the same spines identified several days after transfection? And how were spine sizes measured before transfection in the absence of a fluorescent marker? Maybe I'm missing something here, but this needs to be clarified.

We apologize for the lack of clarity and the confusing formulation. Because our experimental design does not allow us to visualize neurons morphology before expressing the SP-3'UTR mutant, we were not able to perform paired comparisons. Instead, we could analyze unpaired groups of neurons (expressing SP-WT or SP-MUT) and compare representative populations of spines corresponding to each group, as usually performed when pair-wise comparison is not possible (e.g., after chronic TTX treatment, **Turrigiano et al., 1998**). Importantly, expressing SP-MUT did not affect spine number (see Figure 5 below, suggesting that the two spine populations corresponding to the two conditions are equivalent: i.e., the increase in SP expression did not promote the formation (or pruning) of spines (see also **Okubo-Suzuki et al., Mol Cell Neurosci 2008**). Moreover, because our puro-PLA experiments as well as SP immunostainings indicate that SP synthesis occurs preferentially at large synapses and promote stabilization / potentiation (see also **Yap et al., eLife 2020**), the rank-ordering of spines should not be significantly altered by the mutation.

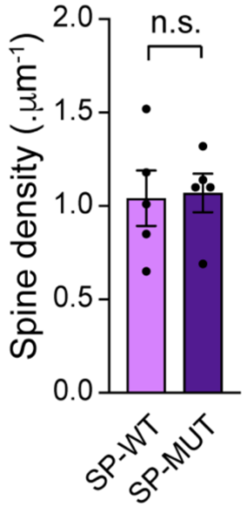

Figure 5: Expression of SP-3'UTR-MUT does affect spine density in CA3 pyramidal neurons from organotypic slices (n = 5 cells in each group from 3-4 different slice preparations). P = 0.84 (Mann-Whitney test).

To make our conclusion less confusing, we reformulated as follows: "In parallel, we found that neurons expressing SP-MUT displayed larger spines compared to neurons expressing SP-WT. Interestingly, the difference was greater when comparing the largest spines than when comparing the smallest spines (Fig 8E, F), suggesting a non-uniform regulation of

spines that was reminiscent of the non-uniform HSP observed in primary neurons upon TTX treatment”.

Fig. 8h: the authors refer to a partial occlusion of the effect of synaptic silencing by expressing the mutated SP-3'UTR. For this to be an acceptable conclusion, the difference between SP-WT and SP-MUT in the absence of TetTx must be statistically significant.

However, this essential comparison is not included on the graph. This must be rectified. Also, why are there such massive differences in sample size for the different conditions? The TetTX- conditions have n=220 and 616, while the TetTX+ conditions have n=25 and 26. I wonder if the authors were pushing for a statistically significant difference between SP-WT and SP-MUT in the absence of TetTX, hence they conducted hundreds of repeats. Perhaps if SP-MUT in the presence of TetTX had a comparable number of repeats, this difference might also be significantly different? This should be tested.

The massive differences in sample size for TetTx- vs TetTx+ conditions can be explained by the fact that ‘TetTx+ spines’ are determined through the apposition of presynaptic terminals labelled from a nearby pyramidal neurons, yielding very few contacts per neuron pair (ranging 1-7 in our data set). In contrast, TetTx- spines correspond to nearby spines from the same dendritic branch that are not contacted by a presynaptic terminal and are therefore more numerous. In order to address reviewer’s concern about the difference in sample size, we repeated the statistical comparison by taking a random selection of nearby TetTx- spines in order to match the sample sizes of the TetTx+ groups. This new comparison is now represented in Fig 9H. We also added the cumulative distribution of spine sizes in order to illustrate the non-multiplicative increase of spine size upon presynaptic silencing (Fig 9I).

Minor points:
Fig 4c:
Is the miR-124 binding site really in position 1574-1580 in both Gria2 and synaptopodin mRNA? If this is just a typo, it should be corrected.

This has been corrected for Gria2 for which the miR-124 binding is in position 187-194.

Fig. S7A,B:
The authors state, "staining was absent at both somatic and dendritic levels when using a control scramble probe, demonstrating the specificity of the signal." There is clearly a punctum present in control dendrites, hence this claim needs to be modified.

We now provide quantitative analysis of the staining in Appendix Fig S3 (see response to referee #1, major point #3) which shows that the signal generated by the scrambled probe is reduced by ~80% in comparison to the signal obtained with the miR-124 probe. We therefore removed the claim that the staining was absent when using the scrambled probe.

==

Referee #3:

The manuscript by Dubes et al. describes how mir-124-dependent expression of synaptopodin regulates input-specific homeostatic scaling of synapses in rat hippocampal

neurons. This is an important and elegantly designed study. Although overall data are solid and support the hypothesis, there are still some gaps which should be filled at the experimental or editorial level. I would recommend the paper for publication after thorough revision.

General points:

1) Some of Homer1c fusion constructs seem to have altered localization and no longer can be considered as specific postsynaptic marker: the construct seems to be all over the dendrites in some example images (e.g. Fig. 6A, Fig. S2). How was it even possible to calculate the homer1c-clusters in such a case? Although previous studies has not found any effect of Homer1c overexpression on PSD- and spine-size (e.g. Meyer, D., Bonhoeffer, T., and Scheuss, V. (2014). Neuron 82, 430-443), there might be other parameters affected, as overexpression of proteins always introduces a disturbance to cellular homeostasis, as well as levels of overexpression differing between different cells, this has the potential to introduce a lot of "noise" to the experiments. It is unclear why the authors chose to use overexpression as opposed to staining endogenous Homer1, since most of the data shown is from fixed cells where overexpression seems unnecessary for the assay setup.

We agree that staining of endogenous Homer1c or other post-synaptic scaffold would have been the best approach. However, the use of multiple antibodies was not always possible, in particular in Fig. 1, where both endogenous SP and AMPAs were stained with rabbit and mouse antibodies, respectively, and did not leave room for immunostaining endogenous scaffolds with our available antibodies. To bring more flexibility in the choice of the antibodies across the study, we therefore opted for the expression of recombinant Homer1c-GFP/-dsRed/-BFP constructs that we and others extensively used in various experimental conditions with no obvious side effects on synaptic structure or function **(e.g., Letellier et al., Nat Neurosci 2014; Letellier et al., Nat Comms 2018; Mondin et al., J Neurosci 2011; Gianonne et al., Cell Reports 2013; Charrier et al., Cell 2012; Meyer et al., Neuron 2014; Catsburg et al., eLife 2022)**. In support of this observation, it should be noted that the AMPARs-mediated mEPSCs recordings in Fig. 1F-H and Fig EV2 were performed from untransfected neurons and are comparable to those recorded from neurons transfected with Homer1c-GFP and miR-Ctrl or miR-124 in Fig 5E,F. We kept Homer1c-GFP/-dsRed/-BFP as a postsynaptic marker throughout the study for consistency in the definition of synapses. While showing a diffuse signal in some neurons as pointed out by the reviewer, we could nonetheless easily segment Homer1c-GFP/dsRed/-BFP clusters using the Multidimensional Image Analysis Software ran in Metamorph (see also our detailed response below).

2) It is not described clearly in the methods & protocols how the intensity measurements of synapse-components (SP, AMPAR) were performed. For example, it is unclear whether the intensity was measured in a single plane, or whether the authors recorded z-stacks to image the entire dendritic volume - the latter would yield way more precise results, as imaging a single z-plane entails the risk of under-estimating fluorescence intensity if the signal is slightly out of focus, as well as introducing an additional level of error.

The intensity measurements of synaptic components were made from epifluorescence images acquired with an inverted microscope (Nikon Ti-E-Eclipse) equipped with a CMOS Prime 95B Scientific camera (Photometrics), as indicated in the method section. We have

shown that this method is appropriate to image and perform quantitative analysis of synaptic parameters in primary cultures, which largely organize in 2D **(Letellier et al., Nat Neurosci 2014; Letellier et al., Nat Comms 2018; Chamma et al., Nat Comms 2016)**. The fast camera-based acquisition of the signal allowed us to image a higher number of neurons per condition and per session compared to slow z-stacks acquisition through scanning confocal microscopy. This allowed us to minimize the cell-to-cell variability that is inherent to dissociated neurons. On another hand, because epifluorescence microscopy collects part of the signal coming from out-of-focus planes, the error arising if the imaging plane is slightly out of focus should be lower compared to single plane confocal microscopy.

Further, it says in line 732 that "we estimated the percentage of Homer1c-GFP clusters for which at least 20 % of pixels was also positive for SP; these clusters were considered as SP+ synapses". What does it mean "we estimated"? How was it estimated? Please describe your data analysis workflow in more detail. It would help to include an example image of the analysis workflow where the detected homer-clusters are outlined.

We apologize for the lack of clarity regarding our data analysis and we now provide further details in the method section. The automated measurement of synaptic parameters in cultured neurons was performed using routines made in the Metamorph software and involving the following steps (see Figure 6 below):

(1) **Definition of the dendritic outline** by thresholding the Homer-1c signal.

(2) **Definition of synaptic ROIs:** unbiased binary segmentation of Homer1c-GFP/-dsRed clusters was obtained through wavelet decomposition of the signal. This was performed by using the Multidimensional Image Analysis Software ran in Metamorph (https://www.yumpu.com/en/document/read/14877227/mia-040-tutorial), as in our previous studies **(e.g., Letellier et al., Nat Neurosci 2014; Letellier et al., Nat Comms 2018)**.

(3) **Measure of integrated Homer1c-GFP/-dsRed/-BFP intensity** within synaptic ROIs as a proxy for synapse size.

(4) **Measure of AMPARs immunosignal within synaptic ROIs**. Synaptic ROIs were transferred from the Homer1c image to the GluA image and the average GluA intensity was measured for each ROI.

(5) **Quantification of SP+ synapses**. Synaptic ROIs were transferred from the Homer1c image to the SP image and the SP signal was thresholded. For each synaptic ROI, we measured the fraction of pixels that overlapped with SP thresholded signal. Synapses were considered 'SP+' when at least 20% of their area overlapped with SP thresholded signal.

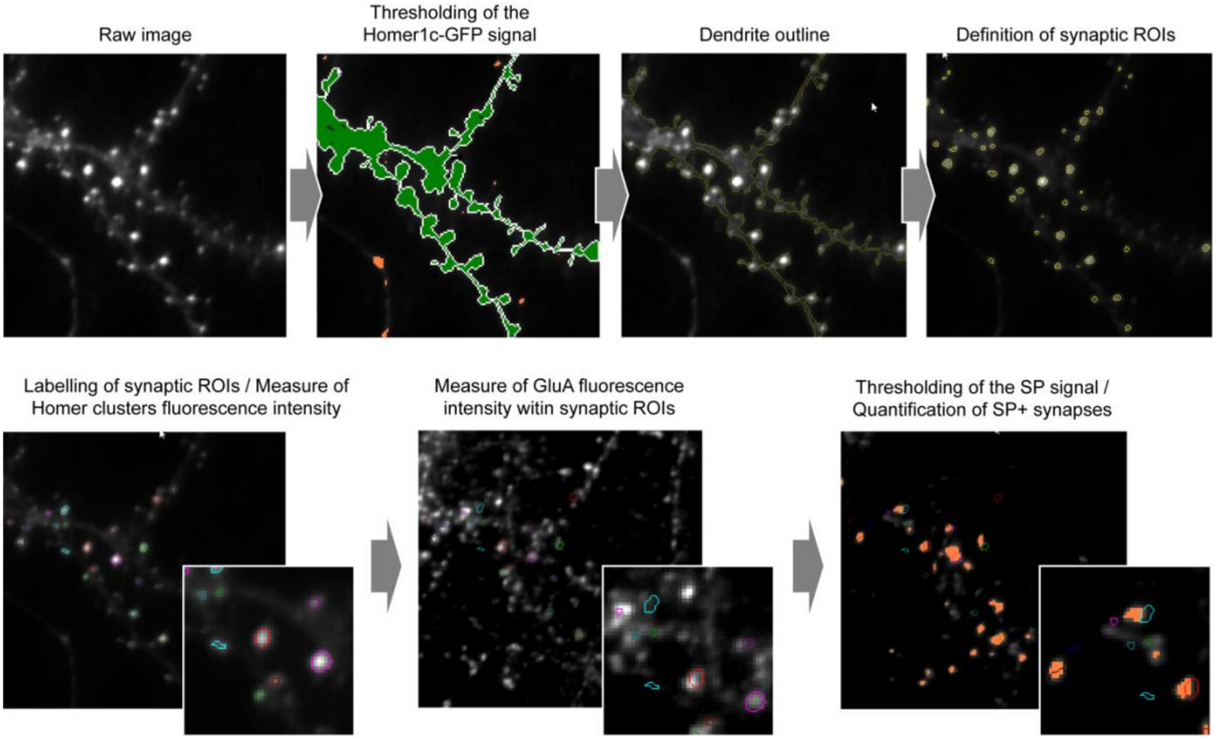

Figure 6: Flowchart illustrating synaptic parameter measurements through image analysis with Metamorph software

Also, the way the workflow is described in methods & protocols, it seems the authors did not discriminate at all between excitatory shaft-synapses and spine-synapses for any of the analyses? If so, this should be clearly stated in the text, since there are clear differences in functionality between those two types of synapses, and this has implications for the interpretation of the data.

Shaft and spine-synapses were indeed not discriminated in culture preparations as it would be very complex to implement in our automated synaptic analysis. In particular, in several of the experiments (including Fig 1), we were missing a volume marker that is necessary to clearly define spines versus shaft at the morphological level. This is now clearly stated in the method section.

3) Sometimes n-numbers in graphs represent cells (e.g. Fig. S6), sometimes individual spines (e.g. Fig. S8), i.e. the n-number is very high, making even small changes "statistically significant". Would it make more sense to average all results per cell, to make the analysis more robust? If not, please argue why it would make sense to regard a single spine as "one n" in some experiments and not in others.

For slices data in Figure 9: average results per cell would not be relevant in CA3 pairs because of the too low number of contacts per pair. In most data from dissociated neurons, n-numbers represent individual cells in which a population of synapses was sampled to

compute a representative average synaptic value. In graphs where different categories of synapses were compared (SP+ vs SP-, small vs large), n instead represents the number of individual synapses pooled from different cells. In this latter case, an equivalent number of synapses was sampled for each cell.

Also, please indicate the number of independent experiments / neuronal preparations for the individual experiments. In methods & protocols it is merely stated "Data are presented as mean {plus minus} SEM of three or more experiments performed in independent preparations". Please indicate the exact number for each experiment in the figure legends.

We now indicate the exact number of experiments/preparations in the figure legends.

Specific points:

1. Figure 1 and corresponding results: Fig. 1A: Please put the corresponding channel labels (SP, GluA, Merge..) also into the images under the TTX heading to make it easier to grasp everything at a glance. The "untreated" example of synaptopodin staining does not really represent the average 25% of SP+ spines shown in 1B; either the contrast in the SP panel is too low, or it is a bad example.

We have added channel labels in the images of both conditions for better clarity. We have also changed example images and/or color channels for more representative ones.

Line 134 / Fig. 1E: "increase in AMPAR synaptic content observed for TTX-treated neurons compared to untreated ones was not multiplicative and selectively occurred at synapses with the highest AMPAR content" -> it is not immediately clear how the cumulative distribution and the scaling factor shown in Fig. 1E prove this statement. The meaning of the scaling factor or how it was obtained is not explained at all the first time it is introduced (Fig. 1E), but only later for Fig. 1H. Please include one or two explanatory sentences on how the data was analyzed and interpreted.

We have modified the sentence as follows: ‘To investigate whether the increase in synaptic AMPAR abundance (Fig 1D) was multiplicative, we scaled synaptic AMPAR fluorescence intensities from control cells by the same factor (1.26) in order to match the average synaptic AMPAR fluorescence intensity from TTX-treated cells. We next compared the cumulative distributions of AMPAR fluorescence intensities from scaled control and TTX-treated cells and found a significant difference, indicating that HSP was not multiplicative and selectively occurred at synapses with the highest AMPAR content (Fig 1E).’

Line 156: "Consistent with this finding, the rank-ordered mEPSC amplitudes from TTX-treated cells plotted against rank-ordered mEPSC amplitudes from control cells were better fitted with a quadratic function than with a linear function (Fig. S4F)": This is unnecessary statement, because every dataset will be better fitted with a quadratic function than a linear function, because there is simply an additional variable in a quadratic function.

We thank the reviewer for pointing this out. We replaced this statement by the following sentence: ‘Finally, the rank-ordered mEPSC amplitudes from TTX-treated cells plotted

against rank-ordered mEPSC amplitudes from control cells revealed that the scaling factor was not uniform, i.e. being close to one for small amplitudes and increasing for larger amplitudes (Fig. EV2F).'

2. Figure 2 and corresponding results: The RFP-synaptopodin construct used in Fig. 2, as well as similar constructs used in literature cited by the authors (e.g. Vlachos et al, 2009; Konietzny et al, 2019), does not contain the 3'UTR of the endogenous synaptopodin, which the authors show later is important for the regulation of local synaptopodin translation. Can the authors speculate on how the regulation of such an overexpression constructs, which seems to behave very similarly to the endogenous protein in terms of localization and abundance in spines, could be mediated despite the lack of the 3'UTR regulatory element? (The authors later show that overexpressed SP-RFP dos not show the same increase upon TTX treatment as endogenous SP)

We thank the reviewer for raising this important point. Although the absence of 3'UTR regulatory sequence results in more than a twofold increase in the number of SP+ synapses, the overall synaptic SP localization and abundance seem largely unaffected **(e.g., Vlachos et al., J Neurosci 2009; Konietzny et al., J Cell Sci 2019; Wang et al. Plos One 2016)**. The fact that the deletion of the miR-124 3'UTR binding region in SP 3'UTR is not sufficient to allow SP expression in every single spine suggests the existence of additional limiting factors that precludes SP expression at synapses. In particular, SP mRNA and the translation machinery might not be available at all synapses. Moreover, recombinant SP mRNA lacking the complete 3'UTR regulatory element might not be targeted/stabilized correctly to synapses **(see for instance Bauer et al., Nat Comms, 2019)**, despite being overexpressed and not regulated by miR-124.

In line 176 the authors conclude "SP promotes the synaptic stabilization of surface diffusing AMPARs". This conclusion is too specific, as (they later mention this in the discussion), the presence of synaptopodin, indicative of a spine apparatus, indiscriminately inhibits the diffusion of (trans)membrane proteins with a large intracellular domain (Wang et al, 2016a). The causal connection between the presence of SP and AMPAR anchoring is not shown, this is only a positive correlation. Therefore, there is no basis for the statement directly after this sentence.

We agree with the reviewer that this statement is too specific and not accurate after this sentence. We have modified it for: 'showing greater ability of SP+ synapses to stabilize surface diffusing AMPARs in comparison to SP- synapses (Fig 2C,D). Together, these observations suggest that the recruitment of SP at a subset of synapses upon TTX treatment (Fig 1A,B) is associated with the stabilization of surface-diffusing AMPARs during HSP (Fig 1C-E).'

3. Figure 3, S3 and corresponding results: shRNA knock down experiments - it would be good practice to use another shRNA as control, scrambled or against an exogenous gene, instead of an empty vector.

To address this concern, we have repeated the experiment corresponding to Fig 3C,D and included a scrambled shRNA control, as requested (see also minor point #5 from referee #1). The scrambled shRNA did not affect the expression of endogenous SP compared to the

empty vector condition. This is in contrast with our SP-shRNA which induces a 40% decrease of SP expression, similar to our previous finding. Together with the fact that SP expression can be rescued by transfecting neurons with resistant SP, this experiment gives strong support to the specificity of our knock-down approach. These results now appear in Fig 3C-E.

Line 192: "knocking-down endogenous SP in cultured neurons did not alter the basal synaptic accumulation of AMPARs": How do the authors reconcile this? According to Fig 1C, reducing SP should lead to fewer SP+, less AMPAR anchoring and therefore lower overall AMPAR-density.

Our results recapitulate previous findings using similar knock-down approach **(Vlachos et al., J Neurosci 2009)** or constitutive SP knock-out model **(Vlachos et al., PNAS, 2013; Zhang et al., J Neurosci 2013; Korkotian et al., J Neurosci 2014; Yap et al., eLife 2020)**. Overall, there is strong evidence that spine size and basal synaptic transmission are not affected by SP KD or KO. In contrast, activity-dependent synaptic potentiation (LTP or HSP) is impaired. Although the reason why SP+ spines contain more AMPARs compared to SP- spines remains to be fully addressed, the fact that SP is required for synaptic potentiation but not basal transmission suggests a role for SP in the induction of synaptic potentiation rather than its maintenance of synaptic potentiation. As such, SP might not be directly involved in the anchoring AMPARs at the synapse but more likely behaves as a 'tag' that enables synapses to potentiate (e.g., thanks to the close proximity of Ca^{2+} stores and/or through promoting actin polymerization, see **Vlachos et al., J Neurosci 2009**).

Line 193: "... these results indicate that the presence of SP is required for synapses to undergo HSP": This statement needs further validation. A 40% reduction of SP does not mean its absence of all synapses. To make this statement, the authors need to show the SP- and SP+ distribution, as in Fig1, upon shRNA knockdown of SP and show that only the SP+ synapses undergo HSP.

We now provide in Fig 3C-E a more detailed analysis of the effect of SP knock-down. Our results indicate that expression of SP-shRNA but not scramble shRNA results in fewer SP+ spines compared to the empty vector condition. This is in agreement with the study of **Vlachos et al. (J Neurosci 2009)** which also reports a complete blockade of chemical LTP upon SP knock-down, despite the persistence of SP+ spines. Unfortunately, because the use of a GFP reporter for SP-shRNA did not leave room for immunostaining of both endogenous AMPARs and SP, we could not compare the expression of AMPARs at SP+ vs SP- synapses upon SP knock-down.

FigS3C: Is the y-axis integrated or averaged intensity?

The y-axis represents average intensity. This is now clearly indicated in Fig. S3C.

4. Figure 4 and corresponding results: Fig. 4C: Exactly the same experiment + results was shown in Elramah et al. 2017, so it was good that the authors reproduced those results, but this paper should be cited at this point. Also, double-check the sequences, since Elramah et al. indicate the complementarity between miR-124 and SP 3'UTR at position 1614-1620, while the authors here indicate 1574-1580. Where does this discrepancy come from?

The difference in the position of the miR-124 seed region in the SP 3’UTR comes from the fact that experiments in Elramah et al. were performed on mouse neurons while experiments in the present study were performed in rat neurons. Indeed, the length and exact composition of SP 3’UTR between these two species diverge but the presence of a miR-124 site remains. Interestingly, in Human SP 3’UTR, a miR-124 is also present at positions 1754-1760 raising the possibility that this mechanism operates in the human brain too.

5. Figure 7+8 and corresponding results: For experiments in CA3 neurons it should be stated which dendrites were selected for analysis (basal, apical, oblique...). For instance, thorny excrescences contacted by mossy fibers have very different morphology. They are very big, all of them are positive for synaptopodin and they undergo very different type of plasticity. It would be very interesting to look at these spines or to make sure that they were not included in the analysis.

Thorny excrescences found on the main (primary) apical dendrite as well as distal spines on tuft dendrites were excluded from the analysis in Figures 7 and 8 (new Figures 8 and 9). We only considered spines from apical or basal oblique dendrites (secondary/tertiary) which correspond to recurrent synapses made with other CA3 pyramidal cells. This is now clearly stated in the Method section.

6. Discussion:
Line 372: "However, we report.... synaptic weight distribution": The authors do not track the fates of individual synapses as TTX is applied. The data is solid, but e.g. whether the smallest SP- synapses indeed remain as such and the other slightly larger SP- ones become SP+ is not shown. One could also see the data such that, if one puts the threshold of SP-/SP+ a little lower, that ONLY SP+ synapses are linearly scaled. The manuscript would benefit strongly from such a tracking of fates, but this experiment is not necessarily required.

The reviewer raises an interesting point. Indeed, the increase in the percentage of SP+ spines upon TTX treatment suggests that a fraction of SP- spines (most likely associated with the miR-124/SP-mRNA complex) become SP+. While tracking the fate of individual synapses would allow us to directly address that point, this experiment appears technically challenging to perform over a 48h period in cultured neurons and we feel that this is beyond the scope of our study. It should be noted, however, that the new data from SP puro PLA which allowed us to capture the translation process itself upon treating neurons with TTX revealed a discrete distribution of translation sites across dendrites, and a higher proximity with large synapses. These data further support the non-uniform regulation of synapses during HSP.

Minor points:

Line 23: "to a same" -> "to the same"

Corrected

line 40: "synapses" plural should be synapse singular, "with a same gain" -> "with the same gain"

Corrected

line 92: "interesting" -> "interestingly"

Corrected

Line 359 "not only we demonstrate" - "not only do we demonstrate"

Corrected

Line 317 "apposed" -> "opposed"

Corrected

In Fig. 1, 3, 5, and 6, the label size inside the microscopy images is too small; it is hard to read. This is partly due to the poor quality of the exported pdf, try to enhance the quality / reduce compression. The colour of the blue channel is hard to make out against the black background. Maybe choose a lighter blue that contrasts better with the black background.

We have increased the size of labels and positioned them on the side of microscopy images for clarity. We have also changed the color-coding for example images in Fig 1 and Fig 6 in order to improve the contrast.

FigS2: It is unclear with which AB this staining was performed. Additionally, no information can be found on the Agrobio AB against GluA1.

The antibody used against GluA1 is a gift from D. Choquet (IINS; Bordeaux). It has been customized by Agrobio and characterized in a number of previous studies **(Nair et al., J Neurosci 2013; Penn et al., Nature 2014; Letellier et al., Nat Neurosci 2014, Letellier et al., Nat Comms 2018; Goncalves et al., PNAS 2020)**. The immunogene used to generate the antibody was RTSDSRDHTRVDWKRC within the extracellular region of the GluA1 subunit **(Nair et al., J Neurosci 2013)**. This information has been added to the Method section.

Fig. S8A: the white arrowhead indicates two different synaptic terminals in the different panels

This has been corrected.

For all of the overexpression plasmids used, it would be nice to also specify the respective promoters in the Methods & Protocols. Were all those expressed under the CMV promoter, synapsin, beta-actin or a different one?

This is now specified in the Methods and Protocols section.

1st Revision - Editorial Decision **27th Apr 2022**

Dear Mathieu,

Thank you for submitting your revised manuscript to The EMBO Journal. Your revision has now been seen by the referees and their comments are provided below. Referees #1 and 2 have a few more queries that I would like to ask you to respond to in a final round of revision. I think it would be helpful to discuss your response to referee #2's comments.

When you submit the revised version would you also please take care of the following editorial issues:

We can only have 3-5 keywords.

Our COI section is called "Disclosure statement and competing interests". Please also see guide to authors.

Please check figure callouts to: Fig 7A,B+G, Figs 10, EV4, S2 & S3, Fig EV4 panel Appendix Fig. S1A,B,D&G

Appendix Fig S3 callout is missing the 'S'.

The appendix is missing a ToC and the appendix figures should be part of the Appendix and uploaded as one file. Please make sure that the figure legends to the appendix figures are in the appendix file.

I see that you have included a reagent table. You can either turn it into a formal Reagents and Tools table - see guide for authors - or include it in the appendix file.

"Methods and Protocols" needs correcting to "Materials and Methods". It should also follow immediately after the Discussion section.

Please add "Expanded View Figure Legends" heading.

We encourage the publication of source data, particularly for electrophoretic gels, blots and data plots with the aim of making primary data more accessible and transparent to the reader. Would you be willing to provide a PDF file per figure that contains the original, uncropped and unprocessed scans of all or key gels used in the figure? The PDF files should be labelled with the appropriate figure/panel number, and should have molecular weight markers; further annotation could be useful but is not essential. The PDF files will be published online with the article as supplementary "Source Data" files.

We include a synopsis of the paper (see http://emboj.embopress.org/). Please provide me with a general summary statement and 3-5 bullet points that capture the key findings of the paper.

We also need a summary figure for the synopsis. The size should be 550 wide by [200-400] high (pixels). You can also use something from the figures if that is easier.

Our publisher has also done their pre-publication check on your manuscript. When you log into the manuscript submission system you will see the file "Data Edited Manuscript file". Please take a look at the word file and the comments regarding the figure legends and respond to the issues.

That should be all. Let me know if you have any further questions.

Best Karin

Karin Dumstrei, PhD
Senior Editor
The EMBO Journal

Instructions for preparing your revised manuscript:

Guide For Authors: https://www.embopress.org/page/journal/14602075/authorguide

Use the link below to submit your revision:

https://emboj.msubmit.net/cgi-bin/main.plex

Referee #1:

The authors addressed thoroughly and elegantly the crucial major points of the revision. They provide further evidences for a role of miR-124-dependent local translation of synaptopodin in homeostatic upscaling of excitatory synapses. This represents a significant advance in our understanding of the mechanisms driving homeostatic plasticity and will be of great interest for the neuroscience community.
Before publication the authors should deal with one last issue regarding their data analysis. For the statistical analysis they pooled individual cells from different biological replicates followed by Anova and post hoc t-tests. As recently pointed out by Yu et al. (Neuron 2022, https://doi.org/10.1016/j.neuron.2021.10.030), this is based on the incorrect assumption that these are fully independent data points. The authors need to take into account the correlation between the data points in their statistical analysis by either using a linear mixed effect model or by aggregating the cells arising from the same biological preparation. This point is especially important in all the experiments where a high number of cells has been pooled, for example in fig. 1b,d and g. Pending the refinement of the statistical analysis, I fully support the publication of the manuscript.

Referee #2:

In this revised manuscript, the authors have made several improvements, and addressed many of my previous criticisms.
I am still concerned about the data presented in figure 5C/D, and the conclusions drawn from them. I acknowledge that it makes sense for there to be an increase in synaptic AMPARs and Homer with TTX, given the increase in mEPSC amplitude, however it is still inappropriate to refer to any effect here. The spread of the data points clearly shows there is no detectable effect of TTX, nor of miR-124, on synaptic AMPARs. The authors offer some explanations for the discrepancy, the first of which they should test experimentally. I.e., how does the analysis look when SP- and SP+ synapses are considered separately? I don't agree with the authors' second point at all.

Referee #3:

In the revised version of the manuscript by Dubes and colleagues, the authors convincingly addressed all my points. They included additional experiments and controls, explained analysis pipeline and improved manuscript flow and conclusions. This is a very nice work and I would recommended it to be accepted. There is only minor thing to be improved. Although the number of spines, cell and independent experiments have been added in the main figures, it is still missing in some panels in EV1. Please include it.

Referee #1:

The authors addressed thoroughly and elegantly the crucial major points of the revision. They provide further evidences for a role of miR-124-dependent local translation of synaptopodin in homeostatic upscaling of excitatory synapses. This represents a significant advance in our understanding of the mechanisms driving homeostatic plasticity and will be of great interest for the neuroscience community.
Before publication the authors should deal with one last issue regarding their data analysis. For the statistical analysis they pooled individual cells from different biological replicates followed by Anova and post hoc t-tests. As recently pointed out by Yu et al. (Neuron 2022, [https://doi.org/10.1016/j.neuron.2021.10.030 | https://doi.org/10.1016/j.neuron.2021.10.030]), this is based on the incorrect assumption that these are fully independent data points. The authors need to take into account the correlation between the data points in their statistical analysis by either using a linear mixed effect model or by aggregating the cells arising from the same biological preparation. This point is especially important in all the experiments where a high number of cells has been pooled, for example in fig. 1b,d and g. Pending the refinement of the statistical analysis, I fully support the publication of the manuscript.

We thank the referee for his/her the nice comments. To address the concern about the fact that data points from the same biological replicate (cell culture or independent experiment) might be correlated, we ran new statistical analysis for data sets corresponding to Fig. 1B, D and G as suggested by the reviewer. However, because several of our data sets (Fig. 1B and 1D) were obtained by < 5 biological replicates, we could not confidently apply mixed-effects models (Harrison et al., 2018). We instead used a multi-linear regression model and applied a fixed effect to the biological replicate (experience) factor. The new analysis (which is detailed below) confirms the effect of the TTX treatment on the percentage of SP+ synapses, the average intensity of synaptic AMPARs immunosignal as well as on mEPSCs amplitudes.

- **Figure 1B: Percentage of SP+ synapses for untreated (UT) and TTX-treated neurons**

The number of experiments (cultures) is limited and equal to two (see below).

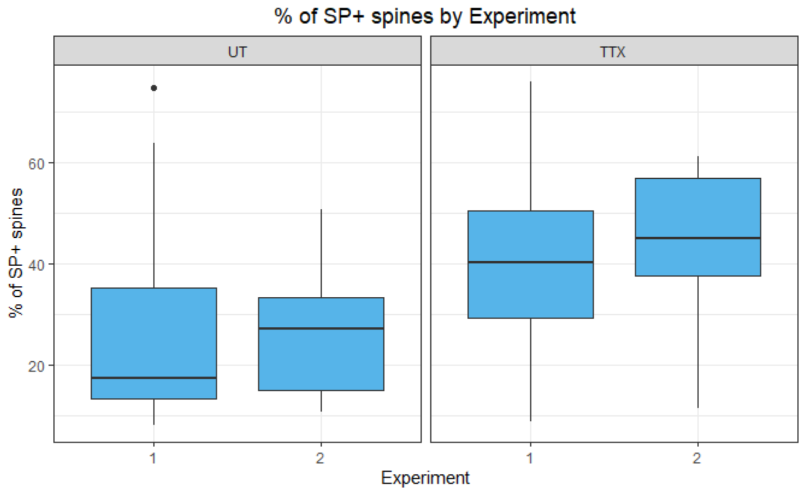

For this reason, a multi-linear regression has been chosen to adjust the analysis on the experience factor with an indicator parameter (fixed effect); because random effects cannot be applied when the groups are inferior than 5 (Harrison et al., 2018). The results of the

multi-linear regression show a significant difference between the percentage of the SP+ spines in untreated (UT) *versus* TTX-treated condition (regression coefficient = 13.482, p-value = 0.00385 **, see below). Since the regression coefficient is positive, that indicates that the mean percentage of SP-positive spines is higher for TTX-treated than untreated condition (UT).

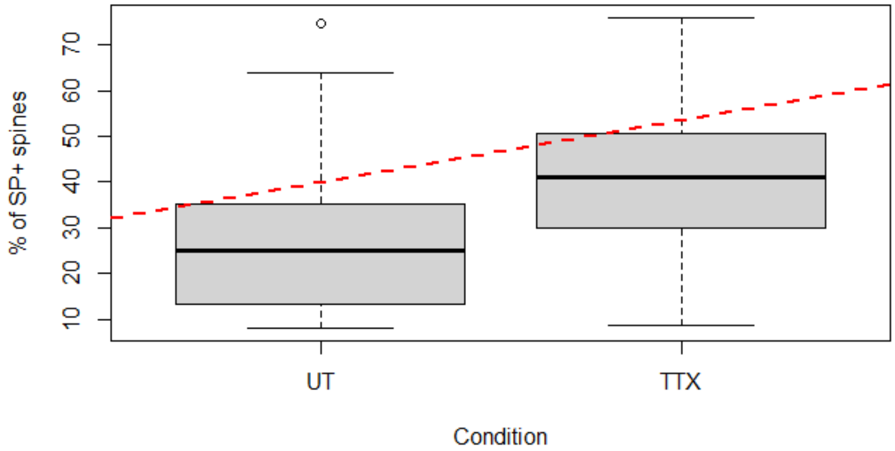

Additionally, we can reasonably accept the goodness of fit of the model after the visualization of the residual distribution. The histogram of the residuals is closed to a normal distribution and the QQ plot tends to a line (see below).

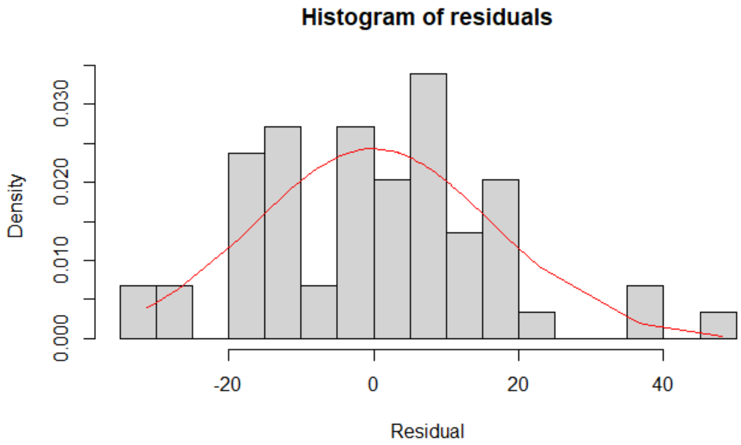

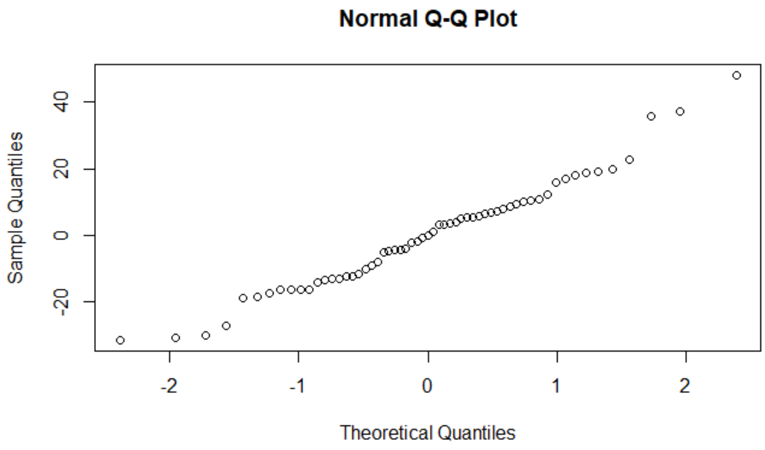

• **Figure 1D: Average AMPAR synaptic fluorescence intensity in UT or TTX-treated neurons**
The number of experiments (cultures) is limited and equal to two (see below).

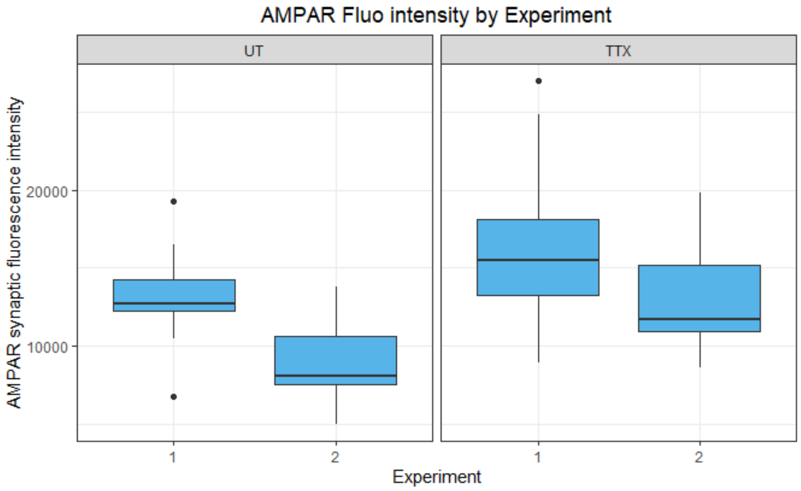

As previously, a multi-linear regression has been chosen to adjust the analysis on the experience factor with an indicator parameter (fixed effect). The results of the multi-linear regression show a significant difference between control (UT) *versus* TTX-treated condition (regression coefficient = 3243.9, p-value = 0.001320 **, see below). Since the regression coefficient is positive, this indicates that the mean of AMPAR synaptic fluorescence intensity is higher for TTX-treated than untreated condition (UT).

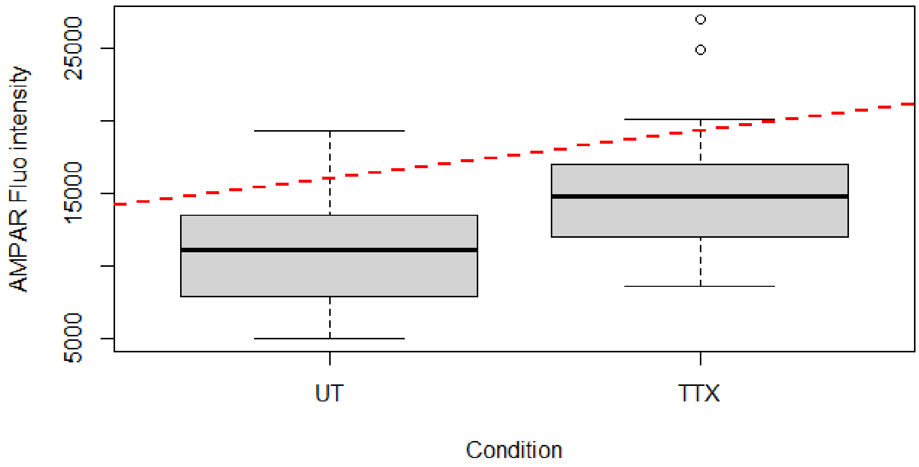

Additionally, we can reasonably accept the goodness of fit of the model after the visualization of the residual distribution. The histogram of the residuals is closed to a normal distribution and the QQ plot tends to a line (see below).

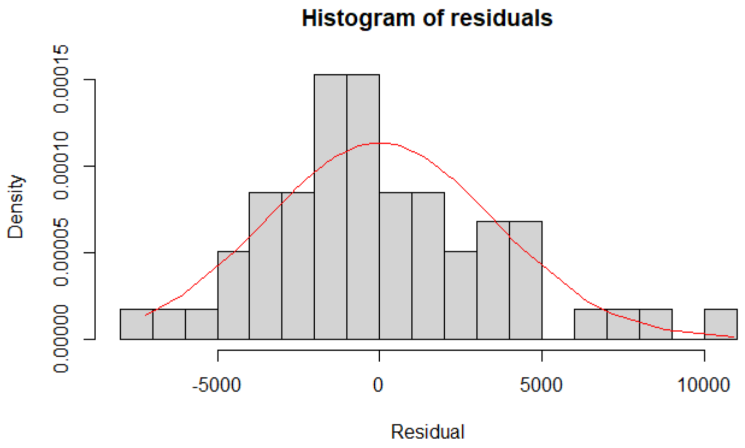

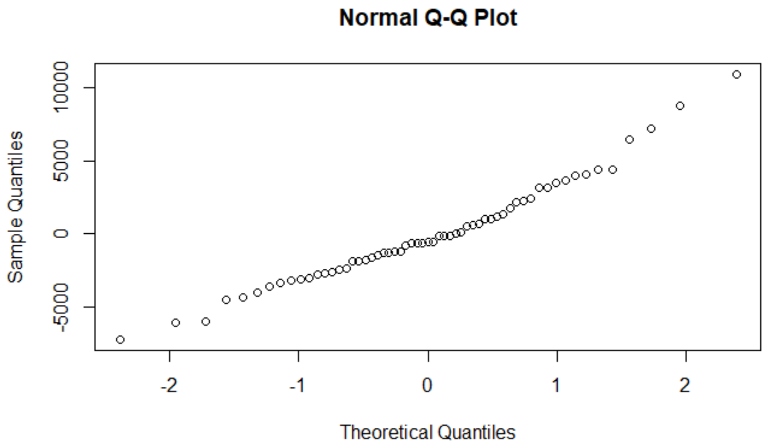

- **Figure 1G: Mean mEPSC amplitudes for each condition**

As the number of experiments is equal to ten (see below), a linear mixed effects model with a random intercept at the experiment level has been used (Yu et al., 2022).

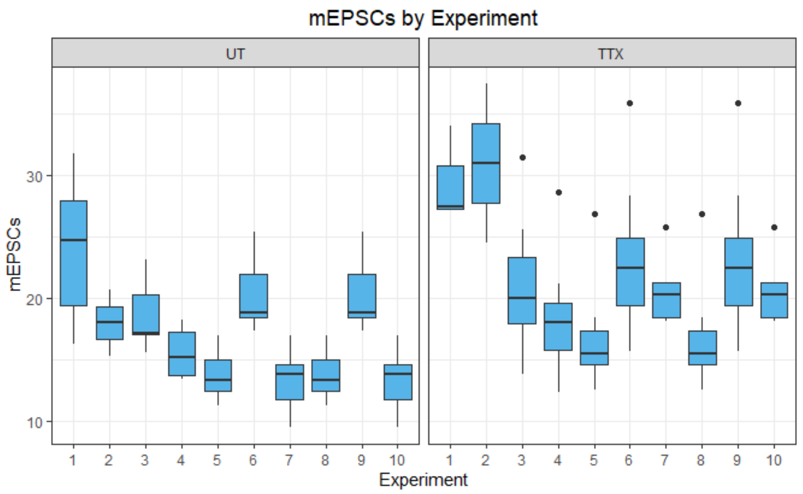

A log link function has been used to get a better normal distribution of the response variable "mEPSC amplitude" (see below).

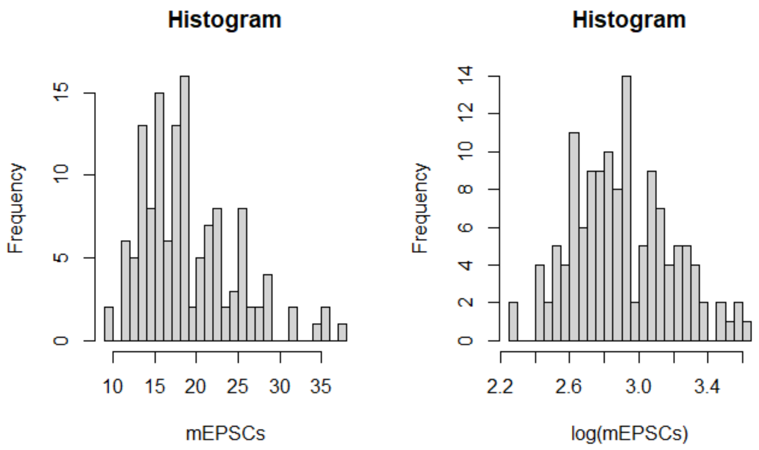

The results of the linear mixed-effects model show a significant difference between the mean of the mEPSC amplitude in untreated (UT) *versus* TTX-treated condition (regression coefficient = 0.21, p-value=0 ***).

The log link function doesn't change the sense of the association. Since the regression coefficient is positive, it indicates that the mean level of mEPSCs is more important for TTX-treated than untreated condition (UT).

Additionally, we have plotted the standardized residual *versus* the fitted values to evaluate the model. As seen below, there are as much positive residuals as negative residuals, showing the goodness of fit of the model.

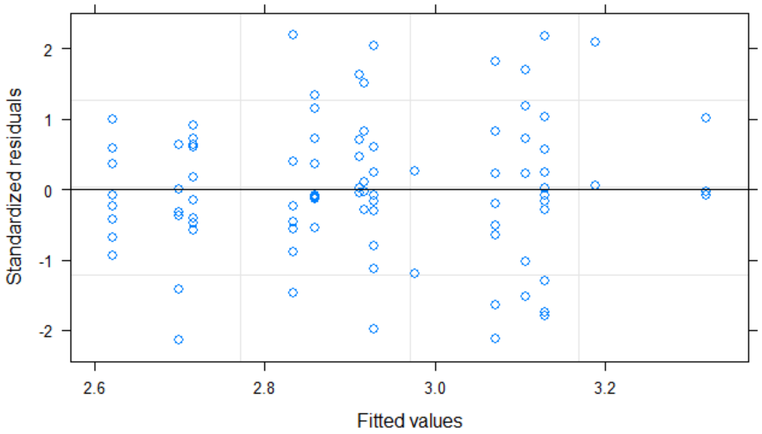

As a linear mixed-effects model has been used, we estimated the value of the ICC (Intra Class Correlation coefficient) and it was equal to 0.42, suggesting a high level of correlation at the experiment level. And therefore, the necessity of using a mixed-effects model in this case.

References:

Harrison, X. A., Donaldson, L., Correa-Cano, M. E., Evans, J., Fisher, D. N., Goodwin, C. E. D., Robinson, B. S., Hodgson, D. J., & Inger, R. (2018). A brief introduction to mixed effects modelling and multi-model inference in ecology. *PeerJ*, *6*(5), e4794. https://doi.org/10.7717/peerj.4794

Yu, Z., Guindani, M., Grieco, S. F., Chen, L., Holmes, T. C., & Xu, X. (2022). Beyond t test and ANOVA: applications of mixed-effects models for more rigorous statistical analysis in neuroscience research. *Neuron*, *110*(1), 21–35. https://doi.org/10.1016/j.neuron.2021.10.030

Referee #2:

In this revised manuscript, the authors have made several improvements, and addressed many of my previous criticisms.

I am still concerned about the data presented in figure 5C/D, and the conclusions drawn from them. I acknowledge that it makes sense for there to be an increase in synaptic AMPARs and Homer with TTX, given the increase in mEPSC amplitude, however it is still inappropriate to refer to any effect here. The spread of the data points clearly shows there is no detectable effect of TTX, nor of miR-124, on synaptic AMPARs. The authors offer some explanations for the discrepancy, the first of which they should test experimentally. I.e., how does the analysis look when SP- and SP+ synapses are considered separately? I don't agree with the authors' second point at all.

We acknowledge that the data presented in figure 5C and 5D do not allow to conclude on the effect of TTX on synaptic AMPARs or Homer1c signal. To address referee's concern about the data presented in Figure 5C and 5D, we decided to perform:

- **A refined synaptic analysis on AMPAR immunostaining, with SP- and SP+ synapses considered separately, as requested by the referee.** In neurons transfected with miR-Ctrl,

we find that SP+ but not SP- synapses show a robust increase in synaptic GluA immunosignal upon TTX treatment, supporting non-uniform HSP. This is in contrast with neurons transfected with miR-124, in which both SP- and SP+ synapses fail to undergo HSP while showing higher synaptic GluA immunosignal compared to miR-Ctrl in basal conditions. Therefore, in contrast to the initial analysis at the cell level which included both SP- and SP+ synapses, this new analysis further illustrates the differential ability of SP+ vs SP- synapses to undergo HSP.

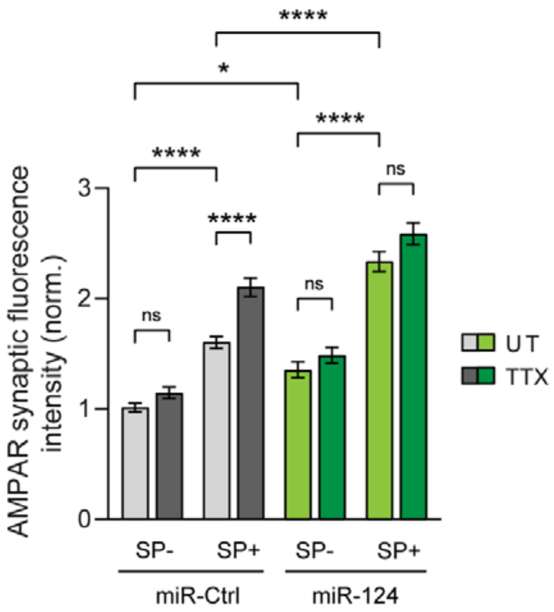

AMPAR synaptic fluorescence intensity for SP+ versus SP- synapses in in neurons transfected with miR-124 or control miR-67 (miR-Ctrl), and treated with TTX, or left untreated (UT). AMPAR synaptic fluorescence intensity was normalized to SP- from miR-Ctrl, UT condition. (n = 353 synapses for each condition, from 2 cultures). *P < 0.05, ****P < 0.0001, ns, not significant, P > 0.05 (two-way ANOVA test followed by Tukey's multiple comparison test).

- **One additional experiment (3 cultures in total) in order to increase the number of data points in Figure 5B-D).** These new data validate both the effect of the TTX treatment and the effect of overexpressing miR-124 on synaptic AMPARs content and now replace initial data in Figure 5. Please note that the effect on synapse size (Homer1c-GFP fluorescence intensity, Fig. 5D) does not reach statistical significance, which might be explained by higher cell-to-cell variability resulting from the exogenous expression of Homer1c-GFP.

Together, these new analysis/data suggest that miR-124 overexpression increases the abundance of synaptic AMPARs in basal condition, most likely through inhibiting GluA2 expression and promoting the assembly of Ca^{2+} permeable AMPARs (as discussed in the manuscript, pp. 8 and 16). Importantly, miR-124 overexpression inhibits HSP as well as the increase in the number of SP+ synapses. Together with loss-of-function experiments (Fig. 6), these results suggest that derepression of SP translation by miR-124 is required for HSP.

Referee #3:

In the revised version of the manuscript by Dubes and colleagues, the authors convincingly addressed all my points. They included additional experiments and controls, explained analysis pipeline and improved manuscript flow and conclusions. This is a very nice work and

I would recommended it to be accepted. There is only minor thing to be improved. Although the number of spines, cell and independent experiments have been added in the main figures, it is still missing in some panels in EV1. Please include it.

We thank the referee for his/her positive comments. We now provide the requested information for panels of figure EV1.

2nd Revision - Editorial Decision 27th Jun 2022

Dear Matheiu,

Thank you for submitting your revised manuscript to The EMBO Journal. Your study has now been seen by referees #1 and 2. As you can see from the comments below, both referees appreciate the introduced changes.

I am therefore very pleased to accept the MS for publication here.

Congratulations on a nice study!

PS => please take a look at the attached synopsis image. We moved it a bit around as the image was too wide. Will this work for you or do you prefer to send me another version [550 wide by [200-400] high (pixels)]?

With best wishes

Karin

Karin Dumstrei, PhD
Senior Editor
The EMBO Journal

Referee #1:

The authors have now satisfactorily addressed my remaining concern. I can therefore recommend publication.

Referee #2:

The authors have satisfactorily addressed all my concerns.
This is a great paper, and should be published.

****

Please note that it is EMBO Journal policy for the transcript of the editorial process (containing referee reports and your response letter) to be published as an online supplement to each paper. If you do NOT want this, you will need to inform the Editorial Office via email immediately. More information is available here:
https://www.embopress.org/page/journal/14602075/authorguide#transparentprocess

Your manuscript will be processed for publication in the journal by EMBO Press. Manuscripts in the PDF and electronic editions of The EMBO Journal will be copy edited, and you will be provided with page proofs prior to publication. Please note that supplementary information is not included in the proofs.

You will be contacted by Wiley Author Services to complete licensing and payment information. The required 'Page Charges Authorization Form' is available here: https://www.embopress.org/pb-assets/embo-site/tej_apc.pdf - please download and complete the form and return to embopressproduction@wiley.com

Should you be planning a Press Release on your article, please get in contact with embojournal@wiley.com as early as possible, in order to coordinate publication and release dates.

If you have any questions, please do not hesitate to call or email the Editorial Office. Thank you for your contribution to The EMBO Journal.

** Click here to be directed to your login page: https://emboj.msubmit.net

EMBO Press Author Checklist

Corresponding Author Name: Mathieu LETELLIER	
Journal Submitted to: The EMBO Journal	
Manuscript Number: EMBOJ-2021-109012R	

USEFUL LINKS FOR COMPLETING THIS FORM
The EMBO Journal - Author Guidelines
EMBO Reports - Author Guidelines
Molecular Systems Biology - Author Guidelines
EMBO Molecular Medicine - Author Guidelines

Reporting Checklist for Life Science Articles (updated January 2022)
This checklist is adapted from Materials Design Analysis Reporting (MDAR) Checklist for Authors. MDAR establishes a minimum set of requirements in transparent reporting in the life sciences (see Statement of Task: 10.31222/osf.io/9sm4x). Please follow the journal's guidelines in preparing your manuscript.
Please note that a copy of this checklist will be published alongside your article.

Abridged guidelines for figures
1. Data
The data shown in figures should satisfy the following conditions:
- the data were obtained and processed according to the field's best practice and are presented to reflect the results of the experiments in an accurate and unbiased manner.
- ideally, figure panels should include only measurements that are directly comparable to each other and obtained with the same assay.
- plots include clearly labeled error bars for independent experiments and sample sizes. Unless justified, error bars should not be shown for technical replicates.
- if n<5, the individual data points from each experiment should be plotted. Any statistical test employed should be justified.
- Source Data should be included to report the data underlying figures according to the guidelines set out in the authorship guidelines on Data Presentation.

2. Captions
Each figure caption should contain the following information, for each panel where they are relevant:
- a specification of the experimental system investigated (eg cell line, species name).
- the assay(s) and method(s) used to carry out the reported observations and measurements.
- an explicit mention of the biological and chemical entity(ies) that are being measured.
- an explicit mention of the biological and chemical entity(ies) that are altered/varied/perturbed in a controlled manner.
- the exact sample size (n) for each experimental group/condition, given as a number, not a range;
- a description of the sample collection allowing the reader to understand whether the samples represent technical or biological replicates (including how many animals, litters, cultures, etc.).
- a statement of how many times the experiment shown was independently replicated in the laboratory.
- definitions of statistical methods and measures:
  - common tests, such as t-test (please specify whether paired vs. unpaired), simple χ^2 tests, Wilcoxon and Mann-Whitney tests, can be unambiguously identified by name only, but more complex techniques should be described in the methods section;
  - are tests one-sided or two-sided?
  - are there adjustments for multiple comparisons?
  - exact statistical test results, e.g., P values = x but not P values < x;
  - definition of 'center values' as median or average;
  - definition of error bars as s.d. or s.e.m.

Please complete ALL of the questions below.
Select "Not Applicable" only when the requested information is not relevant for your study.

Materials

Newly Created Materials	Information included in the manuscript?	In which section is the information available? (Reagents and Tools Table, Materials and Methods, Figures, Data Availability Section)
New materials and reagents need to be available; do any restrictions apply?	Yes	No restriction applies regarding the availability of the new DNA plasmids or probes generated in the study.

Antibodies	Information included in the manuscript?	In which section is the information available? (Reagents and Tools Table, Materials and Methods, Figures, Data Availability Section)
For **antibodies** provide the following information:<br>- Commercial antibodies: RRID (if possible) or supplier name, catalogue number and or/clone number<br>- Non-commercial: RRID or citation	Yes	References for all antibodies are provided in the Materialsl and Methods as well as Appendix Table S2.

DNA and RNA sequences	Information included in the manuscript?	In which section is the information available? (Reagents and Tools Table, Materials and Methods, Figures, Data Availability Section)
**Short novel DNA or RNA including primers, probes**: provide the sequences.	Yes	The sequences are given in the relevant sections in Materials and Methods.

Cell materials	Information included in the manuscript?	In which section is the information available? (Reagents and Tools Table, Materials and Methods, Figures, Data Availability Section)
**Cell lines:** Provide species information, strain. Provide accession number in repository **OR** supplier name, catalog number, clone number, and/**OR** RRID.	Yes	COS-7 cells were supplied by EACC (87021302). HEK-293 cells were purchased from EACC (85120602).
**Primary cultures:** Provide species, strain, sex of origin, genetic modification status.	Yes	This information is available in the Materials and Methods section.
Report if the cell lines were recently **authenticated** (e.g., by STR profiling) and tested for mycoplasma contamination.	Yes	Cell lines were tested for mycoplasma contamination.

Experimental animals	Information included in the manuscript?	In which section is the information available? (Reagents and Tools Table, Materials and Methods, Figures, Data Availability Section)
**Laboratory animals or Model organisms:** Provide species, strain, sex, age, genetic modification status. Provide accession number in repository **OR** supplier name, catalog number, clone number, **OR** RRID.	Yes	This information is available in the Materials and Methods section.
**Animal observed in or captured from the field:** Provide species, sex, and age where possible.	Not Applicable	
Please detail **housing and husbandry conditions**.	Yes	This information is available in the Materials and Methods section (organotypic slices).

Plants and microbes	Information included in the manuscript?	In which section is the information available? (Reagents and Tools Table, Materials and Methods, Figures, Data Availability Section)
**Plants:** provide species and strain, ecotype and cultivar where relevant, unique accession number if available, and source (including location for collected wild specimens).	Not Applicable	
**Microbes:** provide species and strain, unique accession number if available, and source.	Not Applicable	

Human research participants	Information included in the manuscript?	In which section is the information available? (Reagents and Tools Table, Materials and Methods, Figures, Data Availability Section)
If collected and within the bounds of privacy constraints report on age, sex and gender or ethnicity for all study participants.	Not Applicable	

Core facilities	Information included in the manuscript?	In which section is the information available? (Reagents and Tools Table, Materials and Methods, Figures, Data Availability Section)
If your work benefited from core facilities, was their service mentioned in the acknowledgments section?	Yes	This is mentioned in the acknowledgement section

Design

Study protocol	Information included in the manuscript?	In which section is the information available? (Reagents and Tools Table, Materials and Methods, Figures, Data Availability Section)
If study protocol has been **pre-registered, provide DOI in the manuscript**. For clinical trials, provide the trial registration number **OR** cite DOI.	Not Applicable	
Report the **clinical trial registration number** (at ClinicalTrials.gov or equivalent), where applicable.	Not Applicable	

Laboratory protocol	Information included in the manuscript?	In which section is the information available? (Reagents and Tools Table, Materials and Methods, Figures, Data Availability Section)
Provide DOI OR other citation details if **external detailed step-by-step protocols** are available.	Not Applicable	

Experimental study design and statistics	Information included in the manuscript?	In which section is the information available? (Reagents and Tools Table, Materials and Methods, Figures, Data Availability Section)
Include a statement about **sample size** estimate even if no statistical methods were used.	Yes	No statistical method was used to pre-determine sample size; however, the sample size per experiment was based on those reported earlier by us and others. Sample sizes are indicated in Figure legends.
Were any steps taken to minimize the effects of subjective bias when allocating animals/samples to treatment (e.g. **randomization procedure**)? If yes, have they been described?	Yes	The samples were randomly allocated into the different experimental groups (treatment and/or genetic manipulations).
Include a statement about **blinding** even if no blinding was done.	Yes	Allocation of samples to experimental groups as well as data analysis were performed blind whenever possible.
Describe **inclusion/exclusion criteria** if samples or animals were excluded from the analysis. Were the criteria pre-established?<br><br>If sample or data points were omitted from analysis, report if this was due to attrition or intentional exclusion and provide justification.	Yes	Some neuronal cultures were excluded based on abnormal neuronal density or poor cell viability/health (as assessed based on morphological and/or electrophysiological criteria). Data points outliers were indentified and excluded using the ROUTmethod on raw experimental data.
For every figure, are **statistical tests** justified as appropriate? Do the data meet the assumptions of the tests (e.g., normal distribution)? Describe any methods used to assess it. Is there an estimate of variation within each group of data? Is the variance similar between the groups that are being statistically compared?	Yes	All analyses were performed using GraphPad Prism 8 and assessed for normality and variance, when appropriate. This is described in the Materials and Methods section.

Sample definition and in-laboratory replication	Information included in the manuscript?	In which section is the information available? (Reagents and Tools Table, Materials and Methods, Figures, Data Availability Section)
In the figure legends: state number of times the experiment was **replicated** in laboratory.	Yes	This is indicated in the Figure legends.
In the figure legends: define whether data describe **technical or biological replicates**.	Yes	This is indicated in the Figure legends.

Ethics

Ethics	Information included in the manuscript?	In which section is the information available? (Reagents and Tools Table, Materials and Methods, Figures, Data Availability Section)
Studies involving **human participants**: State details of **authority granting ethics approval** (IRB or equivalent committee(s), provide reference number for approval.	Not Applicable	
Studies involving **human participants**: Include a statement confirming that **informed consent** was obtained from all subjects and that the experiments conformed to the principles set out in the WMA Declaration of Helsinki and the Department of Health and Human Services Belmont Report.	Not Applicable	
Studies involving **human participants:** For publication of **patient photos**, include a statement confirming that consent to publish was obtained.	Not Applicable	
Studies involving experimental **animals**: State details of **authority granting ethics approva**l (IRB or equivalent committee(s), provide reference number for approval. Include a statement of compliance with ethical regulations.	Yes	This statement appears in the Material and Methods section.
Studies involving **specimen and field samples:** State if relevant **permits** obtained, provide details of authority approving study; if none were required, explain why.	Not Applicable	

Dual Use Research of Concern (DURC)	Information included in the manuscript?	In which section is the information available? (Reagents and Tools Table, Materials and Methods, Figures, Data Availability Section)
Could your study fall under dual use research restrictions? Please check biosecurity documents and list of **select agents and toxins** (CDC): https://www.selectagents.gov/sat/list.htm	Not Applicable	
If you used a select agent, is the security level of the lab appropriate and reported in the manuscript?	Not Applicable	
If a study is subject to dual use research of concern regulations, is the name of the **authority granting approval and reference number** for the regulatory approval provided in the manuscript?	Not Applicable	

Reporting

The MDAR framework recommends adoption of discipline-specific guidelines, established and endorsed through community initiatives. Journals have their own policy about requiring specific guidelines and recommendations to complement MDAR.

Adherence to community standards	Information included in the manuscript?	In which section is the information available? (Reagents and Tools Table, Materials and Methods, Figures, Data Availability Section)
State if relevant guidelines or checklists (e.g., **ICMJE, MIBBI, ARRIVE, PRISMA**) have been followed or provided.	Yes	Our study complies with ICMJE guidelines.
For **tumor marker prognostic studies,** we recommend that you follow the **REMARK** reporting guidelines (see link list at top right). See author guidelines, under 'Reporting Guidelines'. Please confirm you have followed these guidelines.	Not Applicable	
For **phase II and III randomized controlled trials**, please refer to the **CONSORT** flow diagram (see link list at top right) and submit the CONSORT checklist (see link list at top right) with your submission. See author guidelines, under 'Reporting Guidelines'. Please confirm you have submitted this list.	Not Applicable	

Data Availability

Data availability	Information included in the manuscript?	In which section is the information available? (Reagents and Tools Table, Materials and Methods, Figures, Data Availability Section)
Have **primary datasets** been deposited according to the journal's guidelines (see 'Data Deposition' section) and the respective accession numbers provided in the Data Availability Section?	Not Applicable	
Were **human clinical and genomic datasets** deposited in a public access-controlled repository in accordance to ethical obligations to the patients and to the applicable consent agreement?	Not Applicable	
Are **computational models** that are central and integral to a study available without restrictions in a machine-readable form? Were the relevant accession numbers or links provided?	Not Applicable	
If publicly available data were reused, provide the respective **data citations in the reference list**.	Not Applicable	
